# In Vitro Bioassay Evidence for Chemical Mixture Propagation from the Environment to Humans

**DOI:** 10.1021/acs.est.6c00908

**Published:** 2026-06-02

**Authors:** Beate Escher, Martin Scholze, Maria Margalef, Maria König, Maria J. Valente, Timo Hamers, Kostja Renko, Marc Audebert, Jungeun Lee, Laure Khoury, Peter Cenijn, Yanying Ma, Andreas Frederik Treschow, Leisa-Maree Toms, Christina Rørbye, Georg Braun, Solène Motteau, Jean-Philippe Antignac, Gaud Dervilly, Marja Lamoree, Anne Marie Vinggaard

**Affiliations:** 1 Department of Cell Toxicology, Helmholtz Centre for Environmental Research − UFZ, Leipzig 04318, Germany; 2 Department of Geosciences, Eberhard Karls University of Tübingen, Schnarrenbergstr. 94-96, 72076 Tübingen, Germany; 3 German Center for Child and Adolescent Health (DZKJ), partner site Leipzig/Dresden, Leipzig 04103, Germany; 4 Centre for Pollution Research and Policy, Brunel University London, Kingston Lane, Uxbridge UB8 3PH, U.K.; 5 Amsterdam Institute for Life and Environment, Faculty of Science, Vrije Universiteit Amsterdam, 1081 HV Amsterdam, The Netherlands; 6 National Food Institute, Technical University of Denmark, DK-2800 Kgs. Lyngby, Denmark; 7 German Federal Institute for Risk Assessment (BfR), German Centre for the Protection of Laboratory Animals (Bf3R), 10589 Berlin, Germany; 8 Toxalim, Université de Toulouse, INRAE,INP-ENVT, INP-EI-Purpan, Université de Toulouse 3 Paul Sabatier, 31062 Toulouse, France; 9 PrediTox, 31100 Toulouse, France; 10 School of Public Health and Social Work, Faculty of Health, Queensland University of Technology, Brisbane, QLD 4000, Australia; 11 Department of Obstetrics and Gynaecology, Hvidovre University Hospital, 2650 Hvidovre, Denmark; 12 LABERCA, Oniris, INRAE, 44307 Nantes, France

**Keywords:** new approach methodologies, adverse outcome pathway, in vitro bioassay, mixture effects, concentration addition, iceberg modeling

## Abstract

Complex mixtures of organic chemicals extracted from representative but not directly related environmental samples (wastewater, surface water, fish), food items (drinking water, fish, milk) and human blood were tested in 22 in vitro bioassays targeting pathways associated with neurodevelopmental and reproductive health. Extraction methods were optimized to extract common chemicals across matrices capturing both persistent and nonpersistent, neutral and charged organic chemicals—albeit with some bias toward more hydrophilic chemicals over highly hydrophobic chemicals. Most bioassay end points—except genotoxicity—were responsive, with strongest effects observed higher up the food chain in fish and humans. Experimental mixture effects of 24 chemicals quantified in these extracts conformed to the mixture prediction model of concentration addition in the six most responsive bioassays, namely neurite outgrowth inhibition, mitochondrial membrane potential inhibition, transthyretin protein binding, sodium-iodide symporter inhibition and androgen receptor antagonism. Designed mixtures explained little of total bioactivity, indicating that many of the thousands of unannotated molecular features detected by nontarget analysis contribute to mixture effects. Preliminary effect-based trigger (EBT) values defined for water and food by extrapolation from safe levels of individual chemicals indicate no immediate health risks at these average contamination levels. The high complexity and multivalent bioactivity of these mixtures on neurodevelopmental and reproductive pathways necessitate further toxicological scrutiny.

## Graphical abstract

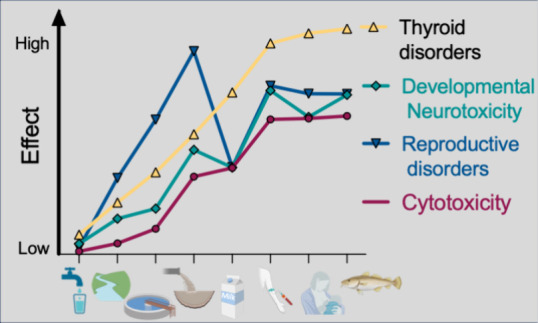

## Introduction

Synthetic organic chemicals are ubiquitous in the environment. Complex mixtures may contain thousands of chemicals, each with the potential to contribute to a wide range of toxic effects. Regulatory efforts are challenged by the sheer number, diversity, and co-occurrence of these chemicals, making it difficult to assess their risks to environmental and human health. Our understanding of how such mixtures—especially at low concentrations—impact human health remains limited. In vitro bioassays are particularly well-suited for studying both complex mixtures of unknown composition and designed mixtures with known constituents. When combined with chemical analysis, they provide powerful tools for evaluating mixture effects and identifying key chemical drivers of toxicity across environmental, food, and human samples.

The Adverse Outcome Pathway (AOP) framework is a valuable tool for selecting bioassays linked to adverse health outcomes.^1^ AOPs describe how molecular initiating events (MIEs) trigger a cascade of key events (KEs), ultimately leading to adverse outcomes. While initially conceptualized as linear sequences, AOPs are now seen as complex, interconnected networks.^2^ For single chemical risk assessment, understanding the full AOP is ideal,^3^ but for screening complex mixtures, it is more convenient to use representative bioassays aligned with relevant MIEs and KEs that are associated with the adverse outcome. In this study, we were particularly interested in key toxicological domains relevant to child development, including developmental neurotoxicity, thyroid hormone system disruption, reproductive toxicity, and genotoxicity. Bioassay selection was guided by putative AOPs synthesized from diverse literature sources, providing a strong toxicological rationale for coverage of outcomes. A test battery comprising 22 in vitro bioassays was initially applied to all samples but designed mixture studies were intentionally reduced to a subset of seven bioassays, selected based on responsiveness, specificity, and suitability for mixture modeling.

Diverse samples spanning the environment-food-human continuum were extracted using dedicated methods designed to simultaneously capture a broad range of organic chemicals across various chemical classes, including hydrophobic and hydrophilic compounds as well as neutral and charged organic chemicals. The extracted chemicals comprise both exogenous and endogenous organic chemicals in fish, milk and humans, which cannot be separated due to their similar physicochemical properties. Some dissolved organic matter is typically coextracted in water by the chosen solid-phase extraction method but this residual has been shown not to interfere with cell-based bioassays.^4^ For fish, milk and human matrices, lipids and proteins were largely removed by the extraction methods as described in detail in the experimental section. Metals and inorganic compounds were excluded by the extraction methods applied, the solid-phase extraction (SPE)- and solvent-based extraction procedures used in this study were designed for organic chemicals and are not suitable for simultaneous extraction of metals and organic contaminants. The focus of this study was on the high diversity of the organic chemical universe.^5^ Metals can be comprehensively characterized by conventional metal analysis and were outside the scope of the present bioassay-based assessment.

As hundreds of thousands of synthetic organic chemicals are in commercial use, it remains largely unknown, how many and how much of them and their transformation products occur in the environment and humans.^6^ Consequently, quantified target chemicals and their modeled mixture effects represent only the visible “tip of the iceberg” and do not capture the total effect of all active chemicals including those in the invisible “bottom of the iceberg”. Effect-based measurements of whole extracts provide an integrative assessment of the combined biological activity of known and unknown chemicals in complex mixtures.^7^

Most in vitro mixture studies reported in the literature have used equipotent mixture ratios, while only a few have investigated reconstituted mixtures based on their actual occurrence in environmental or human samples.^8,9^ One such example is the mixture of persistent organic pollutants developed by Berntsen et al.,^10^ which has been widely adopted in subsequent studies,^11–13^ but individual components of these mixtures were not tested.

The central paradigm of mixture toxicity holds that chemicals sharing the same target site or mode of action (MOA) and which do not interact, act additively in accordance with the principle of concentration addition (CA). This assumption is well-accepted for MIE-related end points and is considered a reasonable default for KE-related end points, supported by ample empirical evidence from environmental and human mixture scenarios.^14,15^ CA predicted effects well for environmentally realistic exposure scenarios if they were measured with MOA-specific bioassays^16,17^ or mixtures were complex and only investigated at low effect levels,^14,15^ where interactions between chemicals are less likely and CA-predictions overlap with independent action.^17^

We extracted pooled samples with the aim of obtaining an average representative signal per sample type, rather than focusing on outliers from hot spots or particularly vulnerable populations. Five types of water samples pooled from ten European countries were extracted to represent key exposure sources. Wastewater treatment plant influent and effluent served as a model for human waste outputs and as major emission sources of pollutants into the environment. Surface water was selected to represent an important environmental compartment, with fish serving both as representative aquatic organisms and as components of human food. For this purpose, we purchased three species of fish each in fish markets in seven European countries. Terrestrial mammals’ exposure was reflected by samples of both conventional and organic cow milk purchased in eight countries. Infants’ exposure was assessed through breast milk, although the number of samples available for pooling was limited to six. Drinking water, bottled water, milk and fish collectively represented part of the human food basket, providing insight into dietary exposure. Adult serum and umbilical cord serum stemmed from individual countries and constituted mixtures prepared by local biobanks. Not only these 16 extracts were tested with the bioassay battery but also mixtures of 24 chemicals reconstituted in the detected concentration ratios.

## Materials and Methods

### Sample Collection and Extraction

An overview of all sample types and their sample code is given in Table S1 and Figure 1A. Details on the optimization/harmonization of the extraction methods are in Text S1.

**Figure 1:**
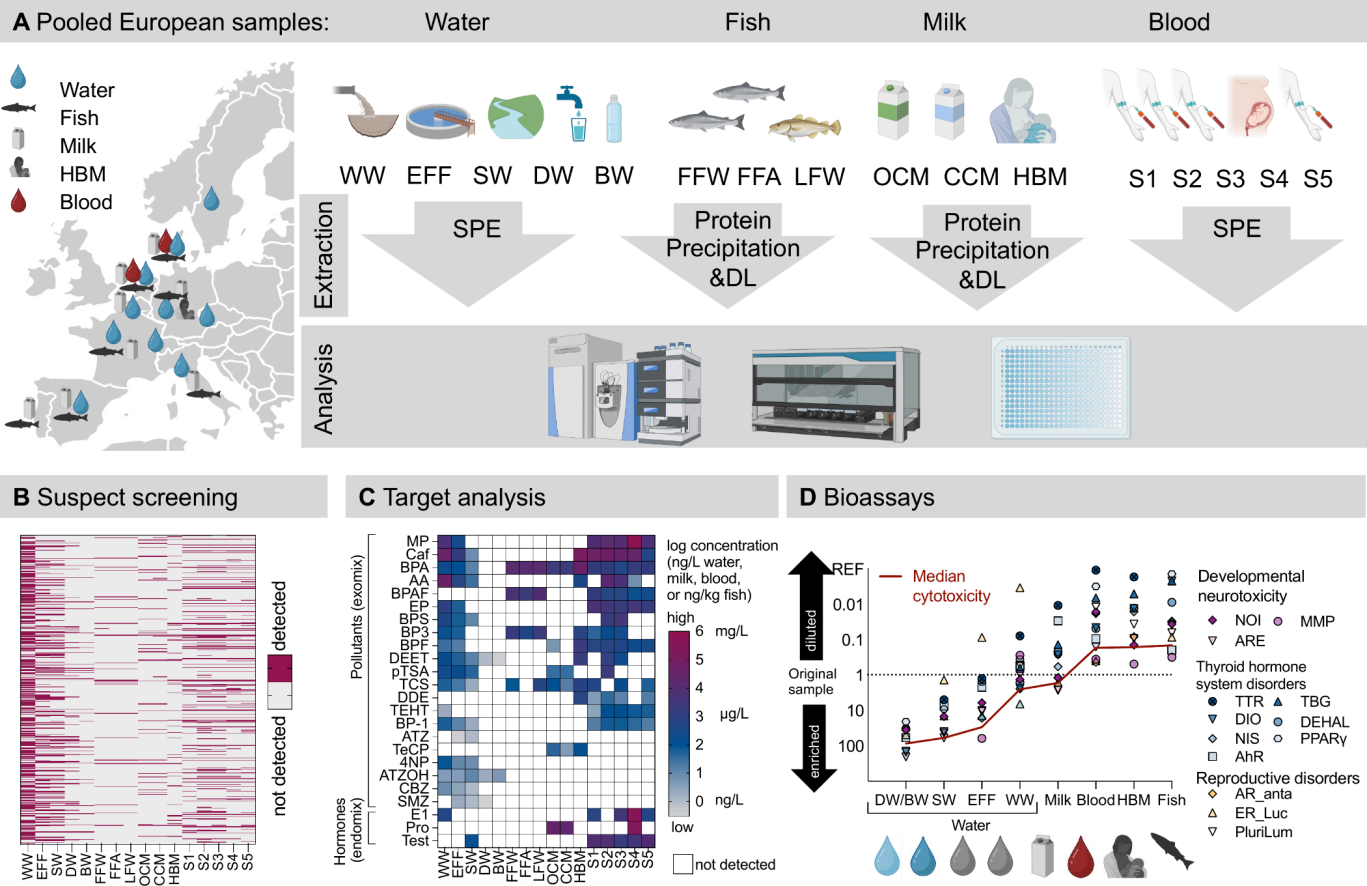
Overview on the samples in the continuum from environment via food to humans and results from chemical analysis and bioassays. (A) Origin of samples sample code (wastewater treatment plant influent (WW), effluent (EFF), surface water (SW), drinking water from the tap (DW), bottled water (BW), fat fish wild (FFW) and from aquaculture (FFA), lean fish (LFW), organic cow milk (OCM), conventional cow milk (CCM), human breastmilk (HBM), serum samples (S1 to S5)), extraction methods (solid-phase extraction (SPE) or protein precipitation and delipidation (DL)), and analysis methods (nontarget and target analysis and bioassays). Figure partially created in BioRender. Escher, B. (2025) https://BioRender.com, wdf1cxx and tm1aov5. (B) Features detected in the extracts with suspect screening (data from Motteau et al.^19^). (C) Estimated concentrations of 24 chemicals detected in >3 extracts (Table S3). Full names and abbreviations of chemicals are listed in Table S3. (D) Overview on the relative effects (concentration causing 10% effect (1/EC_10_) or induction ratio 1.5 (1/EC_IR1.5_) in units of relative enrichment factor, Table S5) of the samples (means for fish, milk and serum) in all bioassay (Table S4) sorted according to increasing mean cytotoxicity (concentration causing 10% cytotoxicity 1/IC_10_). For clarity of visualization, AChE, AR agonism, and genotoxicity are not shown in the plot, as only few samples were active in these assays. However, all results are given in Table S5, and detailed data are given in Tables S6–S26. For the iodothyronine deiodinase assays, the mean of DIO1, DIO2, and DIO3 is reported. Abbreviations: NOI, neurite outgrowth inhibition; ARE, oxidative stress response via the keap-Nrf2-ARE pathway; MMP, mitochondrial toxicity via mitochondrial membrane potential inhibition; THR-TA, thyroid hormone receptor transactivation assay; TTR, competition with FITC-T4 for transthyretin protein binding; TBG, competition with FITC-T4 for thyroxine-binding globulin (TBG); DIO, iodothyronine deiodinase; DEHAL, recombinant dehalogenase 1 (iodotyrosine deiodinase activity (iodine recycling)); NIS, sodium-iodide-symporter inhibition; AhR, activation of the arylhydrocarbon receptor; PPARγ, activation of the peroxisome proliferator activated receptor; AR_ago, androgen receptor transcriptional activation assay (agonist mode); AR_antagonism, AR (antagonist mode); ER_Luc, estrogen receptor (ER) transcriptional activation assay; ER_BLA, ER transcriptional activation assay GeneBLAzer; PluriLum, 3D embryoid bodies made from hIPSC.

Water samples, including wastewater treatment plant influent (WW), effluent (EFF), surface water (SW), drinking water from the tap (DW), bottled water (BW), were collected and extracted in 10 countries (Germany (Leipzig), The Netherlands (SW Rhenen, WW Nieuwegein, DW Amsterdam), Denmark (Kongens Lyngby), France (Nantes), Norway (Trondheim), Czech Republic (Budějovice), Switzerland (Dübendorf), Spain (Girona), Italy (Ispra), Belgium (Ghent)). Solid-phase extraction with the hydrophobic polystyrene-divinylbenzene resin HR-X (CHROMABOND HR-X 6 mL, 500 mg solid material, polypropylene cartridges, with polyethylene filter elements) was used to extract the water in a laboratory close to the collection site as soon as possible after sampling. A standard operating procedure was provided to all participating laboratories.^18^

Fish served as representative of an environmental organism but also as a food item and 18 marine fish were purchased in 7 European countries.^19^ They were grouped in wild salmon (*Salmo salar)* (fat fish wild, FFW) and salmon from aquaculture (fat fish aquaculture, FFA) and cod (*Gadus morhua*) or colefish (*Salvelinus colii*) (lean fish wild, LFW). The country where they were purchased is not necessarily the country where they were caught or bred in aquaculture. FFW was purchased in The Netherlands, Portugal and France, FFA was purchased in The Netherlands, Denmark, Germany, Portugal, France, Italy, Spain, LFW was purchased in The Netherlands, Denmark, Germany (cod and colefish), Portugal, France, Italy, Spain.

Cow milk served as representative of mammalian exposure and human food source. Full-fat conventional and organic cow milk were sourced from 8 European countries (The Netherlands, Denmark, Germany, Belgium, Portugal, France, Italy and Spain).^19^ Cow milk was extracted by liquid–liquid extraction of acetonitrile followed by delipidation. Pooled breast milk samples (*n* = 6, 300 mL) from Germany were extracted with the same method as for fish samples, with pure acetonitrile as extraction solvent.^19^

Mixed 250 mL serum were obtained from five different world regions.^19^ Unfortunately, difficulty to source adequate volumes of serum made it impossible to be representative for several European Countries and the European sample set was supplemented by serum from Australia, due to availability and similarity of socio-economic factors. The Medical Ethics Review Committee of the Vrije Universiteit Medical Centre confirmed that this study was not subject to the Dutch Medical Research Involving Human Subjects Act (WMO, reference number 2022.0119). At the same time, ethical approval or exemption for approval was also warranted by each provider when needed, and participants provided written informed consent to their respective national biobanks according to national regulations. S1 was serum from Healthy Adults (M/F) sourced from the Blood bank at Rigshospitalet, Denmark (anonymized, approval letter by Morte Bagge Hansen of 2022/02/15). S2 and S3 were both sourced in Australia from healthy Australian Adults (M/F) and healthy women of child-bearing age, respectively (ethics approval: 2013/HE000317, HABS LNR, The University of Queensland, Brisbane QLD 4072 Australia). Umbilical cord serum was collected from the Danish National Biobank collected at the Center for Regional Udvikling, Hvidovre Hospital, Denmark (reference number 22010527). S5 was pooled from healthy European Adults (M/F) sourced from different countries in Europe (undisclosed European countries, The Netherlands and Spain). S5 samples were provided by BioIVT (commercial supplier), Sanquin, a not-for-profit blood bank, and the IBSP-CV Biobank, integrated in the Spanish National Biobank Network and in the Valencian Biobanking Network (ethics approval: dictamen CEI 20220225/07, Fundació per al Foment de la Investigació Sanitària i Biomèdica de la Comunitat Valenciana (FISABIO)).

BSSPE and BS5SPE were Milli-Q water process blanks and BCBB was a blank of Milli-Q Water extracted after 10 min in blood collection bags/tubes. Charcoal-stripped fetal bovine serum (cs-FBS), obtained as described by Horwitz et al.^20^ was extracted as a matrix background. Serum samples were extracted with 20 cc Oasis HLB cartridges (OASIS HLB 20 cc 1 g, 60 μm particles, #186000117, Waters, Eschborn, Germany) according to a method modified from Simon et al.^21^

No extraction recovery corrections were made for chemical analysis and bioassays. All extracts were shipped in dry form to the bioassay and analytical laboratories.

### Dose Metrics

The extraction factor EF (eq 1) is defined as the mass or volume of sample extracted per final volume of extract. The extracts were dosed to the bioassays by adding a certain small volume to the bioassays described by the dosing factor DF (eq 2). The multiplication of EF and DF lead to the relative extraction factor REF, which is the dose-metric in the bioassays (eq 3).

1 $$\mathrm{Extraction factor (EF)}\;\left[\frac{g_{\mathrm{matrix}}\;\mathrm{or}\;{\mathrm{mL}}_{\mathrm{water}/\mathrm{milk}/\mathrm{serum}}}{L_{\mathrm{extract}}}\right]=\frac{\mathrm{mass or volume extracted}}{\mathrm{final volume of extract}}$$

2 $$\mathrm{Dosing factor (DF)}\;\left[\frac{L_{\mathrm{extract}}}{L_{\mathrm{bioassay}}}\right]=\frac{\mathrm{volume of extract dosed}}{\mathrm{final volume in bioassay}}$$

3 $$\mathrm{Relative extraction factor (REF)}\;\left[\frac{g_{\mathrm{matrix}}\;\mathrm{or}\;{\mathrm{mL}}_{\mathrm{water}/\mathrm{milk}/\mathrm{serum}}}{{\mathrm{mL}}_{\mathrm{bioassay}}}\right]=\frac{\mathrm{mass or volume extracted}}{\mathrm{final volume in bioassay}}=\mathrm{EF}\cdot \mathrm{DF}$$

### Chemical Analysis

For a set of 45 chemicals among those identified in the previous study,^19^ for which reference standards were available (Table S2), semiquantification was performed based on a mix of labeled internal standards (IS). Details in Text S2.

### Bioassays

Detailed descriptions of the bioassay methods are in Text S3 and a summary of all bioassays, cell lines and references is given in Table S4.

The acute zebrafish embryo (ZFE) toxicity assay was performed according to the modified OECD test guideline 236 ^22^ with morphological assessment of developmental effects.^23^ The ZFE assay has been previous used to test water extracts.^24^

The neurite outgrowth inhibition (NOI) assay uses human neuroblastoma SH-SY5Y cells, which has been already adapted for testing chemicals,^25,26^ environmental^27^ and human samples.^14^ Inhibitory effects on the acetylcholinesterase (AChE) activity were measured in SH-SY5Y cells according to a previously established method that had already been applied for water quality testing.^28^ The MitoOxTox assay is a multiplexed assay based on the AREc32 cell line that expresses luciferase stably under the antioxidant response element-driven NRF-2^29^ coupled to quantification of the mitochondrial membrane potential.^30^ It has been used for testing chemicals,^30^ designed mixtures,^31^ and extracts of water samples.^30^

The thyroid hormone receptor transactivation assay (THR-TA) is a reporter gene assay using a transfected cell line (GH3.TRE-Luc) that constitutively expresses both thyroid hormone receptor isoforms as described by Freitas et al.^32^ The TTR-binding assay was performed as described by Hamers et al.^8^ with some modifications in incubation time and temperature.^33^ The TTR assay has been used to identify TTR-active chemicals in water samples^34^ and serum of polar bears.^35^

The thyroxine-binding globulin (TBG) binding was performed according to Shen et al.^36^ Potential inhibition of the iodothyronine deiodinases type 1, 2, and 3 (DIO1, DIO2, DIO3) and the dehalogenase 1 (DEHAL), which is also named iodotyrosine deiodinase IYD, was tested in four individual enzymatic assays.^37^ These four cell-free assays were for the first time applied to testing of environmental and human extracts. The sodium-iodide-symporter (NIS) assay utilizes a FTC238-derived cell line with a functional human sodium-iodide-symporter fused to the reporter protein firefly luciferase (FLuc),^38^ and was applied for the first time for extract testing.

The AR-EcoScreen^39^ was used to quantify activation and antagonism of the androgen receptor. The GeneBLAzer PPARγ-UAS-bla 293H cells were used to quantify the activation of the peroxisome proliferator-activated receptor γ (PPARγ).^40^ The rat hepatoma cell line H4L7.5c2 was used in the AhR CALUX assay.^40,41^ The VM7Luc4E2 ER transactivation assay^42^ and the GeneBLAzer ERα-UAS-bla GripTite assay^40^ reported activation of the estrogen receptor. All these reporter gene assays or very similar ones have been widely used for water quality assessment,^43^ the AR, ER and AhR assays for testing of human samples^44^ and the AhR CALUX for fish extracts.^45^ The PluriLum assay is based on 3D embryoid bodies made from hIPSC differentiated to beating cardiomyocytes, transfected with a luciferase reporter gene to quantify the activation of the cardiac-specific homeobox gene NKX2.5, which indicates disturbance of heart development.^46,47^ It was applied for the first time to extract testing.

The γH2AX/pH3 assay was performed with the In-Cell Western (ICW) technique on the neuronal cell line SH-SY5Y and the metabolically competent cell line HepG2.^48,49^ While new in this set up, both cell lines have been dosed with extracts from water^43^ and SH-SY5Y with blood extracts.^14^

### Concentration–Response Modeling

All replicate bioassay measurements of one sample were evaluated together in one common concentration–response curve (CRC) because the number of technical and biological replicates was limited by the sample availability and was not consistent across assays. The raw data of the bioassays are given in Tables S6–S26 and S29–S35 and these tables indicate the number of technical and biological replicates. We harmonized the data evaluation as much as possible as outlined in a previous publication on the benchmarking of water quality with more than one hundred bioassays.^50^ Each plate included negative control wells (unexposed cells = 0% effect) and positive controls (a potent reference compound = 100%) to ensure that effect concentrations referred to comparable effect levels across different samples, chemicals and mixtures in the same assay and not only relative effect concentrations, such as median activity concentrations, that differ in effect level for different samples even in the same assay.^51^ This approach is vital for the mixture modeling and for analysis of potency and specificity. As outlined in detail in Text S4, we derived absolute 10% inhibitory concentrations (IC_10_) for all for cytotoxicity and inhibitory CRCs.^52^ Effect concentrations were expressed in concentration that triggered 10% of the maximum effect (EC_10_) or induction ratio 1.5 (EC_IR1.5_). We also predicted the baseline cytotoxicity IC_10,baseline_ in order to derive the toxic ratio TR (eq S19), the specificity ratio against experimental cytotoxicity (SR_cytotoxicity_, eq S16) and the specificity ratio against baseline (SR_baseline_, eq S5) as detailed in Text S5.^53^

### Mixture Design

We combined up to 24 chemicals in the concentration ratios of their occurrence in the samples resulting in the fractions *p*_*i*_ of components *i* (eq 4) in the mixtures, where *C*_*i*_ is the concentration of component *i*, and *C*_tot_ the sum of the concentrations (eq 5). The nomenclature and fractions *p*_*i*_ of the mixtures are given in Table S28. The names of the designed mixtures were composed of the sample abbreviation with suffix mix, e.g., WWmix, FFWmix etc.

4 $$p_{i}=\frac{C_{i}}{C_{\mathrm{tot}}},\mathrm{with},\sum p_{i}=1$$

5 $$C_{\mathrm{tot}}=\overset{n}{\underset{i=1}{\sum }}C_{i}$$

Due to the wide range in desired concentrations and limits in solubility, it was not possible to prepare methanol stocks for all chemicals. Therefore, it was decided to use DMSO as solvent for the preparation of the mixtures despite this being problematic for the neurotoxicity assay because DMSO significantly shortened the neurites DMSO. All chemicals with exception of Caffeine were soluble at the desired concentrations in DMSO. Caffeine in WWmix, EFFmix, HBM, S1mix, S2mix, S3mix had to be added directly to the bioassay medium and was therefore provided in dry form to the participating laboratories. In SWmix, S4mix, and S5mix, the caffeine was in such a low concentration that it could be added to the DMSO stock. An overview of the concentrations in all mixture stocks is given in Table S28.

All chemicals were initially dissolved in methanol at concentrations ranging from 0.1 mM to 0.5 M depending on solubility and volume needs for easy pipetting. From these methanolic stocks methanolic mixture stocks were prepared (exomix). We kept the three natural hormones separate in the initial preparation as all mixtures were tested in the androgen receptor (AR) transcriptional activation assay with and without these three hormones (endomix). All other bioassay laboratories only received the entire mix (endomix + exomix in one vial). The mixture stocks were sent to the bioassay laboratories in dried form in vials with inserts. Upon arrival, the bioassay laboratories resolubilized each mixture in 50 μL DMSO and used the 50 μL DMSO stock to prepare 5000 μL of dosing medium, which contained the caffeine that was also weighed in 5 mL vials and sent dry to be dissolved with 4950 μL of medium directly before the experiment. For the AR antagonism assay, the exomix and the endomix were pipetted into individual 300 μL insert vials and tested separately.

### Mixture Evaluation and Bioanalytical Equivalent Concentrations

The basic equation for concentration addition (eq 6)^54^ allows the prediction of the cytotoxicity IC_10_(CA) or effect concentration triggering effect *y*, EC_*y*_(CA), of the mixture at any effect level *y* for a mixture composed of *n* components *i*, present in fractions *p*_*i*_.

6 $${\mathrm{IC}}_{y}\left(\mathrm{CA}\right)=\frac{1}{\overset{n}{\underset{i=1}{\sum }}\frac{p_{i}}{{\mathrm{IC}}_{y,i}}}$$

For cytotoxicity and effects up to a 30% of maximum effect (*y* < 0.3 expressed in fraction of 1), the concentration–response curves (CRCs) from cell bioassays can be approximated by a linear function.^52^ The model for CA (eq 6) can then be simplified for linear concentration–response curves^17^ and specifically for the 10% effect level (*y* = 10%) to eq 7:

$${\mathrm{IC}}_{10}\;\mathrm{or}\;{\mathrm{EC}}_{10}\left(\mathrm{CA}\right)=\frac{1}{\overset{n}{\underset{i=1}{\sum }}\frac{p_{i}\times {\mathrm{slope}}_{i}}{10\%}}=y\cdot \frac{1}{\overset{n}{\underset{i=1}{\sum }}p_{i}\times {\mathrm{slope}}_{i}}$$ 7

The slope of the concentration–response curve of the mixture, slope_mixture_, is then defined by eq 8, and any effect level below 10% can be calculated by eq 9.^17^

8 $${\mathrm{slope}}_{\mathrm{mixture}}=\overset{n}{\underset{i=1}{\sum }}p_{i}\times {\mathrm{slope}}_{i}$$

9 $$\mathrm{Effect}\;y\left(\mathrm{mixture}\right)=\overset{n}{\underset{i=1}{\sum }}p_{i}\times {\mathrm{slope}}_{i}\times C_{\mathrm{tot}}=\left(\overset{n}{\underset{i=1}{\sum }}p_{i}\times {\mathrm{slope}}_{i}\right)C_{\mathrm{tot}}={{\mathrm{slope}}_{\mathrm{mixture}}\times C}_{\mathrm{tot}}$$

A measure of the quality of the mixture prediction is the index on prediction quality (IPQ, eq S25),^55^ which is defined in Text S6.

If additivity has been confirmed for a mixture, the contribution of individual chemicals i to the overall mixture effect can also be easily expressed as the bioanalytical equivalent concentration, BEQ. The BEQ_*i*_ of chemical *i* is the product of the relative effect potency REP*_i_* and the concentration *C*_*i*_ (eq 10).

10 $${\mathrm{BEQ}}_{i}={\mathrm{REP}}_{i}{\times C}_{i}$$

The relative effect potency REP_*i*_ of a chemical (eq 11) and its standard error SE (eq S21 in the Supporting Information) can be calculated as the ratio of the effect or inhibitory concentration of a reference chemical to the effect or inhibitory concentration at effect level *y* of chemical *i*:

11 $${\mathrm{REP}}_{i}=\frac{{\mathrm{EC}}_{y}\left(\mathrm{reference}\right)}{{\mathrm{EC}}_{y}\left(i\right)}$$

The component-based BEQ_chem_ for the mixture (eq 12) and its standard error (eq S22) is estimated as sum of the individual chemicals BEQ_i_:

12 $${\mathrm{BEQ}}_{\mathrm{chem}}=\overset{n}{\underset{i=1}{\sum }}{\mathrm{BEQ}}_{i}=\overset{n}{\underset{i=1}{\sum }}{\mathrm{REP}}_{i}\times C_{i}$$

The measured BEQ_bio,mix_ of the designed mixture (eq 13, standard error eq S23) can then be compared with BEQ_chem_. If CA applies, the experimentally derived BEQ_bio,mix_ should be equal to the component-based BEQ_chem_ that is predicted from the experimental effects of the single chemicals.

13 $${\mathrm{BEQ}}_{\mathrm{bio},\mathrm{mix}}=\frac{{\mathrm{EC}}_{y}\left(\mathrm{reference}\right)}{{\mathrm{EC}}_{y}\left(\mathrm{mix}\right)}=\frac{\mathrm{slope}\left(\mathrm{mix}\right)}{\mathrm{slope}\left(\mathrm{reference}\right)}$$

Modeled BEQ_chem_ and measured BEQ_bio,mix_ can also be compared with experimental BEQ_bio_ of the whole sample extract (eq 14, standard error eq S24).

14 $${\mathrm{BEQ}}_{\mathrm{bio}}=\frac{{\mathrm{EC}}_{y}\left(\mathrm{reference}\right)}{{\mathrm{EC}}_{y}\left(\mathrm{sample}\right)}=\frac{\mathrm{slope}\left(\mathrm{sample}\right)}{\mathrm{slope}\left(\mathrm{reference}\right)}$$

## Results

### Comparability of Extraction Method

Extraction of complex organic mixtures was essential for the selective isolation of complex mixtures of organic chemicals from interfering matrix constituents such as inorganic ions, trace metals, natural organic matter, proteins, and lipids. Beyond enrichment, extraction also served a critical sample cleanup function. Although procedures were harmonized as far as practicable, methodological adjustments were necessary to accommodate matrix-specific characteristics, with extraction protocols optimized individually for each sample type (Figure 1A, Text S1, Figure S1). Water and serum samples were extracted using solid phase extraction (SPE) with a polystyrene/divinylbenzene copolymer as the sorption phase. SPE has shown a mean recovery of 70% (95% CI 67–73%) across >500 chemicals and bioassay responses for water samples in previous studies.^18,56^ Serum, fish and milk extractions were optimized in this study to maximize chemical recovery and minimize matrix effects in bioassays (Text S1, Figure S1). Serum was extracted with SPE.^21^ The recovery of 14 spiked chemicals was 74% (95% CI 56–92%), and no bioassay activity was observed in blanks. Fish and milk samples were extracted with acetonitrile.^57^ To avoid interferences with the analytical method and bioassays, a delipidation step was necessary for milk and fish,^19^ which reduced the detection of some highly hydrophobic chemicals. Despite these necessary methodological differences, the resulting extracts are broadly comparable, capturing both persistent and nonpersistent, neutral and charged organic chemicals—albeit with some bias toward more hydrophilic chemicals over highly hydrophobic chemicals.

### Chemicals Detected across Multiple Matrices

Suspect screening identified 547 chemical features across the 16 extracts analyzed (Figure 1B). Of these, 63 chemicals could be unambiguously identified,^19^ and 45 were semiquantified (Table S2). A subset of 24 chemicals was detected in at least three different sample types, with concentrations ranging from the low nanogram per liter to the low milligram per liter range (Figure 1C, Table S3).

The 24 quantified chemicals closely mirrored the broader patterns observed during suspect screening (Figure 1B), with highest abundance and concentrations detected in WW, followed by EFF and surface water (Figure 1C). Although fewer chemicals were detected in serum extracts, their concentrations were often higher compared to environmental extracts. Human breast milk exhibited chemical profiles more similar to serum than to cow milk. Even fewer chemicals were detected in fish and milk extracts, which might be due to the necessary delipidation step during extraction.

The semiquantified chemicals range from legacy chemicals, such as the DDT metabolite *p*,*p*′-dichlorodiphenyldichloroethylene (DDE), to a variety of contemporary and industrial chemicals. The highest concentrations in wastewater and serum extracts were observed for cosmetic preservatives methyl- and propylparaben, the food-related chemical caffeine, endogenous steroid hormones, and the widely used analgetic acetaminophen. Pesticides were represented by compounds such as the legacy herbicide atrazine and its hydroxy metabolite, as well as current-use pesticides such as the fungicide carbendazim. Industrial chemicals were frequently detected and often present at higher concentrations, including the PVC plasticizer tris­(2-ethylhexyl) trimellitate and the corrosion inhibitor 4-toluenesulfonamide. Chemicals associated with consumer products were also prevalent, such as bisphenols, the pesticide synthesis intermediate and degradation product 4-nitrophenol, and personal care products including the sunscreen agent 2-hydroxy-4-methoxybenzophenone, the insect repellent DEET and the disinfectant triclosan. Additionally, endogenous sex steroid hormones—estrone, estriol, estradiol, progesterone, and testosterone—were detected in the serum extracts.

Hierarchical cluster analysis (SI Text S7) demonstrated that chemical patterns and concentrations were most closely aligned among fish, clean water (BW/DW) and milk, while SW, EFF, WW and serum extracts formed a separate cluster (Figure S2A). DW and BW exhibited lower contamination levels and clearly separated from all other sample types. Fish and cow milk extracts also formed distinct clusters. Except for pesticides, which were predominantly detected in water extracts, no clustering patterns emerged based on chemical use or source.

### Mixture Effects of Extracts in a Battery of Bioassays

The test battery comprised of 22 bioassays incorporated both in vitro bioassays previously applied for chemical mixture testing and others adapted newly for extract-based dosing. Although focusing on human health, many of the bioassays used are also relevant for environmental organisms and are widely applied in environmental monitoring. Among the assays, eight were cell-based reporter gene assays, seven of which were run in 2D high-throughput format, and one was a cardiomyocyte embryoid body assay, derived from human induced pluripotent stem cells (hiPSC), applied for developmental toxicity assessment. Six cell-free assays utilized isolated protein fractions, while seven cellular bioassays relied on the detection of biomarkers in native cells or the use of imaging techniques. The battery additionally included a whole-organism bioassay employing the zebrafish embryo as a proxy of a developing organism. In all cell-based assays, cytotoxicity was assessed in parallel to the specific end points. Only concentrations causing less than 10% cytotoxicity were included for effect evaluation. This strategy was adopted to minimize the risk of false positives resulting from matrix effects or cytotoxicity bursts.^51^ The bioassays underwent rigorous quality control procedures and produced consistent results throughout the study, as demonstrated by appropriate positive and negative controls (SI Text S8, Figure S3, Table S4). All extracts exhibited responses in three or more bioassays (Figure 2; Table S5).

**Figure 2:**
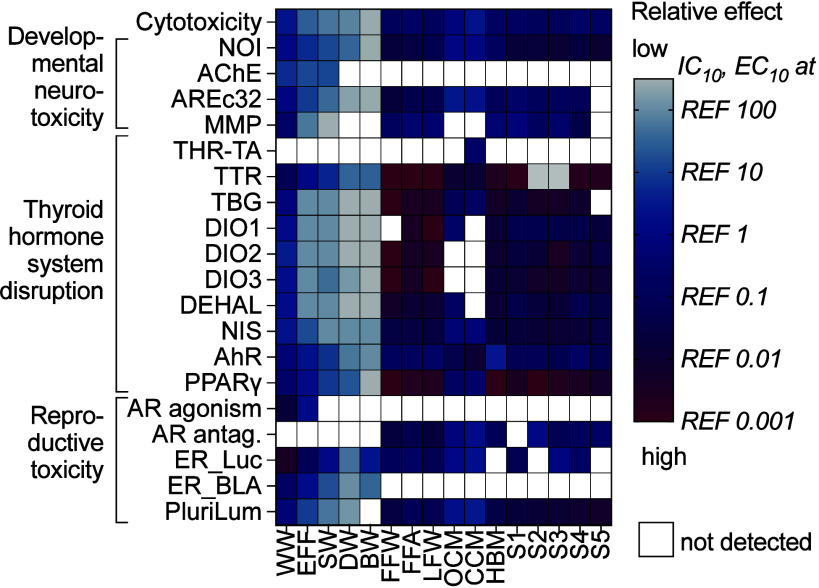
Heatmap of in vitro test battery covering developmental effects triggered by the complex mixtures extracted. Effect concentrations EC_10_ or EC_IR1.5_ in all assays related to developmental neurotoxicity in units of relative extraction factors REF. AChE = acetylcholinesterase, AhR = aryl hydrocarbon receptor, AR = androgen receptor, ARE = oxidative stress response via the keap-Nrf2-ARE pathway, DEHAL = dehalogenase activity 1, DIO = deiodinase activity, ER = estrogen receptor, Luc = luciferase, MMP = mitochondrial toxicity via mitochondrial membrane potential inhibition, NIS = sodium–iodine symporter, NOI = neurite outgrowth inhibition, PPARγ = peroxisome proliferator-activated receptor γ, PluriLum = cardiomyocyte development assay, TBG = thyroxine binding globin, TH = thyroid hormone, THR-TA = thyroid hormone receptor transcriptional activation, TTR = transthyretin. For abbreviations for samples, see legend to Figure 1.

Each extract displayed similar cytotoxicity profiles across all cell lines (SI Text S9, Figure S4), and the red line in Figure 1D illustrates the median inhibitory concentration per extracts causing 10% cytotoxicity (IC_10_, eqs S3 and S5), expressed in units of relative extraction factors (REF) (eq 3). A REF of 1 indicates the concentration present in the original sample (not the extract) relative to that tested in the bioassay, while REF 100 refers to a 100-fold enrichment and REF 0.01 to a 100-fold dilution. Cytotoxicity followed a clear gradient, increasing from DW and BW over SW, EFF to WW. Extracts from milk had similar cytotoxicity as WW. HBM and serum extracts exhibited greater cytotoxicity, requiring five to 10-fold dilutions to reach the 10% cytotoxicity threshold and fish carried the highest burden of cytotoxic chemicals (Figure 1D).

The 10% lethal concentration (LC_10_) in zebrafish embryos (ZFE) corresponded remarkably well with the IC_10_ from the cytotoxicity assays for water, fish and milk but led to mortality in the serum extracts (SI Text S10, Figure S4B, Table S6). As the ZFE results did not provide additional information beyond cytotoxicity, they were not included in subsequent analyses. Behavioral effects and phenotypic changes observed in the ZFE at LC_10_ were subtle, often indistinguishable from those seen in process controls (Figure S5), and could not be reliably assigned to a specific mode-of-action.^58^ Although ZFE are frequently used as a model organism for the evaluation of neurotoxic effects of single chemicals, the ambiguous phenotypic outcomes combined with the consistency between mortality and cytotoxicity suggest that the labor-intensive ZFE assay offers limited added value for broad-spectrum screening of complex environmental and human samples.

Effects in all bioassays typically occurred at lower REFs than those required to induce cytotoxicity (Table S5). Effects observed in process blanks were negligible (Table S5). Given that serum extracts are known to cause background bioassay activity,^14^ the EC values of serum extracts were corrected using values obtained from charcoal-stripped fetal bovine serum (SI Text S11, eq S35). All indicators of developmental neurotoxicity were affected across all extracts and increased proportionally to cytotoxicity and occurred at similar REFs as cytotoxicity, indicating rather low specificity of effects (Figure 1D). End points related specifically to thyroid hormone system disruption exhibited consistently highest activities, in particular TTR, whereas indirectly related pathways (AhR and PPARγ activation) showed the same increasing trend from water to milk, blood and fish but were of lower potency (Figure 1D). For reproductive toxicity, the estrogen receptor (ER) was strongly activated by water extracts with a very high specificity (IC_10_/EC_10_), while in other sample types, ER activation and AR antagonism occurred at similar concentrations. Genotoxicity, assessed on γH2AX induction, was only observed in WW, EFF and FFA extracts, suggesting a clastogenic mode of action but with low potency (SI Text S12).

Detailed bioassay results are presented below for each of the three AOP-networks in separate sections but all results are summarized in a heatmap (Figure 2). Hierarchical clustering of this bioassay heatmap (SI Text S7) revealed that serum, fish, WW and HBM grouped together, while all other water types clustered with cow milk (Figure S2B). Clustering for the bioassays, however, did not yield meaningful groupings, with the three main effect groups distributed across all clusters but a tendency of the thyroid bioassays to cluster closer together, although thyroid-related bioassays tended to cluster more closely.

### Developmental Neurotoxicity

(Developmental) neurotoxicity covers multiple AOPs related to cognitive development and impairment as summarized in Figure S6A, which combines information from multiple AOPs sourced from the AOP-Wiki (aopwiki.org) and recent literature reviews.^59–63^ Common KEs include (a) neuronal cell death, which is downstream of several KEs such as impaired synaptogenesis, degeneration of dopaminergic neurons, and disturbances of neuronal networks and their functions; (b) adaptive stress response to oxidative stress, triggered by reactive oxygen species generated through mitochondrial dysfunction or interference with oxidoreductases, leading to activation of the NRF2-ARE signaling pathway; (c) mitochondrial dysfunction, caused by inhibition of the mitochondrial electron transport chain, uncoupling of oxidative phosphorylation, and inhibition of ATP synthesis; and (d) endocrine disruption, primarily affecting thyroid hormone and retinoic acid pathways.^64^ Thyroid hormone system disruption, given its broader importance in metabolism and development, is addressed separately below.

Blum et al.^65^ developed an in vitro test battery consisting of ten assays covering key neurodevelopmental processes, including proliferation, migration and differentiation of neurospheres, neurite growth, neural network formation and synaptogenesis. These assays are relatively complex, require large sample volume and have not yet been applied to testing of extracts. We applied a simpler screening assay, based on cytotoxicity and neurite outgrowth inhibition (NOI) in differentiated neuroblastoma SH-SY5Y cells,^26^ that has been widely used for water quality testing,^27^ and human biomonitoring.^14^

All water extracts were cytotoxic to neurons (Figure 2 and Figure S6B), but NOI was observed at 3–7-fold lower REFs, indicating moderate neurotoxicity specificity (Table S7). WW extracts showed effects without the need for enrichment, while surface water required a 15-fold enrichment, and BW required a 270-fold enrichment, reflecting the high quality of drinking water. NOI levels in OCM and CCM exposed neurons were comparable to those in WW-exposed neurons. Fish and serum extracts, despite being matrix-corrected using the EC_10_ of charcoal-stripped fetal bovine serum, were highly potent. The neurotoxic effects observed for EFF, SW, and serum extracts aligned well with findings reported in the literature (SI Text S13).

Neurotransmitter signaling can be disturbed by many insecticides acting as inhibitors of acetylcholinesterase (AChE), an enzyme responsible for the breakdown of the neurotransmitter acetylcholine. Consequently, AChE inhibition was determined as an additional end point in differentiated SH-SY5Y cells.^28^ AChE inhibition was observed only in the WW, EFF and SW extracts. No activity was detected in the other extracts, although potential effects may have been masked by cytotoxicity in FFA, FFW, and HBM extracts (Table S8).

Activation of NRF2-ARE-mediated oxidative stress response and mitochondrial dysfunction were evaluated using a multiplexed assay (MitoOxTox), which combines the reporter gene assay AREc32 for ARE activation^29,66^ with a fluorescent dye-based measurement of mitochondrial membrane potential (MMP) inhibition.^30^ Although both end points are mechanistically connected within the AOP framework, they exhibited distinctly different response patterns, consistent with previous observations from water quality monitoring studies.^30^

NOI was the most sensitive neurotoxicity end point with the lowest EC_10_ (Table S7), except for WW extracts, where NRF2-ARE activation (Table S9) and MMP inhibition (Table S10) were more sensitive, and for fish extracts, where NRF2-ARE activation was more sensitive (Figure 2 and Figure S6B). Neurotoxic potency of water extracts followed a decreasing trend from WW to EFF, then SW, and finally DW and BW. As AChE inhibition was only detected in contaminated water samples and showed low specificity, this suggests that future monitoring could focus primarily on NOI as the main neurotoxicity end point. Specificity, expressed as the specificity ratio (SR = IC_10_/EC_10_), was close to 1 for AChE in water extracts, MMP in fish, and ARE and MMP in HBM and serum extracts, indicating observed effects were likely driven by cytotoxicity rather than assay-specific primary mode of action (Figure 2). Although MMP showed high potency for WW extracts with high specificity, its high potency observed in fish and serum extracts was not accompanied by high specificity (Figure S6B). Overall, NOI demonstrated the highest specificity among all end points (Figure 2). Therefore, NOI and MitoOxTox were included in the subsequent mixture study. For more details, see SI Text S13.

### Thyroid Hormone System Disruption

Noyes et al.^60^ proposed an AOP network for thyroid hormone system disruption, identifying 26 MIEs. This highly complex network, summarized in simplified form in Figure S7A, encompasses interference with hormonal feedback and central regulation, thyroid hormone (TH) biosynthesis, excretion, and metabolism, as well as hormone distribution and mechanisms of prereceptor control at the target cell level including transmembrane transport, deiodination, and receptor agonism/antagonism. The hypothalamic-pituitary feedback loop, shown in the upper left corner of Figure S7A, plays a critical role in regulation of TH levels for proper development and homeostasis. Particularly during fetal brain development, decreased or increased levels of circulating TH levels in maternal blood have been associated with reduced IQ and increased risk for neurobehavioral disease in offspring.^67^

Various MIEs can lead to the downregulation of serum concentrations of triiodothyronine (T3) and/or thyroxine (T4). Key targets include modulators of the transthyretin (TTR) and thyroxine-binding globulin (TBG), which prevent the rapid hepatic clearance of free THs, as well as the sodium–iodine symporter (NIS). T4 binds in blood to the distributor proteins such as albumin. These proteins maintain an equilibrium between bound and unbound TH, with the free fraction available for active uptake into target cells via specific transmembrane transporters. After TH biosynthesis, iodinated byproducts are recycled by iodotyrosine deiodinase to prevent loss of iodine. Disruption of this recycling process can be assessed using assays based on recombinant dehalogenase 1 (DEHAL). When reaching their target cells, TH may undergo local and isoenzyme-specific activation and/or inactivation by iodothyronine deiodinases (DIOs) prior to nuclear receptor binding. A decrease in thyroxine (T4) levels may also result from activation of nuclear receptors in the liver. Upon activation, the arylhydrocarbon receptor (AhR), the constitutive androstane receptor (CAR), and the pregnane X receptor (PXR) induce metabolic enzymes, which can conjugate free TH to help excretion. The peroxisome proliferator-activated receptor (PPARγ) is a modulator of KE where metabolic disruption intersects with TH signaling as it can modulate TH signaling via cross-talk with the TH receptor (THR).

We used in vitro bioassays covering 8 of the 26 TH system-specific MIEs inventory by Noyes et al.^60^ and others:^68^ TTR, TBG, THR-TA, DIO1, DIO2, DIO3, DEHAL, and NIS and two broader indicators of cellular stress responses—AhR and PPARγ activation. PXR was excluded due to its upregulation by many chemicals, resulting in low specificity.^69^ Similarly, CAR was excluded due to its low hit rate (only 1%) in ToxCast, suggesting it may not be a reliable indicator of chemical exposure.^69^

THR transactivation was detected only in the CCM extracts and showed lower potency compared to other assays (Figure S7B, Table S11). TTR binding exhibited distinct activity across all extracts and was the most responsive end point (Table S12), followed by TBG (Table S13). The iodothyronine deiodinases (DIO1–3; Tables S14–S16) showed similar or slightly lower potency compared to TBG (Figure 3B). DEHAL (Table S17) and NIS (Table S18) also showed limited responsiveness. A more detailed analysis is provided in SI Text S14.

**Figure 3:**
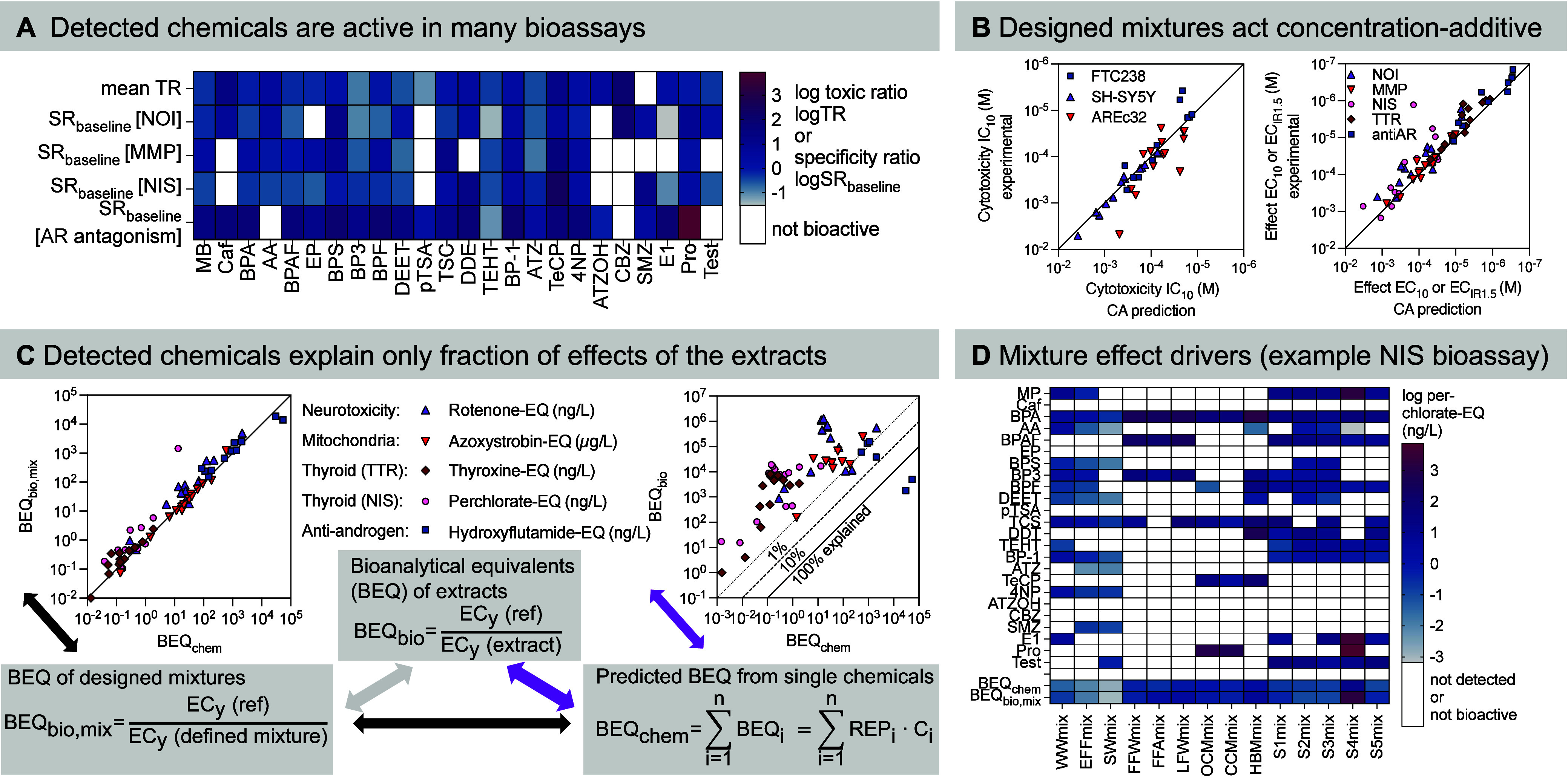
Designed mixture experiments and comparison with whole extracts’ effects. (A) Specificity of detected chemicals: toxicity ratios TR (IC_10,baseline_/IC_10_, eq S19) for mean cytotoxicity of all tested cell lines and specificity ratios SR_baseline_ (IC_10,baseline_/EC_10_, eq S20) for four responsive and cell-based bioassays (NOI, neurite outgrowth inhibition; MMP, mitochondrial toxicity; NIS, sodium-iodide-symporter activity; AR_antagonism, androgen receptor transcriptional activation assay (antagonist mode). Blank fields are missing effect data, i.e. no effect up to the highest tested concentration. Data in Tables S29–S35. (B) Comparison of experimental and predicted mixture cytotoxicity IC_10_ in three cell lines and effects EC_10_ or EC_IR1.5_ in five bioassays. Data in Table S36. (C) Predicted bioanalytical equivalent concentrations BEQ_chem_ (eq 12) of the designed mixtures align well with the experiments of the designed mixtures BEQ_bio,mix_ (eq 13), but the BEQ_chem_ underestimate the measured BEQ_bio_ (eq 14) in the extracts due to the presence of many unknown chemicals or chemicals with unknown potencies. Data in Tables S39–S43. Arrows of the same color link figure with associated equations. (D) Contribution of individual chemicals’ BEQ_i_ to the predicted mixture BEQ_chem_ on the example of the NIS assay. Data in Table S41, this plot for all other bioassays in Figure S14.

The nuclear receptors AhR (Table S19) and PPARγ (Table S20) were activated by most extracts. Potency was generally within the same order of magnitude as observed for other assays, and water extracts showed similar responses for both end points. However, AhR activation was more pronounced in milk extracts, while PPARγ appeared more sensitive to fish and serum extracts. Notably, the specificity ratio for PPARγ activity in fish and serum was often exceedingly high (Figure S7B), which may be attributed to coextracted fatty acids—natural ligands with strong affinity to PPARγ. Due to this potential artifact, combined with the low specificity of AhR, the designed mixture study focused on TTR and NIS bioassays as more reliable indicators for characterizing disturbance of the thyroid hormone system.

### Reproductive and Developmental Toxicity

Male reproductive health is suffering in many countries, where increased rates of testicular nondescent, penile malformations, and hypospadias are observed in newborn boys.^70^ Testicular germ cell cancer incidence is also rising among Caucasian men,^71^ while semen quality declines in Western regions.^72^ These conditions are grouped as the testicular dysgenesis syndrome (TDS), linked to impaired fetal androgen signaling.^73^ The TDS hypothesis suggests that exposure to chemicals capable of disrupting androgen signaling during fetal life contributes to the development of these disorders. Very structurally diverse chemicals can act as antiandrogens and collectively disrupt androgen signaling and male sexual development.^74,75^ Androgen insufficiency in the developing male fetus leads to shortening of anogenital distance (AGD), which is considered a unique, early, and noninvasive biomarker of male reproductive health disorders.^75,76^ Figure S8A illustrates a putative AOP for shortened AGD, highlighting that fetal androgen insufficiency is the primary mode of action leading to this effect and subsequent male reproductive disorders. Androgen insufficiency may result from blocked androgen receptors (ARs) and/or via an inhibited steroidogenesis, with AR antagonism often producing the most marked androgen insufficiency. As markers of potential impacts on reproductive health, we included reporter gene assays for AR antagonism and ER agonism and the PluriLum assay, a 3D hiPSC-based differentiation assay for embryotoxicity. This assay simulates early embryonic development by formation of beating cardiomyocytes, which is relevant for both sexes.^77,78^

The AR-Ecoscreen assay showed activation only in WW and EFF extracts (Table S21). All other extracts exhibited antagonistic effects (Table S21, Figure S8B, SI Text S15). Human cord blood (S4) required a 10-fold dilution to elicit 10% AR antagonism.

The two ER bioassays—ER-Luc (Table S22) and ER-bla (Table S23)—indicated strong estrogenic activity of WW and EFF, while SW exhibited moderate activity; in contrast, DW and BW extracts were clean (Figure S8B). Estrogenic effects correlated with antiandrogenic effects, although the estrogenic responses were 1.1–11 times more sensitive (Figure S9).

Cytotoxicity IC_10_ values were very similar in Plurilum (Table S24) to those observed in the 2D cell cultures, except for the serum extracts, which were 40 to 90 times more potent in the embryoid bodies. However, the specificity of the cardiac marker NKX2.5 inhibition was low (with 1.2 < SR_cytotoxicity_ < 10), and data showed substantial variability. This more labor-intensive assay, which also requires larger extract volumes due to the need for prolonged and repeated dosing, does not provide additional information to justify its use for environmental and human biomonitoring. Therefore, only AR moved onto the mixture study.

### Effects of Designed, Reconstituted Chemical Mixtures in a Subset of Bioassays

Among all detected chemicals by suspect screening, 24 chemicals that were present in at least three matrices were quantified, and chemical mixtures were designed and reconstituted according to their presence in the extracts and tested in a subset of bioassays. Also, single chemicals were tested to allow for prediction of mixture effects by modeling. Due to the wide range in physicochemical properties of the 24 chemicals, spanning 11 orders of magnitude in hydrophobicity and varying from neutral to almost fully anionic, dosing was challenging but successful (Figure S10A, Table S27). We dosed each chemical at a three times higher concentration than its predicted baseline toxicity IC_10,baseline_ (eq S18, Figure S10B, Table S27), representing the minimal toxicity any chemical can trigger, or at its solubility limit if it was lower than the IC_10,baseline_. Using this approach, we were able to experimentally determine cytotoxicity for all chemicals in at least one of the cell lines (Tables S29–S35).

As observed for the extracts, the IC_10_ estimates for cytotoxicity varied only slightly across the different cell lines (Figure S10C). In Figure 3A, the mean toxic ratios TR (eq S19) are shown, while individual IC_10_ and TR values are provided in Tables S29–S31 and S33–S35 and Figure S10D–G. As detailed in SI Text S16, only CBZ, Caf and TeCP were specifically cytotoxic (TR > 10), the other chemicals exhibited only baseline toxicity (Figure 3A). Since not all effect data could be directly matched with cytotoxicity data, Figure 3A displays the SR_baseline_ (eq S20). With few exceptions such as for AR antagonism, most chemicals acted either nonspecifically or only moderately specifically in one or more bioassays, indicating that these common environmental pollutants generally lacked high selectivity for a specific mode of action (Figure 3A).

Most single chemicals affected NOI at EC_10_ values close to their IC_10_ for cytotoxicity, with the exception of BPS, which showed a SR_cytotoxicity_ of 36 (eq S16, Table S29). Thus, neurotoxicity was nonspecific for 96% of the chemicals tested. Activation of the oxidative stress response was minimal and, where observed, was nonspecific (Table S30). Triclosan and TeCP were the only chemicals showing specific inhibition of MMP (Table S31), which is consistent with their known function as uncouplers of oxidative phosphorylation. No specificity analysis could be performed for the cell-free TTR assay (Table S32), but all chemicals were active with IC_10_ ranging from 25 nM to 1.9 mM. In the NIS assay, only BP-1 showed specific activity (Table S33). Twenty out of 24 chemicals exhibited AR antagonistic activity, including bisphenols, phthalates, parabens, UV filters, while the hormones estrone (E1) and progesterone (Pro) acted as both potent agonists and antagonists. As expected, testosterone (Test) was the strongest AR agonist (Table S34).

The cytotoxicity IC_10_ values of the 14 designed mixtures—comprising 3 to 17 components at the concentration ratios detected in the corresponding extracts (Table S28)—spanned 4 orders of magnitude. The bioassay-specific EC_10_ values varied even more widely, covering up to 10 orders of magnitude (Tables S29–S34), with no ARE activation (Table S30) and genotoxicity (Table S35) detected. The experimental results for the five active functional end points (NOI, MMP, TTR, NIS, AR antagonism), including cytotoxicity aligned well with predictions based on concentration addition (CA) (Figure 3B). The index on prediction quality (IPQ, eq S25, Table S36), which equals 0 in the ideal case, was <0.9 for most mixtures. The mean IPQ across all cytotoxicity experiments was 0.25 (95% CI: 0.18 to 0.32) (Figure S11A), and for the effect data from the specific end points, it was 0.31 (95% CI: 0.19 to 0.33) (Figure S11B). IPQ values were independent of the number of active or inactive mixture components (Figure S11C,D). The good agreement between experiments and predictions (Figure 3B) supports the conclusion that concentration addition (CA) is a reliable reference model for predicting the effects of environmentally and human derived chemical mixtures present at low levels.

## Discussion

### Chemicals Detected across Environment-Food-Human Continuum

The broad chemical diversity, detected as real-life mixtures across the environment-food-human continuum, is consistent with previous studies that separately investigated different water types,^79,80^ fish^81^ and human blood.^14^ It is unprecedented for a single study to detect such a wide range of chemicals simultaneously in environmental, dietary, and human samples.

### In Vitro Assays Suitable to Test Diverse Sample Types

This study represents the first comparative assessment of extracts from diverse environmental, food and human samples using a broad test battery of in vitro bioassays. The battery included in vitro bioassays with isolated proteins, 2D cells, one organoid model and an in vivo assay using zebrafish embryos. Notably, the same assays were consistently responsive across all sample types, possibly reflecting commonalities in the pollutant burden across matrices. Co-extracted natural organic matter appeared to interfere with the cell-free bioassays (SI Text S14) but did not affect the performance of the cell-based bioassays under our experimental conditions.^4^ The observed effects in extracts from fish, milk and serum were likely triggered by a combination of endogenous and exogenous compounds. Defining a “natural background” remains challenging, as no truly “clean” biological matrix is available —except for charcoal-stripped fetal bovine serum (FBS), which was used as a clean background for serum blank subtraction (SI Text S11).

Although the embryoid body and the ZFE behavioral assays more closely mimic physiological conditions compared to 2D cellular assays, they did not provide higher information content and exhibited more biological variability. They may be more appropriate for mechanistic studies rather than for effect-based monitoring.

Neurotoxicity, as indicated by NOI, suggests an underlying risk to brain development. The MIE-level effects on the thyroid system were pronounced, though these are more difficult to interpret given the reliance on predominantly cell-free assays. Isolated receptors may be more susceptible to matrix effects of extracts,^82^ an issue typically less pronounced in cellular bioassays.^4^ Bioactivity associated with reproductive effects emerged through AR antagonism and PluriLum responses. The broad battery of bioassays—targeting crucial MIEs and KEs—proved effective in capturing different aspects of toxicity. This panel was streamlined into a smaller screening set for the designed mixture experiments without significant information loss, offering advantages regarding throughput and extract volume requirements.

### Are the Mixture Effects Safe?

Because the bioassays are highly sensitive, the detection of an effect does not necessarily imply that the mixtures pose an adverse risk to ecosystems or human health. To interpret these effects meaningfully, so-called effect-based trigger values (EBT) must be established to differentiate between acceptable and unacceptable mixture effects. EBTs are commonly expressed as bioanalytical equivalent concentrations (BEQ), representing the concentration of a reference chemical that elicits the same potency as the mixture. This approach provides a simple and effective means of communicating mixture effects. Effect concentrations (Table S5) were converted into BEQ_bio_ (eq 14) using reference chemicals listed in Table S37.

EBTs are bioassay-specific and have been previously developed for water quality monitoring,^43^ and, as outlined in SI Text S17, we established interims drinking and surface water quality EBTs for additional bioassays as described previously^83^ and we thereof derived EBTs of wastewater influent and effluent by assuming that effluent is typically 10 times diluted in the receiving stream and that the WWTP can remove 90% of the mixture effect. In a similar approach (SI Text S17), we derived preliminary EBTs for fish and milk (Table S37) based on the Derived No Effect Levels (DNEL), Acceptable Daily Intakes (ADI) or Reference Doses (RfD) of the 24 quantified chemicals (Table S37). Given that only the 24 quantified chemicals were used to derive the EBTs, they should be considered preliminary and indicative rather than definitive thresholds.

All water samples were below their respective EBTs, except for estrogenicity (ER_Luc) in WW and EFF (Figure S12A–E), which has been commonly observed.^84^ In contrast, fish and milk samples slightly exceeded the EBT for NOI, NIS and MMP, but overall remained within an acceptable range (Figure S12F–J). It should be noted that the EBTs for food items were derived from a limited data set, and have not yet been adjusted for the presence of coextracted bioactive endogenous chemicals, and must therefore be considered as preliminary. For human serum, EBTs have not been established yet.

### Mixtures Propagate from Food to Humans

The comparative analysis of complex chemical mixtures extracted from environmental, food, and human samples using a broad panel of in vitro bioassays as indicators of human health suggests a shared pollutant burden and chemical transfer from the environment to food and ultimately to humans. As the samples were pooled from up to ten European countries, conclusions at a single country or individual level are not possible, but the data provide a valuable reference for assessing chemical exposure across the European population.

For a conceptual illustration of how chemical mixtures may relate across the environment–food–human continuum, a simplified model was used to convert food intake levels to BEQ levels at the health-based guidance value (HBGV) for each contaminant, treating the effects observed in food samples (HBM for babies, water, fish and milk for adults) as input data (SI Text S18). Since concentration addition was confirmed for nearly all designed mixtures across all bioassays (Figure 3B), it is justified to translate the effect concentrations of the mixtures into BEQs (Table S37). The BEQ_bio_ of all food items were summed to bioanalytical equivalent doses (BED_food_) by scaling according to food intake (eq S41, Table S38).

Two toxicokinetic model scenarios were considered: (i) complete absorption with immediate distribution and no clearance (eq S42), and (ii) a steady-state model incorporating distribution volume and total clearance, based on estimated mean compound hydrophobicity and half-life (eq S43). Despite of these simplistic assumptions, the predicted blood BEQ aligned reasonably well with measured blood BEQ_bio_ for newborns, with 9 to 83% of BEQ explained by HBM intake. In contrast, only 0.4 to 4.4% of adult blood BEQ_bio_ was explained by modeled intake, reflecting the limited range of food items considered in the model (only water, fish and milk) and the greater diversity of diet in adults (Figure S13, SI Text S18).

Given the uncertainty of the chemical composition of the food and blood extracts, it is futile to attempt a better approximation of the toxicokinetic parameters, let alone the samples are taken independently and do not constitute a true food chain. Nevertheless, this simple exercise demonstrates that the toxic mixture burden of chemicals expressed as BEQ may propagate according to the same general principles as its mixture components.

### Realistic Chemical Mixtures Act in a Concentration-Additive Manner but Exhibit a Large and Hidden Activity That Is Not Yet Explained by Chemical Identification

The 24 quantified pollutants that were identified to be present across many samples represented only a minor fraction of the total chemical activity, illustrating our poor understanding of the complexity of ‘real-life’ chemical mixtures. Hundreds of compounds were identified or detected in the extracts,^19^ and many more remain undetectable by current analytical methods.^7^ The extraction strategy prioritized capturing common chemicals across several different matrices over exhaustively profiling all compounds in a single matrix. As such, the extracts focus primarily on nonvolatile, moderately hydrophobic organic compounds, rather than representing the full “chemical universe.”

We distinguish between BEQ_chem_, predicted from the concentrations of individual quantified chemicals and their REPs, BEQ_bio,mix_, representing the experimental effect of the designed mixture, as well as BEQ_bio_, representing the total effect measured directly in the extracts calculated back to concentrations in the original sample (eq 14). BEQ_chem_ and BEQ_bio,mix_ showed near-perfect agreement confirming CA, whereas the BEQ_bio_ was substantially higher than BEQ_chem_ (Figure 3C).

Only between 10% (for AR-antagonism, Table S43) and less than 1% of the BEQ_bio_ (all other bioassays, Tables S39–S42) could be explained by the quantified chemicals. Among the thousands of chemical features detected in the extracts and the 547 chemicals identified with a high confidence level,^19^ only 24 allowed for reliable concentration estimates, based on available reference standards and suitable calibration procedures. These chemicals therefore accounted for only the tip of the iceberg, with 90 to 99.99% of the observed mixture response remaining unexplained. We can expect that quantifying a larger number of chemicals in the extracts would increase the fraction of the observed effects that can be explained. However, it will not be possible to capture the full bioactivity of complex mixtures, as not all chemicals and transformation products can be identified and quantified. Only in cases where a small number of highly potent and specific chemicals dominate the response can the majority of effects be explained by targeted analysis.^43^

In each bioassay and for each mixture, different chemicals contribute to and dominate the mixture BEQs (Figure 3D for NIS and Figure S14 for all other bioassays, Text S19). While certain chemicals, such as BPA, BPAF, TCS, BPF and BP-3, frequently contribute significantly to the overall mixture effect, they cannot account for it entirely. Given the variability of contributing chemicals across mixtures, it must be acknowledged that an exclusive focus on universal “priority mixtures” (also referred to as “mixtures of concern”) has inherent limitations–especially if the total burden of complex mixtures is overlooked.

Designed mixture at realistic concentration ratios demonstrated concentration addition, regardless of mode-of-action considerations, which justifies the use of bioanalytical equivalent concentrations as a straightforward tool for communicating overall sample toxicity. Even though all detected chemicals were present at individually “safe” levels, the designed mixture experiments and the testing of extracts demonstrated that chemicals can jointly trigger significant effects. This is a regulatory blind spot,^85^ not due to rare synergies, but due to additive effects of many low-dose pollutants. In vitro bioassays, successfully applied in water quality monitoring for many decades and central to NAMs, demonstrate strong applicability in food safety and human biomonitoring, efficiently complementing chemical analysis. The biological effects span species and ecosystems, demonstrating the interconnectedness of environmental, animal, and human health, a core principle of the One Health framework.^86,87^ The responsiveness of in vitro bioassays across diverse matrices reinforces their value as integrative tools to capture mixture toxicity and supports the need for holistic risk assessment strategies that bridge environmental science, toxicology, and public health.

## Supplementary Material





## Data Availability

The concentration–response curves can be accessed at zenodo (https://doi.org/10.5281/zenodo.20402074).

## Notes

Tables S1–S43 with a summary of additional chemical and bioassays information, all raw data of all bioassay experiments and the thereof derived effect concentrations for extracts, single chemicals and defined mixtures; mixture modeling and data for derivation of effect-based trigger values (XLSX)
Texts S1–S19 and Figures S1–S14 describing the optimization of the extraction methods, details of chemical analysis and bioassay measurements, AOP networks and bioassay description, hierarchical clustering of chemical and bioassay data, quality control data for the bioassays, detailed results of samples and blanks for each bioassay, derivation of effect-based trigger values, simplified toxicokinetic model to relate the food intake to blood concentrations, iceberg mixture modeling including heatmaps for all bioassays (PDF)
