# Supplementary material for: In Vitro Bioassay Evidence for Chemical Mixture Propagation from the Environment to Humans
Source: Environ Sci Technol. 2026 Jun 2;60(23):16498–513. doi: 10.1021/acs.est.6c00908 (PMC13290017; doi:10.1021/acs.est.6c00908)
Supplement: Supplementary file 2 [file es6c00908_si_002.pdf]

Supplementary Information

In-Vitro Bioassay Evidence for Chemical Mixture Propagation from the Environment to Humans

Authors: Beate Escher[1,2,3], Martin Scholze[4], Maria Margalef[5], Maria König[1], Maria J. Valente[6], Timo Hamers[5], Kostja Renko[7], Marc Audebert[8,9], Jungeun Lee[1], Laure Khoury[9], Peter Cenijn[5], Yanying Ma[6], Andreas Frederik Treschow[6], Leisa-Maree Toms[9], Christina Rørbye[10], Georg Braun[1], Solène Motteau[11], Jean-Philippe Antignac[11], Gaud Dervilly[11], Marja Lamoree[5], Anne Marie Vinggaard[6]

Corresponding author: beate.escher@ufz.de

Table of content

19 Additional texts (Text S1 to S19–this file)

14 Figures (Figure S1 to S14–this file)

43 Tables (Tables S1 to S43 as xlsx file)

Table of content of the xlsx file:

Table S1 Samples, volume extracted, and maximum relative enrichment factor tested
Table S2 List of chemicals that were semi-quantified
Table S3 Concentrations detected in the samples in mol/L or mol/kg for fish
Table S4 Bioassays and QA/QC reference parameters
Table S5 Summary of bioassay results of the extracts
Table S6 Bioassay results of the extracts in the zebrafish embryo toxicity
Table S7 Bioassay results of the extracts in the neurite outgrowth inhibition assay with SH-SY5Y
Table S8 Bioassay results of the extracts in the acetylcholinesterase inhibition with SH-SY5Y cells
Table S9 Bioassay results of the extracts in the oxidative stress response assays with AREc32
Table S10 Bioassay results of the extracts in the MitoOxTox assay with AREc32
Table S11 Bioassay results of the extracts in the thyroid hormone receptor transactivation assay (THR-TA)
Table S12 Bioassay results of the extracts in the assay for competition with FITC-T4 for transthyretin (TTR) protein binding
Table S13 Bioassay results of the extracts in the assay for competition with FITC-T4 for thyroxine-binding globulin (TBG)
Table S14 Bioassay results of the extracts in the iodothyronine deiodinase type 1
Table S15 Bioassay results of the extracts in the iodothyronine deiodinase type 2
Table S16 Bioassay results of the extracts in the iodothyronine deiodinase type 3
Table S17. Bioassay results of the extracts in the dehalogenase 1 (iodotyrosine deiodinase activity (iodine recycling), DEHAL)
Table S18 Bioassay results of the extracts in the sodium-iodide-symporter (NIS) activity
Table S19 Bioassay results of the extracts in AhR CALUX assay for the activation of the arylhydrocarbon receptor
Table S20 Bioassay results of the extracts in PPARγ-BLA assay for the activation of the peroxisome proliferator activated receptor (PPARγ)
Table S21 Bioassay results of the extracts in the androgen receptor (AR) transcriptional activation assay (agonist and antagonist mode)

Table S22 Bioassay results of the extracts in the ER-Luc estrogen receptor (ER) transcriptional activation assay
Table S23 Bioassay results of the extracts in the ER-BLA estrogen receptor (ER) transcriptional activation assay
Table S24 Bioassay results of the extracts in the PluriLum assays based on 3D embryoid bodies made from hIPSC
Table S25 Bioassay results of the extracts in the assay for induction of phosphorylation of H2AX histone in response to DNA damage (γH2AX) and Phospho-histone 3 (pH3) marker of mitosis and cell cycle arrest in G2/M phase in HepG2 cells
Table S26 Bioassay results of the extracts in the assay for induction of phosphorylation of H2AX histone in response to DNA damage (γH2AX) and phospho-histone 3 (pH3) marker of mitosis and cell cycle arrest in G2/M phase in SH-SY5Y cells
Table S27 Physicochemical properties and baseline toxicity prediction of the chemicals tested individually and in mixtures
Table S28 Mixture stocks concentrations and mixture composition in the bioassays
Table S29 Individual chemicals and defined mixtures in the neurite outgrowth inhibition (NOI) assay with SH-SY5Y
Table S30 Individual chemicals and defined mixtures in the assay for oxidative stress response with AREc32
Table S31 Individual chemicals and defined mixtures in the MitoOxTox assay with AREc32
Table S32 Individual chemicals and defined mixtures in the assay for competition with FITC-T4 for transthyretin (TTR) protein binding
Table S33 Individual chemicals and defined mixtures in the assay for Sodium-Iodide-Symporter (NIS) activity
Table S34 Individual chemicals and defined mixtures in the androgen receptor (AR) transcriptional activation assay
Table S35 Individual chemicals and defined mixtures in the assay for induction of phosphorylation of H2AX histone in response to DNA damage (γH2AX) and

phospho-histone 3 (pH3) marker of mitosis and cell cycle arrest in G2/M phase in SH-SY5Y cells

Table S36 Mixture modelling concentration addition (CA)

Table S37 Reference chemicals for and derivation of bioanalytical equivalent concentrations (BEQ), as well as effect-based trigger values (EBT-BEQ)

Table S38 Simplified toxicokinetic model to relate the food intake to blood concentrations

Table S39 Bioanalytical equivalent concentration model: neurite outgrowth inhibition (NOI)

Table S40 Bioanalytical equivalent concentration model: mitochondrial membrane potential inhibition in AREc32

Table S41 Bioanalytical equivalent concentration model: competition with FITC-T4 for transthyretin (TTR) protein binding

Table S42 Bioanalytical equivalent concentration model: sodium-iodide-symporter (NIS)

Table S43 Bioanalytical equivalent concentration model: androgen receptor (AR) transcriptional activation assay (antagonism)

Supplementary Text

Text S1. Optimization of the extraction methods

Water and serum samples were extracted using solid phase extraction (SPE) with a polystyrene/divinylbenzene copolymer as the sorption phase. SPE has shown mean recovery of 70% (95% CI 67-73%) across >500 chemicals and bioassay responses for water in previous studies.[1, 2] Serum, fish and milk extractions were optimized for the purpose of this study to maximize chemical recovery and minimize matrix effects in bioassays (Figure S1). Serum was extracted with SPE.[3] The recovery of 14 spiked chemicals was 74% (95% CI 56-92%) and bioassays did not show any blank effects. Fish and milk samples were extracted with acetonitrile.[4] To avoid interferences with the analytical method and bioassays, a delipidation step was necessary for milk and fish,[5] which reduced the detection of some highly hydrophobic chemicals.

Water: 2.4 L WWTP influent (WW), 2.4 L effluent (EFF) and 4.8 L surface water (SW) were filtered in each sampling laboratory over glass fiber filters (Whatman Grade GF/F Filter for TCLP Test Use, 0.7 µm, 55 mm). 7.2 L local tap water and 7.2 L locally bought bottled water were extracted with SPE without prior filtration (Table S1). Each sampling laboratory also prepared a SPE blank (BWSPE) with 100 mL HPLC-grade water and a filtration plus SPE blank with 100 mL HPLC-grade water filtered through a glass fiber filter followed by the SPE.

The SPE cartridges were conditioned by adding 2x 5mL of ethyl acetate, followed by 2x 5mL of methanol and lastly 2x 5mL of HPLC-grade water. Then the sampled waters were loaded onto the SPE cartridges with a gentle vacuum. After all water has passed, the cartridges were dried in vacuum for several hours, then wrapped in aluminum foil and shipped to UFZ.

The cartridges were eluted with 2x 5mL of methanol, followed 2x 5mL of ethyl acetate. Before pooling the samples, the individual extracts were tested in the AREc32 assay to confirm that there were no outliers. The individual extracts were combined, aliquoted for the bioassays and chemical analysis, blown down, and shipped to the partner laboratories and reconstituted with methanol to the extraction factor given in Table S1. No recovery correction was made for chemical analysis and bioassays.

The HR-X SPE material for enrichment of the water samples was shown to be compatible with bioassays and chemical analysis.[2] In previous work with a very similar

polystyrene/divinylbenzene copolymer HLB[1] SPE had a mean recovery of 70% (95% CI 67-73%) for 459 chemicals and the quantified chemicals of the present study had a mean recovery of 76% (95% CI 60-91%, n= 15) (Figure S1B).

Serum: Equivolume of sample and formic acid:isopropanol (4:1) were sonicated for 10 min and incubated for 50 min. After addition of the same volume of MilliQ water:isopropanol (4:1), the mixture was sonicated for 10 min, then five times the volume of MilliQ water was added and extracted with HLB (25 mL of original serum per cartridge). The SPE cartridge was eluted with 5 x 5 mL ethyl acetate and 5 x 5 mL 10%MeOH in ethyl acetate.

Further, in order to remove endogenous hormones from the serum samples that may interact with hormone receptor activation bioassays (ER_Luc and AR_antagonism), an additional cleaning step was applied.[6] Briefly, the method used a semi-preparative HPLC with a normal chromatographic phase and two mobile phases: n-hexane and n-hexane:methanol:2-isopropanol (40:45:15 v/v). This method allowed separation of lipophilic xenoestrogens from endogenous hormones and more polar xenoestrogens.

The serum extraction with HLB had a mean recovery of 74% (95% CI 56-92%) for 14 spiked chemicals and the detected chemicals fell into this recovery range (Figure S1B). The bioassays NOI, ARE and AhR were also evaluated with variations of the protocol and two solid materials – HLB (hydrophilic lipophilic balance) and MCX (cation exchange) (Figure S1C). MCX was not suitable as it led to high blank effects and cell debris in the NOI assay (data not shown), reduced activity in the AhR (Figure S1D) and high blank effects in ARE (Figure S1E).

Milk: Comparison of eleven milk extraction methods (Figure S1F) were performed with chemical analysis only. One hundred chemicals (42 pesticides, 13 PAH, 27 phthalates, 12 flame retardants, 4 bisphenols, 1 UV filter and 1 PFAS) were spiked to milk and extracted with miscible (acetonitrile) or non-miscible solvent (acetonitrile/hexane or ethyl acetate/hexane) without and with (QuEChERS[7]) salting out followed by delipidation with Z-sep SPE, solvent (hexane) or Captiva EMR cartridges. For most methods 60-80% of the chemicals were detected after extraction (Figure S1G). Cow milk was finally extracted by liquid-liquid extraction with 800 mL of acetonitrile per 240 mL of milk followed by delipidation with 800 mL hexane.

Fish: 240 g of fish were freeze-dried and extracted with acetonitrile/water (9:1) and according to Pourchet et al.[4] and optimized by Motteau et al.[5] After blow-down of the solvent, the extract was reconstituted in acetonitrile/water (8:2) and lipids were removed by a Captiva EMR cartridge. The fish extraction was optimized in parallel to the milk extraction as described above with results in Figs. S1F and G and the finally chose method in Figure S1H. The mean recovery was highest (60%) and the matrix effect (40%) was lowest for P10, where the samples were extracted and protein precipitated with acetonitrile followed by delipidation with Captiva EMR cartridges.

Fish extracts were also run in bioassays (ARE and AhR) to evaluate the matrix effects of the extracts in the bioassays. Especially co-extracted lipids pose a problem in the bioassays due to reduced sensitivity and precipitation in the wells. Lipid content should therefore be kept below 0.27%.[8] Process blanks did not show any effects but extracts without delipidation precipitated at higher doses or were turbid. Blanks for Z-Pak cleanup led to slight cytotoxicity artifacts but Captiva EMR cleanup did not show any process blank effects. The lipid content of the extract was reduced from 0.33% to 0.09-0.11% by clean-up with Z-Pak or Captiva EMR, which was sufficient to avoid disturbing the bioassay performance. The final chosen method was therefore P10 for fish and HBM (Figure S1H) and P9 for OCM and CCM (Figure S1I) because the milk volume to be processed was too high for Captiva EMR.

Text S2. Chemical analysis

For LC-HRMS analysis, IS included ^{13}C-Atrazine mercapturate, ^{13}C-Atrazine, ^{13}C-Perfluoro-1-octanesulfonate, ^{13}C-Bisphenol S, ^{13}C-BPA glucuronide, ^{13}C-Tetrabromobisphenol A, ^{13}C-Perfloro-n-octanoic acid, ^{13}C-Perfluoro-n-butanoic acid, ^{13}C-Perfluoro-n-decanoic, d_{18}-γ-Hexabromocyclododecane, d_{18}-γ-Hexabromocyclododecane, d_4-Paracetamol, d_9-Caffeine, d_5-2,4-dihydroxybenzophenone, d_8-4,4'-Dimethoxybenzophenone and d_5-2-hydroxy-4-methoxybenzophenone. For GC-HRMS analysis, IS included d_{10}-Phenanthrene, d_{10}-Chlorpyriphos, ^{13}C-Anti-dodecachloropentacyclooctadecadiene, ^{13}C-4,4'-Dichlorodiphenyldichloroethylene and ^{13}C-Atrazine. An internal calibration curve principle was first applied with extracts fortified at 50, 100, 250, 500 and 1000 µg/L for WW in LC and at 10, 50, 100, 250, and 500 ug/L for other extracts (WW in GC and EFF, LFW, CCM,

pooled S1-S5). For mono-2-ethylhexyl phthalate, parabens and hormones in serum samples, an external calibration curve (from 5 to 100 ug/L in solvent) was also applied as the observed signal intensities exceeded the previous internal calibration range. For each analyte, relative response factor (RRF) values were calculated for each fortified concentration level and an averaged value was used for semi-quantification.

$$RRF=\frac{\text{concentration labelled standard} \times \text{area standard}}{\text{concentration standard} \times \text{area labelled standards}} \quad (S1)$$

The detailed settings of the applied LC-HRMS analytical pipeline using a Dionex UltiMate 3000 system (Thermo Fisher Scientific) coupled to a Thermo Q Exactive (Thermo Fisher Scientific, Darmstadt, Germany) are given by Motteau et al.[5] Briefly, an Acquity Premier HSS T3 C18 (2.1 mm × 100 mm, 1.8 µm, Waters) and a Hypersil Gold (2.1 mm × 100 mm, 1.9 µm, Thermo Fisher Scientific) analytical columns were used for fish and cow milk samples. The mobile phase consisted of Acetonitrile and Milli-Q water with 0.3 mM ammonium fluoride in water with the HSS T3 column, and 5 mM ammonium acetate in both solvents with the Hypersil Gold column. Two separate injections were carried out to analyze the samples in both positive and negative ionization modes, using a heated electrospray source (HESI). Data acquisition included first a one-dimensional MS full scan and one second MS/HRMS (top 5 data-dependent acquisition, DDA) fragmentation event. The GC analytical pipeline consisted in a TriPlus RSH autosampler, a Trace 1310 gas chromatography system coupled to an electronic impact ionization source with detection by a Q Exactive (Thermo Fisher Scientific). A DB-5MS (30 m × 0.250 mm, 0.25 µm, Agilent Technologies) column and an electron impact source set at 70 eV were used respectively for chromatographic separation and ionization.

Text S3. Details on the bioassay methods

Zebrafish embryo toxicity

All zebrafish husbandry and experimental procedures were performed in accordance with the German animal protection standards and were approved by the Government of Saxony, Landesdirektion Leipzig, Germany (Aktenzeichen 75-9185.64). Based on the Guidelines on the protection of experimental animals by the Council of Europe, Directive 2010/63/EU, which allows zebrafish embryos to be used up to the moment of independent feeding (approximately 5 days after fertilization). Because embryos used here were no

more than 4 days old, no license is required by Council of Europe (1986), Directive 2010/63/EU or the local authority. ZFE were collected after fertilization and quality of embryo was checked under microscope. Selected zebrafish embryos were added individually to 384-well plates with 40 µL of ISO water. Cytotoxicity IC_{10} from SH-SY5Y (similar to the median cytotoxicity of all cell lines, which were not all available at the time of testing) were used as a REF range finder. Three different REFs were applied (IC_{10}, $IC_{10}/4$, $IC_{10}/8$) with 10 technical replicates each. Methanol stocks of individual extracts were blown down and reconstituted with fresh ISO water to prepare test solution and 40 µL of test medium was added to individual wells. On each 384-well plates, 20 embryos were included as controls. After exposure to the extracts for 96 h, the number of live and dead embryos were counted to calculate the mortality of embryos.

Behavioral experiments were conducted at one fixed concentration per extract corresponding to the lethal concentration for 10% of the embryos LC_{10} and five endpoints were registered: spontaneous tail coiling (STC), photomotor response (PMR),[9] locomotor response (LMR), morphology and heart rate.[10] Zebrafish embryos were added individually to 96-well plates with 180 µL of fresh medium. 20 µL of extract (solvent exchanged to medium) or 20µL of medium for control were added additionally to the individual wells in 16 technical replicates each. At 24 hours post fertilization (hpf), zebrafish embryo movement was recorded with the ZebraBox (Viewpoint Behaviour Technology, Lyon, France) for 1 min without light stimulus for STC measurement. At 30 hpf, the movement was recorded for 1 min with light stimulus from 21 to 25 sec (excitation phase). The pixel changes were quantified for individual embryos based on recorded video using the EmbryoMotion software (https://github.com/sscholz-UFZ/EmbryoMotion). At 96 hpf, a video was recorded again for zebrafish embryo movement for 70 min for LMR measurement with two light phases at 10-20 min and 30-40 min and two dark phases at 20-30 min and 40-60 min. The video was analyzed by KNIME workflow, where pixel changes for individual dark and light phases were considered to quantify motion index. Each zebrafish embryo was imaged with VAST BioImager system (Union Biometrica, Holloston, MA, USA). Prior to imaging, all embryos were anesthetized by adding 20 µl tricaine solution (6 mg/l tricaine, TRIS 26 mM, pH 7.5 ± 0.1) to each well of the microplate. The morphology of each zebrafish embryo was quantified using the FishInspector software (https://codebase.helmholtz.cloud/ufz/tb3-cite/biotox/FishInspector[11]) and heart

rate was derived from video analysis using the FishCardiologist software and further processing with a KNIME workflow.

Neurite outgrowth inhibition assay

SH-SY5Y cells were differentiated with 10 µM all-trans retinoic acid for 72 h. The differentiated cells were seeded in collagen-coated 384-well plates (3,100 cells/well) and incubated for 24 h.

Appropriate volumes of methanol extracts were blown down with nitrogen gas and reconstituted in assay medium. The dosing medium was serially diluted, which resulted in eleven concentrations with two technical replicates. Narciclasine was included in each assay plate as positive control,[12] and unexposed cells served as negative control. After 24 h of exposure, neurite length and cell viability were derived using an IncuCyte S3 live cell imaging system (Essen BioScience, Ann Arbor, Michigan, USA). Neurite length was quantified in the phase-contrast image. For cell viability, Nuclear Green LCS1 and propidium iodide were added to stain total and dead cells, respectively. The fluorescence image further processed using the imaging software in IncuCyte S3. The assay was run in two technical replicates and 11-point serial dilution and repeated in two independent experimental runs.

AChE inhibition assay

After 72 h of differentiation with all-trans retinoic acid, SH-SY5Y cells were seeded in 384-well plates (15,000 cells/well in 10 µL Neurobasal™ medium + 2% B-27 Supplement + 2% GlutaMAX Supplement). The plates were incubated for a further 48 h, then the samples in a dilution series as for the neurite outgrowth inhibition assay were dosed in 30 µL of medium and incubated for 3 h. AChE inhibition was measured with the Ellman's method[13] using a detection mixture containing acetylthiocholine iodide and 5,5´-dithiobis(2-nitrobenzoic acid). The absorbance was detected every minute for 30 mins using an Infinite M1000 plate reader (TECAN, Hombrechtikon, Switzerland). Paraoxon-ethyl was included as positive control; unexposed cells served as negative control. The assay was performed in two independent experiments only for samples active in the first run.

MitoOxTox (ARE and MMP)

The AREc32 cell line expresses luciferase stably under the antioxidant response element-driven NRF-2 and was derived from the MCF7 breast cancer cell.[14] AREc32 was obtained via MTA from Roland Wolf, Cancer Research, UK. The assay was performed as described.[15, 16] 2.65 x 10^3 cells per well were seeded in 30 µL of medium per well (DMEM with Glutamax, 10 % FBS, 100 U/mL penicillin, 100 µg/mL streptomycin) in black, clear bottom poly-D-lysine coated 384-well plates (Corning). The assay was performed similar to AhR-CALUX except for a higher concentration of 1.9 mM D-luciferin in the luciferase substrate buffer. Luminescence was measured to quantify the oxidative stress response as induction ratio of the luminescence signal of the sample divided by the signal of the unexposed cells. To determine cell viability of treated cells and unexposed cells as control, confluency was measured based on phase contrast images acquired using an Incucyte Zoom S3 (Essen BioScience, Ann Arbor, Michigan, USA).[17] In addition, the mitochondrial membrane potential was recorded with the MMP indicator m-MPI (Codex BioSolutions, CB-80600).[18] 1 µg/mL Hoechst 33342 (Invitrogen, H3570) and m-MPI were added per well and fluorescence intensity recorded as described by Lee et al.[18]

Thyroid hormone receptor transactivation assay (THR-TA)

For the thyroid hormone receptor transactivation assay (THR-TA)[19], GH3.TRE-Luc cells were seeded at 80% confluency in 75 cm^2 culture flasks (Corning, Schiphol-Rijk, The Netherlands) in regular growth medium. Twenty-four hours later, growth medium was replaced by PCM for an additional period of 24 h. Cells were then collected by scraping and seeded into 96-well plates at a fixed density of cells per well and incubated for 24 h in the presence or absence of T3, with or without the sample/blank extracts in 0.5% DMSO. A full T3 standard curve was included on each exposure plate.

After the 24h exposure period, cell viability was determined as cellular metabolic activity by measuring the reduction of resazurin to fluorescent resorufin as previously described.[20, 21] After four hours of incubation with resazurin in the dark (37ºC; 95% air/5% CO_2), fluorescence was measured at λ_{ex} = 530 nm and λ_{em} = 590 nm (Millipore Cytofluor 2350 Fluorescence Measurement System, Burlington, MA, USA). Then medium was removed and luciferase activity was measured in lysed cells in a microplate luminometer

with two injectors (Thermo LabSystems Luminoskan Ascent), as previously described by Murk et al.[22]

Transthyretin (TTR) protein binding

100 µL of a TRIS-buffer solution was added to all wells of a 96 wells plate, after which 2 µL was added of the extract dilution series, negative control (DMSO), and a calibration series of reference compound T4 (all n=2). Subsequently, FITC-T4 (110 nM final concentration) and TTR solution (30 nM final concentration) were added, reaching a final volume of 200 µL. Plates were incubated at room temperature for 15 minutes in the dark on a plate shaker (Titramax 1000, Heidolph, Schwabach, Germany) at 600 rpm. Fluorescence (expressed as arbitrary fluorescence units (AFUs)) was measured after each time a reagent was added in the 96 wells plate at λ_{ex} = 487 and λ_{em} = 528 using a Citation 5 image reader (BioTek, Winooski, VT, USA) operated with the Gen 5 microplate reader and imager software (version 3.10; BioTek).

Thyroxine-binding globulin (TBG)

The TBG binding was measured in black nonbinding 96 wells plates (Greiner) containing 10 nM thyroxin binding globulin (Bio-Rad), 5 nM FITC-T4, and sample test dilutions (0.5% DMSO) in a final volume of 100 ul Tris-HCl buffer (0.1 M Tris, 0.1 M NaCl, 1 mM EDTA) pH 8.0 according to Shen et al.[23] The fluorescence anisotropy was measured on a Pherastar plate reader (BMG Labtech, Ortenberg, Germany) with at λ_{ex} = 485 nM (polarized) and λ_{em} = 520 nM (parallel and perpendicular). Wells containing only FITC-T4 were used to set the gain of the instrument. Polarized and unpolarized background fluorescence of the samples were checked and only sample dilutions with <20% autofluorescence were used to calculate the anisotropy.

Iodothyronine Deiodinase types 1 to 3

Enzymes were extracted from HEK293 cells, recombinantly expressing a respective enzyme variant.[24] The enzymes were incubated with their preferred, iodinated substrates (rT3 for DIO1, T4 for DIO2 and T3 for DIO3) in the presence of (solvent) control, positive control (propythiouracil (PTU) or xanthohumol (XN)) or test extract at various REFs. Released iodide is representing the enzymatic activity and was separated from the intact

TH substrates via ion exchanger columns and quantified by an iodine-sensitive redox reaction, the Sandell-Kolthoff-reaction, by photometry.[25]

Dried extracts were solved in DMSO, 50-fold concentrated in relation to the highest REF within the run. 25 µL of extract was diluted in 100 µL ddH2O. From here, a 1:3 serial dilution was prepared using ddH_2O/20 % DMSO (8 REFs in total). From this dilution series, 10µL was transferred into a 96well qPCR plate per isoenzyme.

40 µL of diluted enzyme was added to each well and the reaction was started with another 50 µL of the substrate master mix, resulting in final concentrations of 0.1 M KPO_4 (pH 6.8 for DIO1&2, pH 8 for DIO3), 1mM EDTA, 40 mM DTT and 10 µM substrate (rT3 for DIO1, T4 for DIO2, T3 for DIO3).[24] After incubation (2h for DIO1 and 3, 4h for DIO2) at 37°C under constant shaking (600rpm), 75µL of reaction mixture was transferred to a prepared column pack (600 µL DOWEX 50wx2, Thermo Fischer Scientific, Darmstadt, Germany). Subsequently, 100µL acetic acid (10 %) was added to each column and the released iodide was eluted by centrifugation (1min, 200g) into a microtiter plate. Following a further dilution step (12 µL eluate + 38 µL acetic acid), the microtiter plate (TPP, Trasadingen, Switzerland) was transferred to a dedicated fume hood to initiate the Sandell-Kolthoff-reaction. After addition of 50 µL Ce-Solution (25 mM $(NH_4)_4Ce(SO_4)_4$ and 0.5 M H_2SO_4) and 50 µL As-solution (25 mM $NaAsO_2$, 0.5 M H_2SO_4, 0.2 M NaCl), OD at 416nm was recorded at 0min and 20min, to follow the iodide-depending destaining kinetics.

The grade of destaining (dOD_{416nm} at 20min) correlates with the amount of iodide, released from the substrate by the respective enzyme. Solvent control (2 % DMSO) defines 100 % activity, while high concentrations of known inhibitors (propylthiouracil (PTU) for DIO1, xanthohumol for DIO2 and DIO3) are used to define enzyme-independent background (0%) activity.

Dehalogenase (Iodotyrosine Deiodinase)

The protocol for the detection of DEHAL1 inhibition roughly follows the DIO-protocol, using an own source of enzyme (expression in FTC238 cell line) and a completely different composition of the reaction mixture, containing monoiodotyrosine (MIT) as substrate for deiodination. While the overall procedure (preparation, incubation, iodine separation and Sandell-Kolthoff-reaction) is very similar to the DIO protocol, the reaction mixture is optimized for DEHAL1 containing 0.1M K_3PO_4 (pH7), 0.2 M KCl, 0.01 M b-

mercaptoethanol, 800 µM NADPH, 30 µM FAD, 10µM MIT.[26] Extracts sample handling for testing followed the same procedure as described above.

Sodium-Iodide-Symporter (NIS)

The Sodium-Iodide-Symporter (NIS) assay utilizes a FTC238-derived cell line, stably expressing a fusion protein, harboring the functional human sodium-iodide-symporter and the reporter protein firefly luciferase (FLuc) as described before with HEK293 cells.[27] A clone with high transporter activity was chosen and used for all experiments. Cells are exposed to iodide in the supernatant in the presence of (solvent) control, positive control ($NaClO_4$) or test extract in a range of REFs. After a certain period of incubation, the supernatant is removed, the cells are lysed and the lysate is tested for intracellular iodide, reflecting the activity of the NIS transporter. A minute fraction of the lysate was further used to determine cell viability after exposure using intracellular ATP concentration as readout (CelltiterGlo, Promega, Madison, WI, USA). Extract sample preparation followed the same procedure as described for the deiodinases. For the chemicals and chemical mixtures, a 100-fold stock solution was prepared by adding DMSO. For each run, 6 µl stock was mixed with 294 µl HBSS (with 0.1 % BSA) and a 1:4 serial dilution was prepared using HBSS/2 % DMSO.

Two days before the uptake experiment, 50.000 cells were seeded in a 96-well microtiter plate (TPP) in 200µL (IMDM, 10% FBS, pen/strep). On the day of the experiment, plates were removed from the CO_2 incubator and washed with PBS. 40µL of Hank's buffered salt solution (HBSS) with 0.1% BSA, 10µL of tested extract, (solvent) controls or positive control (1mM $NaClO_4$) was added to each well and uptake was started by adding 50µL HBSS (0.1%BSA) containing sodium iodide (40µM).

Plates were incubated for 30min at 37°C under non-sterile conditions. Uptake was stopped by removing the supernatant and rapid washing up to four times with cold PBS and ddH_2O (60 µL of TritonX in ddH_2O (0.1 %) was added to the cells and plates were shaken for 5min at 400rpm. 10 µL of lysate was transferred to a white microtiter plate, 20 µL Celltiter Glo 2.0 reagent (Promega) was added, and plates were immediately measured for luminometric signals representing ATP content/viability. The remaining lysate was transferred into the dedicated fume hood and relative iodide uptake was quantified by Sandell-Kolthoff-reaction as described above. For the extracts, three

independent experiments were conducted with a single replicate per concentration. For the testing of chemicals and chemical mixtures, three independent experiments were conducted, each with three technical replicates/concentration.

Androgen Receptor (AR) Transcriptional Activation Assay

The AR-EcoScreen™ cell model (Test No. 458 of the OECD Guidelines for the Testing of Chemicals[28]) is derived from a Chinese hamster ovary cell line (CHO-K1), with three stably inserted constructs: (i) the human androgen receptor (AR) expression construct, (ii) a firefly luciferase reporter construct, and (iii) a renilla luciferase reporter construct for cell viability measurement.

This cell model was used to assess the AR agonistic potential of water samples, and the AR antagonistic potential of fish, cow milk, human breast milk, human adult and cord blood serum samples. Data were normalized to a negative control (untreated cells) and a reference compound – methyltrienolone (R1881) for AR agonism, and 2-hydroxyflutamide (OHF) for AR antagonism.

Cells were seeded in 96-well plates at a density of 9 x 10^3 cells/well (DMEM/F12, 100 U/mL penicillin, 100 µg/mL streptomycin, 5% charcoal-treated FBS) in white flat-bottom plates and allowed to attach for 24 h. Cells were then exposed to extracts and reference compounds. For the assessment of antiandrogenic activity, cells were exposed to extracts, single chemicals or mixtures and OHF as reference chemical in the presence of a fixed concentration of an AR agonist (0.1 nM R1881). After 24 h of exposure, both enzyme activities (firefly for AR activity measurement and renilla for cytotoxicity evaluation) were measured in each plate, using the Dual-Glo® Luciferase Assay System (Promega), according to the instructions of the manufacturer.

PPARγ-GeneBLAzer

The PPARγ-BLA assay using GeneBLAzer PPARγ-UAS-bla 293H cells (Thermo Fisher) was performed solvent-free according to König et al.[29] and Neale et al.[16] with 6.5 x 10^3 cells per well in 30 µL 98% Opti-MEM supplemented with 2% charcoal-stripped FBS 100 U/mL penicillin and 100 µg/mL streptomycin. Solvent-free means that a defined amount of methanolic extract was transferred to a glass vial, the methanol evaporated in a gentle nitrogen stream or in a vacuum evaporator, and then redissolved in the appropriate

bioassay medium. After 24h of incubation at 37°C at 5% CO_2, the extracts that were solvent exchanges from methanol to medium were serially diluted and 10 μL were dosed to the cell plate, resulting in 40 μL of bioassay medium. Rosiglitazone was used as reference compound. Cytotoxicity was assessed by cell growth quantified by confluency measured with phase-contrast imaging.

The formed β-lactamase was quantified with the GeneBLAzer® FRET Assay from Thermo Fischer Scientific, using a specific fluorescent substrate for the β-lactamase, whose implementation depends on the Forster resonance energy transfer (FRET). The substrate readily enters the cell, where endogenous esterases rapidly convert it into a fluorescent precursor that can be selectively quantified using Forster resonance energy transfer (FRET) fluorescence at (blue) and that is transformed into an enzyme product by β-lactamase with green fluorescence. The blue-to-green (B/G) ratio is a measure of the effect that was normalized to the maximum effect of the positive control rosiglitazone as 100% and the B/G ratio of the unexposed cells as the minimum (0%).

AhR CALUX

The rat hepatoma cell line H4L7.5c2 stably expressing the luciferase reporter gene plasmid pGudLuc7.5 containing a total of 20 XREs was used in the AhR CALUX assay.[16, 30] The cell line was obtained via MTA from Michael Denison, UC Davis, USA. 3.5 x 10^3 cells per well were seeded in 30 μL of medium per well (DMEM with Glutamax, 10 % FBS, 100 U/mL penicillin, 100 μg/mL streptomycin, 0.4 mg/mL geneticin) in black, clear bottom poly-D-lysine coated 384-well plates (Corning). Plates were incubated for 24 h. Cells were treated with 10 μL/well of the dosing medium containing the samples (solvent-free), blanks or controls and incubated for 24 h.

Luminescence was measured and the AhR activity potential of the samples evaluated against the reference TCDD. To measure luciferase activity, cells were washed twice with PBS and subsequently 20 μL of lysis buffer was added (25 mM Tris, 1 % Triton-X 100, 2 nM EDTA, 2 mM DTT, 10 % glycerol). After a 10 min incubation period at RT 20 μL of luciferase substrate buffer (20 mM Tricine, 2.67 mM MgSO4, 33.3 mM DTT, 0.1 mM EDTA, 0.261 mM coenzyme A, 0.53 mM ATP, 0.47 mM D-luciferin was added to each well and luminescence was read. Cell viability was determined following the same principle as for the ERα-GeneBLAzer assay.[17]

Estrogen Receptor (ER) Transcriptional Activation Assay

The VM7Luc4E2 ER transactivation assay model (Test No. 455 of the OECD Guidelines for the Testing of Chemicals[31]) is derived from the human breast adenocarcinoma cell line MCF7 and endogenously expresses both human estrogen receptor (ER) forms, ERα and ERβ, and that have been stably transfected with a firefly luc reporter construct to detect substances with in vitro (anti)estrogenic activity.

This cell model was used to assess the estrogenic activity of all pooled samples and blanks. Data were normalized to a negative control (untreated cells) and 17β-Estradiol (E2) as a reference compound for ER agonism.

Cells were seeded in 96-well plates at a density of 40 x 10^3 cells/well (DMEM/F12, 100 U/mL penicillin, 100 µg/mL streptomycin, 5% charcoal-treated FBS, 2% L-glutamine, 110 mg/mL sodium pyruvate) in white flat-bottom plates and allowed to attach for 24 h. Cells were then exposed to each pooled sample/blank/E2. After 24 h of exposure, estrogenic activity was measured in each plate, using the Steady-Glo® Luciferase Assay System (Promega), according to the instructions of the manufacturer. ATP measurement was used as an indirect measure of loss of cell viability, using the CellTiter-Glo® Luminescent Cell Viability Assay (Promega).

Estrogen Receptor (ER) Transcriptional Activation Assay GeneBLAzer

GeneBLAzer ERα-UAS-bla GripTite cells (Thermo Fisher) are quantifying the activation of ERα using the reporter gene BLA encoding β -lactamase as described above for GeneBLAzer PPARγ-UAS-bla 293H, using estradiol (E2) as reference compound.[16]

5 x 10^3 cells per well were seeded in 30 µL of medium per well (Opti-MEM, 2 % csFBS, 100 U/mL penicillin, 100 µg/mL streptomycin) in black, clear bottom poly-D-lysine coated 384-well plates (Corning). Cells were treated with 10 µL/well of the dosing medium containing the samples (solvent-free), blanks and the reference estradiol (E2) and incubated for 24 h. For the detection of the expression of β-lactamase the ToxBLAzer detection reagent was prepared according to the instructions of the manufacturer and 8µL of the reagent were added per well. Fluorescence was read with excitation at 409 nm and emission at 460 nm (blue) and 530 nm (green) immediately after addition of the reagent

(t = 0h) and after 2h of incubation at room temperature in the dark. Cytotoxicity was derived from image-based confluency measurements.[17]

PluriLum assay

The PluriLum assay is based on 3D embryoid bodies made from hIPSC that can be differentiated to beating cardiomyocytes. We have introduced a luciferase reporter gene into the locus of NKX2.5 of this hiPSC line enabling us to measure luminescence intensities instead of counting beating cardiomyocytes, which is less labor-intensive.[32]

The assay was performed as detailed by Treschow et al.[33] Briefly, HiPSC were harvested as single cells and seeded at a density of 5 × 10^4cells/ml into 96-well conical bottom plates in mTeSR-ROCK medium. Plates were then centrifuged at 500g for 5 min at RT and incubated overnight to allow the formation of spheroids. Cultures were kept for 7 days, medium was changed on days 0, 1, 2, 3 and 6, and the appropriate factors were added at each appropriate day to promote differentiation of cells into cardiomyocytes. On days 1, 2, 3 and 6, samples/blanks/ATRA were added to the exposure media. On day 7, the embryoid bodies were rinsed with DPBS and fully dissociated using a papain solution. Cell suspensions were then split in two for assessment of NKX2.5 luciferase luminescence using the Nano-Glo® Luciferase Assay System (Promega) and measurement of cytotoxicity, using the CellTiter-Glo® Luminescent Cell Viability Assay (Promega). Data were normalized to a negative control (untreated cells) and all-trans retinoic acid (ATRA) as a reference compound for differentiation inhibition.

γH2AX and pH3 assay for genotoxicity

The γH2AX/pH3 assay was performed with the In-Cell Western (ICW) technique[34-38] on the neuronal cell line SH-SY5Y and the metabolically competent cell line HepG2. Briefly, cells were distributed and cultured in a 96-well cell culture plate (6 x10^4 cells/well and 3.2 x10^4 cells/well respectively) in a culture medium adapted (αMEM medium for HepG2 and DMEM medium for SH-SY5Y, each supplemented with 10 % FBS, 100 U mL^{-1}penicillin and 100 μg mL^{-1} streptomycin). Sixteen hours later, the cells were treated with the different samples in serum free medium. After 24 h of treatment, the cells were washed in PBS and fixed with 4% paraformaldehyde in PBS at room temperature (RT), then washed with PBS. Cells were permeabilized with 0.2% Triton X-100 in PBS then washed

with PST buffer (PBS, 2% fetal calf serum and 0.2% Triton X-100). Cells were blocked with MAXblock Blocking medium during 1 h and washed with PST buffer, followed by 2 h incubation with both rabbit monoclonal anti-γH2AX (Clone 20E3, Cell Signaling, Danvers, MA, USA) and mouse monoclonal anti-pH3 (Clone CMA312, Millipore) in PST buffer.
After three washes with PST, secondary detection was carried out using three different infrared fluorescent dyes in PST buffer: a goat anti-rabbit antibody (CF770, Biotium, Fremont, CA, USA), a goat anti-mouse antibody (CF680, Biotium) and SYBR™ Gold (Invitrogen, now Thermo Fisher) for DNA labeling. After 1 h of incubation and three washes with PST, the three biomarkers γH2AX, pH3 and DNA dye were simultaneously visualized using the Sapphire Biomolecular Imager (Azure Biosystems, Dublin, CA; USA). Raw absorbance data were corrected for background.
To determine cytotoxicity, the DNA content (related to the number of cells) recorded in the different treated cells was compared to the DNA content in cells treated with blanks. For the determination of genotoxicity, relative fluorescent units for γH2AX (or for pH3) per cell (as determined by γH2AX (or pH3) divided by DNA content) obtained with the sample of interest were divided by the respective controls (blanks only) to determine the change in the level of phosphorylation of histone to the cells treated with the sample compared with the level of phosphorylation of histone to the cells treated with blanks.

Text S4. Concentration-response modeling

Cytotoxicity is typically observed between 100% cell viability (0% cytotoxicity) and 0% cell viability (100% cytotoxicity), and the concentration-response pattern can be described by a log-logistic concentration-response model (eq. S2), where C stands for concentration and IC_{50} for inhibitory concentration causing 50% cytotoxicity. It is possible to derive thereof the IC_{10}, the inhibitory concentration causing 10% cytotoxicity with eq. S3.

$$\text{Cytotoxicity (\%)} = \frac{100\%}{1+10^{\text{slope}(\log IC_{50} - \log C)}} \quad \text{(S2)}$$

$$\log IC_{10} = \log IC_{50} - \frac{1}{\text{slope}} \cdot \log\left(\frac{\text{max}-\text{min}}{\text{y}-\text{min}} - 100\%\right) \quad \text{(S3)}$$

Analogously, for the concentration-cell viability relationship, the IC_{10} of cytotoxicity corresponds to the IC_{90} of cell viability and the corresponding equations can be used.
For most cell lines cytotoxicity was assessed with different viability/activity dyes. Confluency was used to quantify the growth of cells and cytotoxicity in the bioassays NOI,

AChE, ER_Luc, AREc32, AhR, and PPARγ. The imaging-based measure of confluency is not reliable if cell growth is not only stopped but cells are killed and then confluency would be lower after 24h than directly after dosing. However, the concentration-response relationship is typically linear up to 30% of cytotoxicity,[39] and in this range confluency is a good measure of reduced cell proliferation and cytotoxicity.[17] Then the CRC simplifies to eq. S4 and the IC_{10} and its standard error SE can be directly derived with eq. S5 and S6.[39]

Cytotoxicity (%) = slope x C or cell viability (%) = 100% -slope x C (S4)

$$IC_{10}=\frac{10}{slope} \quad (S5)$$

$$SE\ (IC_{10})\approx\frac{10}{slope^2}\cdot SE(slope) \quad (S6)$$

The 10% effect level for cytotoxicity served as cut-off for activation data because at cytotoxic concentration, activation of specific endpoints is often observed but this is an artifact of the cytotoxicity burst.[40-42] It has been observed that many stress responses are activated at concentrations close to cell death but that these activations are not a specific effect but just a general stress response. This has also been observed in omics studies, which are always dosed at non-toxic concentrations to avoid artifacts by non-specific activation.

An exception were the genotoxicity assays (γH2AX_Hep, γH2AX_Neu, pH3_Hep, pH3_Neu), where the IC_{50} was used as a concentration cut-off because it has been demonstrated that to limit the cytotoxicity to 50% permit to differentiate true genotoxicity from false-positive genotoxic results due to apoptosis.[36, 43] Indeed, the results of many studies performed using flow cytometry or ICW techniques with non-genotoxic chemicals that induce apoptosis identified no false-positive compounds with the γH2AX biomarker at sub-toxic concentrations. Moreover, as H2AX and H3 phosphorylation are indicators of early DNA damage, damage can be assessed at an early time point, thereby avoiding false-positive results due to apoptosis induction.[44] Lethal concentrations for 10% of the ZFE, IC_{10}, were derived from log-logistic CRCs. When insufficient datapoints were available, the hill slope was fixed to 1.

If the effects range from 0 to 100%, eqs. 10 and 11 can be used analogously as for cytotoxicity to derive the effect concentration triggering 50% of maximum effect, EC_{50}, and 10%, EC_{10}. If the concentration-response data suggest a minimum (min) and a

maximum (max) level that are different from 0 and 100%, then a four-parameter log-logistic model was used to fit the $EC_{median\ effect}$. The EC_{50} for an absolute 50% effect level can be calculated with eq. 8 and the EC_{10} can be calculated with eq. 9.

$$\text{Effect}(\%)=\min+\frac{\max-\min}{1+10^{\text{slope}(\log EC_{\text{median effect}}-\log C)}} \quad (S7)$$

$$\log EC_{50}=\log EC_{\text{median effect}}-\frac{1}{\text{slope}}\cdot\log\left(\frac{\max-\min}{50\%-\min}-100\%\right) \quad (S8)$$

$$\log EC_{10}=\log EC_{\text{median effect}}-\frac{1}{\text{slope}}\cdot\log\left(\frac{\max-\min}{10\%-\min}-100\%\right) \quad (S9)$$

Again, at effect levels below 30% the log-logistic equation can be simplified to a linear regression through the intercept zero (eq. S10), which facilitates the derivation of an EC_{10} and its SE with eq. S11-S12.

$$\text{Effect}(\%) = \text{slope} \times C \quad (S10)$$

$$EC_{10}=\frac{10}{\text{slope}} \quad (S11)$$

$$SE(EC_{10})\approx\frac{10}{\text{slope}^2}\cdot SE(\text{slope}) \quad (S12)$$

Genotoxicity and oxidative stress response have no 100% effect, hence the induction ratio IR, i.e., the signal of the sample over the signal of the control was used as effect measure. At effect levels below IR 4, these concentration-response curves are typically linear and can be fitted with a linear regression through the intercept of IR 1. In addition, only concentrations that are below the IC_{10} of cytotoxicity (or IC_{50} for the genotoxicity assays γH2AX_Hep, γH2AX_Neu, pH3_Hep, pH3_Neu), were used for concentration-induction curves. The effect concentration is the $EC_{IR1.5}$, i.e., the concentration that causes an increase in IR by 50% over the control, which corresponds to IR = 1.5. IR was used as effect endpoint for AREc32, γH2AX_Hep, γH2AX_Neu, pH3_Hep, pH3_Neu.

$$IR = \text{slope} \times C + 1 \quad (S13)$$

$$EC_{IR1.5}=\frac{0.5}{\text{slope}} \quad (S14)$$

$$SE(EC_{IR1.5})\approx\frac{0.5}{\text{slope}^2}\cdot SE(\text{slope}) \quad (S15)$$

Text S5. Specificity analysis

The specificity ratio $SR_{cytotoxicity}$ is the ratio between cytotoxicity (IC_{10}) and effect concentration (EC_{10} or $EC_{IR1.5}$).[42] The specificity ratio can be deduced for the complex mixtures in the sample extracts, for individual chemicals and for the designed mixtures.

$$SR_{cytotoxicity}=\frac{IC_{10}}{EC_{10}} \quad \text{or} \quad SR_{cytotoxicity}=\frac{IC_{10}}{EC_{IR1.5}} \tag{S16}$$

For single chemicals, one typically considers $SR_{cytotoxicity} \leq 1$ as not specific, $1 \leq SR_{cytotoxicity} < 10$ as moderately specific, $10 \leq SR_{cytotoxicity} < 100$ as specific and $100 \leq SR_{cytotoxicity}$ as highly specific.[42]

Each substance has a unique dose-response profile across the bioassays, and single chemicals might have different or no effect in the specific endpoints. However, cytotoxicity limits as to where dosing is meaningful. If only cytotoxicity is observed, the chemical is not inducing any specific effect. If neither cytotoxicity nor specific effects are detected up to the highest tested concentration, it is possible that dosing was just too low. Therefore, it is important to try to dose to concentrations that reach cytotoxicity to assure that specific effects are not overlooked. As solubility hampers dosing of high concentration, we oriented ourselves on predicted baseline toxicity, which is the minimal, non-specific toxicity any chemical exhibits, to define appropriate maximum dosing concentrations.

Baseline toxicity leading to 10% cytotoxicity occurs at constant critical membrane concentration $IC_{10,membrane}$ and the nominal concentration for this membrane concentration $IC_{10,baseline}$ can be calculated from a mass-balance model (eq. S17) that accounts for proteins and lipid in cells and medium.[45]

$$IC_{10,nom,baseline}(M)=\frac{IC_{10,membrane}}{D_{lip/w}} \times \left(1+D_{BSA/w^{\circ}} \times {}^{\circ}VF_{protein,medium}+D_{lip/w}{}^{\circ} \times {}^{\circ}VF_{lipid,medium}+D_{cell/w} \times \frac{V_{cell}}{V_{bioassay}}\right) \tag{S17}$$

The ionization-corrected distribution ratios between bovine serum albumin (BSA) and water $D_{BSA/w}$ served as proxy for protein binding in the medium and ionization-corrected distribution ratios between biomembranes (liposomes) and water $D_{lip/w}$ served as proxy for lipid binding in the medium and were predicted.[46, 47] or taken from literature.[46, 48-50]

Unless serum-free medium is used, the volume fraction of proteins and lipids in the cells are negligible,[51] and eq.S17 simplifies to eq.S18. The critical concentration $IC_{10,membrane}$ previously was estimated to be 26 mM[51] for several human cell lines, and the volume fractions of proteins in medium $VF_{protein,\ medium}$ depend on the medium and was measured as 0.238% for NOI, 0.302% for AREc32 and AhR, and 0.094% for ERα and PPARγ.[52] The volume fractions of proteins in medium $VF_{lipid,\ medium}$ were 0.0004% for NOI, 0.007% for AREc32 and AhR, and 0.001% for ERα and PPARγ.[52] The $VF_{protein,\ medium}$ and $VF_{lipid,}$

medium of the AR assays was extrapolated assuming 5% FBS and likewise for NIS, where 10% FBS was used.

$$IC_{10,nom,baseline}(M)=\frac{0.026M}{D_{lip/w}}\times\left(1+D_{BSA/w^{\circ}}\times{}^{\circ}VF_{protein,medium}+D_{lip/w}{}^{\circ}\times{}^{\circ}VF_{lipid,medium}\right) \qquad (S18)$$

The toxic ratio TR (eq.S19) is the ratio between predicted ($IC_{10,baseline}$) and experimental cytotoxicity (IC_{10}).[53]

$$TR=\frac{IC_{10,baseline}}{IC_{10}} \qquad (S19)$$

The specificity ratio $SR_{baseline}$ (eq.S20) is the ratio between predicted baseline cytotoxicity ($IC_{10,baseline}$) and effect concentration (EC_{10} or $EC_{IR1.5}$).[42]

$$SR_{baseline}=\frac{IC_{10,baseline}}{EC_{10}} \quad \text{or} \quad SR_{baseline}=\frac{IC_{10,baseline}}{EC_{IR1.5}} \qquad (S20)$$

Text S6. Mixture predictions

The errors of the mixture predictions and related parameters were calculated with eqs. S21 to S24.

$$SE(REP_i)\approx\sqrt{\frac{1}{EC_y(i)^2}\cdot SE(EC_y(reference))^2+\frac{EC_y(reference)^2}{EC_y(i)^4}\cdot SE(EC_y(i))^2} \qquad (S21)$$

$$SE(BEQ_{chem})\approx\sqrt{\sum_{i=1}^{n}C_i^2\times SE(REP_i)^2+REP_i^2\times SE(C_i)^2} \qquad (S22)$$

$$SE(BEQ_{bio,mix})\approx\sqrt{\frac{1}{EC_y(mix)^2}\cdot SE(EC_y(reference))^2+\frac{EC_y(reference)^2}{EC_y(mix)^4}\cdot SE(EC_y(mix))^2} \qquad (S23)$$

$$SE(BEQ_{bio})\approx\sqrt{\frac{1}{EC_y(sample)^2}\cdot SE(EC_y(reference))^2+\frac{EC_y(reference)^2}{EC_y(sample)^4}\cdot SE(EC_y(sample))^2} \qquad (S24)$$

The index on prediction quality (IPQ)[54] is defined by eq. S25. An IPQ of 0 is a perfect agreement, and IPQ of 0.5 is the typical threshold for acceptable agreement with CA for equipotent mixtures, which corresponds to a factor of 2 (0.3 log units) ratio between experimental and predicted (or vice versa). Here we have mixtures in the concentration ratios as they were detected and some of the mixture components were inactive on their

own but might have contributed to the mixture nevertheless, therefore we relax the criterion to an IPQ of 0.9, which corresponds to one log unit deviation in either direction.

$$IPQ = 1 - \frac{IC_{10}(exp)}{IC_{10}(CA)} \text{ for } IC_{10}(exp) < IC_{10}(CA)$$

$$\text{and } IPQ = \frac{IC_{10}(exp)}{IC_{10}(CA)} - 1 \text{ for } IC_{10}(exp) > IC_{10}(CA) \quad (S25)$$

<u>Text S7. Quantified chemicals shared in the environment and in humans</u>

The heatmap depicted in Figure1 C was additionally clustered in both dimensions. Hierarchical Clustering was performed using the vegan R package (version 2.6-4). Manhattan distance was used for both binary and continuous variables. Clustering was done using the Maximum Linkage approach. Non-detects were replaced by 10^{-13}. With respect to chemicals, fish clustered at one end and serum extracts at the other end with water and milk in the middle but a subcluster of SW, EFF and WW was closer to serum. BW and DW were rather independent and separated fish from milk (Figure S2A). All milk clustered together with HBM adjacent, but separate, from cow milk.

With respect to the dimension of chemicals, fish extracts stuck out and clustered due to the presence of TCS, BP3, BPA and BPAF. It is possible that these are contaminations and stemmed from packing materials such as plastic crates or plastic film. Such consumer product and plastic chemicals were abundant and detected in all samples but BW and DW. Else there was no separation between industrial chemicals, endogenous compounds and pesticides, the different chemical classes were distributed over all clusters.

The effects clustered in a different fashion (fig S2B): Human serum, HBM and fish formed a large cluster where the groups were not well separated. DW/SW/BW clustered at the opposite spectrum with milk in a cluster in the middle and EFF between the milk cluster and the fish with a closer connection to the latter. The clustering of bioassays was not easy to rationalize with the three effect groups distributed over all larger clusters but genotoxicity clearly being the least responsive assay that was well separated from other assays.

Text S8. Quality assurance/quality control and reference compounds for all bioassays

In each assay, reference compounds were run alongside the tested samples, while for all samples all individual repeats were evaluated together. The quality assurance/quality control (QA/AQ) was performed accounting for the variability of individual repeats. The QA/QC measures follow the recommendations given previously.[55, 56]

Precision is expressed as the coefficient of variation for repeatability (CVr, eq. S26 and for reproducibility (CVR, eq. S27). μ_r is the mean and σ_r is the standard deviation of the validation sample results repeated on multiple occasions by the same operator. μ_R is the mean and σ_R is the standard deviation of the validation sample results repeated on multiple occasions by different operators and/or laboratories.

Both parameters should be <15-20% for an assay to be deemed repeatable and reproducible.

$$CVr = (\sigma_r / \mu_r) \quad (S26)$$

$$CVR = (\sigma_R / \mu_R) \quad (S27)$$

Robustness characterizes the sensitivity of a method to operational variations and is a measure of how transferrable the method is to other operators and/or laboratories. Robustness is usually calculated as the ratio (eq. S28) of the CVr over the CVR.

$$\text{Robustness index} = \frac{CVr}{CVR} \quad (S28)$$

As each assay was only run in one laboratory by one team, the robustness index was only derived as the ratio of CVr of the first experimental batch (extract) divided by the CVr of the second experimental batch (single chemicals and designed mixtures).

For reporter gene assays, the Z'-factor (eq. S29) is also reported as a measure of quality with a good assay having a Z'-factor between 0.5 and 1.

$$Z'\text{-factor} = 1 - \frac{3 \times (\sigma_p + \sigma_n)}{| \mu_p - \mu_n |} \quad (S29)$$

The limit of detection LOD and limit of quantification were calculated with eqs. S30 and S31, respectively.

$$LOD = \mu_n + (3 \times \sigma_n) \quad (S30)$$

$$LOQ = \mu_n + (10 \times \sigma_n) \quad (S31)$$

μ_n is the average response of the negative control and σ_n is the standard deviation of response with the negative control. Typically, the negative control are unexposed cells.

For bioassays, the "calibration curve" is the concentration-effect curve for the reference and other relevant compounds.

As bioassay results are often expressed in effect concentrations EC_{10} or $EC_{IR1.5}$ the LOD or LOQ are typically reported not as effects but as the EC of a reference compound, i.e., EC_{LOD}. The LOD expressed as a concentration is defined as the concentration of the reference chemical causing three times the standard deviation of the response of the negative control, which corresponds to eq. S32 for log-logistic concentration-effect curves, eq. S33 for linear concentration-effect curves with % effect and eq. S34 with induction ratio (IR) as the effect measure.

$$\log EC_{LOD} = \log EC_{50} - \frac{1}{slope} \cdot \log\left(\frac{max - min}{LOD - min} - 100\%\right) \tag{S32}$$

$$EC_{LOD} = \frac{LOD}{slope} \tag{S33}$$

$$EC_{IR,LOD} = \frac{LOD\text{-}1}{slope} \tag{S34}$$

As summary of the QA/QC data is given in Table S4.

In the **ZFE assay**, rather than treating positive chemicals, the response from non-treated zebrafish embryos were checked if they were in the acceptable range. Non-treated embryos were included for each assay plate, which showed only 2.5-5.0% of lethality.

In the **neurite outgrowth inhibition assay (NOI**) the reference compound narciclasine was run in four independent experiments in parallel to the characterization of the extracts (Figure S3A). The IC_{10} for 10% reduction of cell viability was 195 nM and the EC_{10} for 10% reduction of neurite length was 0.932 nM. They agree well with literature (IC_{10} 170 nM and EC_{10} 3.9 nM[57]). The variability of the cell viability control had a CV of 4.7%, and that of neurite length of 5.2%. The repeatability CVr was 8.9% for cell viability and 4.3% for neurite length. The Z'-factor was 0.85, indicating a good separation band (100% effect was assumed to have no variability as it could not be reached experimentally). The LOD was at 85% cell viability and 84% neurite length over all experiments in Table S7. Accordingly, the EC_{LOD} was 315 nM for cytotoxicity and 3.0 nM for reduction of neurite length.

For logistic reasons, the mixture had to be prepared in DMSO. All other experiments of extracts in the NOI were performed solvent-free because DMSO poses a great problem in the neurotoxicity assay, it has an EC_{10} in neurite outgrowth inhibition of

7.4 mM (data not shown). DMSO has a MW of 78.13 g/mol and a density of 1.1 kg/L, which means that the EC_{10} is at 0.05%. The mixtures had 1% DMSO in the background to be consistent with all other bioassays and because we could not prepare mixtures separately for this assay. The neurite length was overall very short with 1% DMSO background compared to solvent-free condition. Neurite length ranged from 1 ~ 16 mm/mm^2 in the exposed cells and 14~16 mm/mm^2 in the unexposed cells if there is no DMSO background. However, the neurite length was reduced in the presence of 1% DMSO to 1 ~ 6 mm/mm^2 in the exposed and 5~6 mm/mm2 in the unexposed cells: This means that the dynamic range is much smaller in the presence of 1% DMSO and therefore the EC_{10} values are less reliable. The IC_{10} (519 nM) and EC_{10} (5.9 nM) were also 2.7 to 6.3 times higher (lower sensitivity) in the presence of DMSO. Hence the effect data with DMSO cannot be compared to other experiments but for this study single compounds and mixtures were run under the same conditions and are therefore comparable.

The EC_{50} value of the reference compound paraoxon-ethyl in the **AChE inhibition assay** was 1.88 nM, which agrees with literature.[58] The repeatability CVr was 0.1%, the standard deviation of the controls was 4.95 % and accordingly the EC_{LOD} was 7.05 nM, which is higher than the EC_{50} due to the variability of the control. The concentration-response curves showed substantial heteroscedasticity and show much less variability at the higher inhibition levels (Figure S3B). The previously observed artifact of the AChE assay due to coextracted organic material was circumvented by the cell-based assay.[58] The fatty fish and serum samples cause visible cytotoxicity after 3h of exposure already, hence the detected effects are clear cytotoxicity artifacts, so any interference with the Ellman's reaction could not be picked up.

The EC_{10} value of *tert.*-Butylhydroquinone (tBHQ) in **AREc32 assay for oxidative stress response** were 2.7±0.07 µM during the measurements of the extracts and 1.2±0.02 µM during the designed mixture experiments (Figure S3C). The robustness was 2.1. The EC_{LOD} was 110.8 µM during measurement of the extracts and 4.9 µM during the mixture experiments. The robustness was 2.1 and no Z'-factor can be defined for an assay with induction ratio.

The EC_{10} value of azoxystrobin in the **mitochondrial toxicity assay** were 24.6 µM during the measurements of the extracts and 12.3 µM during the designed mixture

experiments (Figure S3D). These are similar as the method development paper with 15 μM[18] and robust during the two measurement series (robustness 1.52).

The natural THR agonist 3,3',5-trioiodothyronine (T3) was tested as reference substance in the **thyroid hormone receptor transactivation assay (THR-TA)** using a concentration series (n=3), in three experiments. In data analysis, calibration curves from all three experiments were combined to reach a single data set with 9 replicate values (Figure S3E), resulting in IC_{50} of 132 nM. This IC_{50} was higher than the IC_{50}=0.1 nM reported by Freitas et al.[19] Based on this single calibration curve, the following estimates were made: LOD=19.5 nM and Z'-factor=0.39. Endogenous ligands may interfere with the results for thyroid hormone assays. Biota extracts, specifically milk and blood, were expected to contain endogenous T4 hormone that could contribute to the assay result. Therefore, estimates were made for the contribution of T4 to the assay response. A contribution of endogenous T4 to the assay results cannot be excluded for TBG-binding by human breast milk (10%) and by serum samples (25%) and for THR-TA on cow milk. Lipids did not interfere with the TTR-binding results at the IC_{10} levels reported for the samples.

The natural TTR-ligand thyroxine (T4) was tested as reference substance for the **transthyretin (TTR) protein binding** assay and the measurements alongside the extracts agreed with those alongside the mixture experiments (Figure S3F) with a robustness of 0.7. The Z'-factor was 0.73± 0.09.

The natural TBG-ligand thyroxine (T4) was tested as reference substance in the **thyroxine-binding globulin (TBG) assay** using a concentration series (n=2), in twelve experiments (Figure S3G). Calibration curves from two independent experiments were combined to reach six data sets with 4 replicate values, resulting in an IC_{50} of 4.7 nM, which falls within the same range of values reported by Shen et al.[23] (9.2 [8.2-10.2], average [CI]). EC_{LOD} was 5.21 nM. The Z'-factor was determined to be 0.67± 0.25. The current application was the first time that the assay was used to test sample extracts, rather than pure compounds. At present, no method has been developed to correct anisotropy results for background fluorescence. Therefore, an arbitrary cut-off value of <20% autofluorescence was chosen: sample dilutions with >20% higher fluorescence than the background were excluded from data analysis.

For the **deiodinase isoenzymes types 1 to 3** (DIO1-3), propylthiouracil was used as positive control for DIO1 inhibition while xanthohumol served as inhibitor for DIO2 and 3 (Figure S3H-J). IC_{50} and EC_{LOD} are in Table S4. For DIO1, the average Z'-factor was 0.72, with no plates below the quality threshold of 0.5. The average CV of the 100 % control was 5.6 %, with one plate being above 10 %. For DIO2, the average Z'-factor was 0.49, with five plates out of twelve below the quality threshold of 0.5. The average CV of the 100% control was 9.9 %, with three plates being above 10 %. This was expected, as the specific activity of DIO2 is >10 fold lower in the used cell homogenate which results in prolonged incubation time (4 hours). Furthermore, the inhibitor Xanthohumol turned out to be unstable over time in aqueous solution. Therefore, positive controls on some plates had elevated activity, resulting in reduced dynamic and suboptimal Z'-factor. For DIO3, the average Z'-factor was 0.67, with one plate out of 14 below the quality threshold of 0.5. The average CV of the 100 % control was 6.5 %, with two plates being above 10 %.

For the **dehalogenase (iodotyrosin deiodinase) assay DEHAL1**, dibromotyrosine (DBT, FigureS2K) was used as specific inhibitor to define 0 % activity and had an IC_{50} of 242 nM and EC_{LOD} of 266 nM. The average Z'-factor for DEHAL1 was 0.68, with two plates out of 12 below the high-quality threshold of 0.5. The average CV of the 100 % control was 6.7 %, with one plate being above 10 %.

In the **sodium-iodide-symporter (NIS) assay**, sodium perchlorate ($NaClO_4$) was used as specific (competitive) inhibitor with a IC_{50} of the 356 nM and EC_{LOD} of 15.9 nM (Figure S3L). The uptake showed a small gradient over the microtiter plate, which was leading to a detecTable slope even in the blank extracts. While this gradient results from the washing procedure (loss of iodide), it is not evident in the viability readout from the identical cells. During the measurements of the extracts, the average Z'-factor for NIS was 0.67, with two plates out of 21 below the quality threshold of 0.5. The average CV of the 100 % control was 5.9 %, with four plates being above 10 %. On 30 plates run with the mixtures and pure compounds, there was an average Z'-factor of 0.66 and an average CV of <10% of the solvent control (100% activity).

The EC_{50} value of 2,3,7,8-tetrachlorodibenzo-dioxin (TCDD) in the **AhR CALUX** was 1.54 pM (Figure S3M), the EC_{LOD} was 0.62 pM and the Z'-factor 0.65.

The EC_{50} value of rosiglitazone as the reference compound in **PPARγ-GeneBLAzer** was 1.92 nM (Figure S3N), the EC_{LOD} was 0.25 nM and the Z'-factor 0.71.

Following the OECD Guidelines for the Testing of Chemicals, R1881 and OHF were used as reference compounds for **AR agonism and AR antagonism in the androgen receptor (AR) transcriptional activation assay**, respectively. The EC_{50} value for R1881 was 63.2 pM during the extract measurements and 53.2 pM during the mixture experiments (Figure S3P), which agrees with the OECD Guidelines (15 - 426 pM). In antagonist mode the IC_{50} for OHF was 34.7 nM during the extract measurements and 60.5 nM during the mixture experiments (Figure S3P), in line with the OECD Guidelines of 676 nM). The CV for repeatability (on log scale) were 0.08 %/0.26 % for R1881 and 0.26 %/0.23 % for OHF, while the estimated Z'-factors were 0.61 and 0.71, respectively. The robustness indices were 3.17 in agonist and 0.91 in antagonist mode (Table S4).

Following the OECD Guidelines for the Testing of Chemicals, 17β-estradiol (E2) was used as reference compound for ER agonism in the **estrogen receptor (ER) transcriptional activation assay (ER-Luc).** The EC_{50} value was 6.62 pM (Figure S3Q), which is in accordance with the OECD Guidelines (5.63 pM). The CV for repeatability was 0.13 %, while the estimated Z'-factor was 0.68.

The EC_{50} value of E2 in the **estrogen receptor (ER) transcriptional activation assay GeneBLAzer (ER-bla)** was 53 pM (Figure S3R), the EC_{LOD} was 3 pM and the Z'-factor 0.73.

All-trans retinoic acid (ATRA) was used as reference compound in the **Plurilum** assay for the inhibition of cardiac differentiation of hiPSCs-based embryoid bodies. ATRA was previously shown to be a strong teratogen in stem cell-based models used for cardiac development assessment.[59, 60] The estimated IC_{50} value was 15.2 nM (Figure S3S). The CV for repeatability was 0.12 % (for $logEC_{50}$), while the Z'-factor was 0.59.

Four reference compounds were run in one constant concentration in all the independent experiments in the **γH2AX and pH3 assays for genotoxicity** (Figure S3T-U) and matched with expectation. The negative control 0.2% DMSO had no effect. The clastogen compound that requires a metabolic bioactivation (1 µM benzo[a]pyrene (BaP)) was only activating γH2AX in the metabolically active HepG2 cells. The direct clastogen 10 µM Etoposide (Eto) activated γH2AX in both two cell lines. The aneugenic 1 µM Nocodazole was activating pH3 in both cell lines.

Text S9. Cytotoxicity of sample extracts

The inhibitory concentrations decreasing the cell viability by 10%, IC_{10}, were consistent across all cell lines (Table S5) although cytotoxicity could not be recorded in all cell lines (Tables S6-S26).

All cells were exposed for 24h with the sample extracts, apart from the 3h exposure in the AChE assay, which showed no cytotoxicity and was omitted from the further cytotoxicity evaluation, and apart from the 7-day exposure in PluriLum. In contrast, the cells used in the NIS assay showed cytotoxic effects after 30 min of incubation, qualifying the used assay type (ATP quantification) as rapid readout for cytotoxicity. For HepG2 and SH-SY5Y cells that were used in the genotoxicity assay with an IC_{50} cut-off, the IC_{10} was also calculated and used here for comparison with other cytotoxicity IC_{10}.

In all water samples cytotoxicity was highest in WW (IC_{10} ranging from 0.39 to 3.1 $mL_{water}/mL_{bioassay}$), followed by EFF (IC_{10} ranging from 10 to 56 $mL_{water}/mL_{bioassay}$), and SW (IC_{10} ranging from 23 to 153 $mL_{water}/mL_{bioassay}$). In general, DW had some low effects at higher than REF 100 $mL_{water}/mL_{bioassay}$ (IC_{10} in five active samples ranging from 64 to 144 $mL_{water}/mL_{bioassay}$) but bottled water had an IC_{10} of 279 $mL_{water}/mL_{bioassay}$ in one single assay (AR agonism), which is negligible (Table S5). The blank did not show any cytotoxicity up to REF 300 $mL_{water}/mL_{bioassay}$.

Cytotoxicity of the fish extracts was highest in FFW, followed by FFA and LFW, the blanks were clean. There was little variation between the different assays for the same sample with exception of ZFET and the PluriLum assay, which are more complex systems than all other 2D cells.

Cow milk showed only very low cytotoxicity, while human breast milk was much more cytotoxic (Table S5). These differences can also be caused by the different extraction methods: Cow milk was delipidated with hexane, which has likely also removed some of the hydrophobic pollutants, while the EMR clean-up used from HBM is likely to be much milder. The concentrations of legacy persistent organic pollutants were negligible in cow milk and therefore, differences in the burden of chemical contamination between cow and human milk is also possible.

The five different serum types showed cytotoxicity with less than a factor of three difference between the mean IC_{10} values of the serum samples (Table S5).

Text S10. Fish embryo toxicity

While WW showed the highest mortality in zebrafish embryos, BW and DW did not show any effects on mortality of zebrafish embryos within the tested concentration range (CRCs deposited in https://doi.org/10.5281/zenodo.20402074). In case of fish, FFW caused the strongest effects on mortality followed by FFA and LFW (Table S5). HBM was the only sample which exhibited effects on mortality among milk samples, and the rest of the milk samples as well as blanks were inactive. All 5 serum samples (S1-S5) exhibited more than 70% of mortality for all tested concentration range, hence LC_{10} for these samples could not be determined.

Behavioral experiments were performed for active samples at LC_{10} concentrations. LFW highly decreased STC but none of the tested samples significantly affected STC after 24 h of exposure (Figure S4A). When PMR was observed after 30 h exposure, WW and LFW decreased PMR (Figure S4B). All milk samples and the milk process blank decreased PMR at excitation phase. Similar trends were observed for LMR (Figure S4C), where WW and LFW highly and consistently decreased the activity over different phases. For milk samples, effects did not differ from the blank. Regarding morphological changes, decrease in swim bladder area was pronounced for WW and LFW (Figure S4D). Even stronger decrease was observed for OCM, and no swim bladder was observed in zebrafish embryo after exposure to CCM. Considering that no obvious malformation was observed for these samples, the effects on LMR was likely to be no secondary effects from morphological defects and rather more due to effects on nervous system. Lastly, for heart rate, no obvious effects were observed and only FFW significantly decreased heart rate by 9% (Figure S4E).

Overall, WW and LFW consistently caused adverse effects in behavioral tests, which indicates that they might cause developmental neurotoxicity but due to the limited availability of extract the behavioral assays could only be performed at concentration corresponding to the LC_{10}.

Text S11. Process and serum blanks

The process blanks had negligible effects (Table S5), and therefore a blank subtraction was not necessary. An exception was the milk blank BM2, which turned out to have been run with contaminated glassware. Therefore, an additional two milk blanks were run (BM3 and BM4) and these were not problematic.

It is a different case with the serum samples. A previous study on the neurotoxicity bioassay has demonstrated that plasma has a background effect in this bioassay.[61] In this previous study we stripped plasma with charcoal, which completely removed all chemicals as spiking experiments demonstrated.[61] As it was not possible to strip the serum samples, we used commercially available charcoal-stripped fetal bovine serum (cs-FBS) as a proxy of human serum and ran it through the same SPE as the serum samples. There is a difference in the protein content of FBS, which is about half of that of human serum. However, as we are not certain if the serum background effect stems from the proteins or other organic chemicals (growth factors, etc.), we used the cs-FBS at the same REF as the serum samples. The blank effect was subtracted with eq. S35 for those serum extracts that showed activity.

$$EC_{10}(\text{blank-corrected})=\left(\frac{1}{EC_{10}(\text{sample})}-\frac{1}{EC_{10}(\text{cs-FBS})}\right)^{-1} \quad \text{(S35)}$$

For SH-SY5Y, the blank cytotoxicity ranged from 1.4 to 4.0% of the measured cytotoxicity but for the NOI endpoint from 6.3 to 14.7% (Table S5). Blank correction was also necessary for AREc32 where 14 to 47% of ARE activation was caused by the serum blank (Table S5). This was different for THR-TA, where the blank effects were only 0.7 to 3.5% and were therefore also negligible. In case of TTR, the cs-FBS had an EC_{10} of 0.165 REF. As the CRC of TTR was a log-logistic fit, the blank correction would be concentration-dependent if the logCRCs were not parallel. The slope ratios between sample and cs-FBS ranged from 1.3 to 1.5 and therefore a close enough to parallel slopes that we deemed it reasonable to simplify and apply also eq. S35. The blank activity amounted to 4% to 57% of the effect of the serum extracts for DOI1, DOI2, DOI3 and typically it is not recommended to make a blank correction if the blank constituted more that 50% of the effect of the same (effect meaning here $1/EC_{10}$). DEHAL was less affected by the blank (12% to 40%) and for NIS the blank correction was almost insignificant (3% to 7%).

Text S12. Detailed results on genotoxicity of extracts

The γH2AX/pH3 assay was performed on two human cell lines: the hepatocellular line HepG2 and the neuronal line SH-SY5Y. Both cell lines were exposed during 24 hours to the extracts, chemicals or synthetic mixtures, followed by the γH2AX/pH3 assay with the In-Cell Western technique of parallel detection of cytotoxicity and the genotoxicity. Moreover, this assay permits the distinction of the two genotoxic modes of action (MoA): the clastogenic MoA for samples that induce direct or indirect DNA damage and the aneugenic MoA for samples that cause a change in the number of chromosomes.

Genotoxicity was considered positive when a sample produced a statistically significant 1.5-fold γH2AX or pH3 induction at a level of cytotoxicity below 50% compared to the control. This is why we relied on the IC_{50} and not on the IC_{10} as cytotoxicity cutoff. These parameters were based on our previous studies,[38] and are generally accepted for use within genotoxicity assessment.[43]

For water samples, DW, BW and SW did not induce genotoxic nor cytotoxic effects in the two cell lines used up to REF 300 $mL_{water}/mL_{bioassay}$. In contrast, WW induced genotoxic effect with an increase in the induction of the phosphorylation of H2AX without effect on pH3 biomarker and without cytotoxicity. We observed an increase in the induction of the γH2AX biomarker of EFF at REF 100 $mL_{water}/mL_{bioassay}$ on the HepG2 cell line and at REF 66 $mL_{water}/mL_{bioassay}$ on the SH-SY5Y cells without cytotoxic effect (IC_{50} > 300 $mL_{water}/mL_{bioassay}$). No variation in the induction of pH3 was observed with this sample. WW showed an increase in the phosphorylation of this same biomarker (γH2AX) of REF>1 in the two cell lines studied, without cytotoxic effect (IC_{50} > 4).

For all the milk samples tested (OCM, CCM and HBM), no genotoxic nor cytotoxic effects were observed on the two cell lines used, with $EC_{IR1.5}$ and EC_{50} > REF 1 $mL_{milk}/mL_{bioassay}$.

For fish samples, the lean fish (LFW) did not induce genotoxic and cytotoxic effects in the two cell lines used with $EC_{IR1.5}$ and EC_{50} > REF 0.5 $g_{fish}/mL_{bioassay}$. With the FFA, we observed an increase in the induction of γH2AX at REF 0.5 on the HepG2 cell line, whereas we obtained a cytotoxic effect with this sample on the SH-SY5Y cell line with a IC_{50} to 0.2 $g_{fish}/mL_{bioassay}$. The sample FFW caused cytotoxicity in both cell lines from REF 0.2 $g_{fish}/mL_{bioassay}$. No variation in the induction of pH3 was observed with these samples.

The serum samples were cytotoxic with IC_{50} ranging from REF 0.35 to 0.80 $mL_{serum}/mL_{bioassay}$ for HepG2 and IC_{50} ranging from REF 0.62 to 1.41 $mL_{serum}/mL_{bioassay}$ for SH-SY5Y but did not activate γH2AX or pH3.

Text S13. Detailed results of testing of extracts for neurotoxicity

All water samples caused inhibition of neurite outgrowth with EC_{10} values of REF 2.3 $mL_{water}/mL_{bioassay}$ for WW, 6.2 for EFF, 15 for SW, 35.5 for DW and 271 for BW and the blanks BWSPE and BWF were not active up to REF 300. REF 2.3 means that the sample WW had to be enriched 2.3 times to trigger 10% effect, and REF 271 means that the sample BW had to be enriched 271 times to trigger the effect. A 10% effect at a higher REF means an overall lower activity than an EC_{10} at a lower REF.

All samples, with exception of BW, caused cytotoxicity, albeit at IC_{10} higher than EC_{10} and correspondingly specificity ratios (SR = IC_{10}/ EC_{10}) ranging from 3.9 to 7.4. This is a typical specificity ratio for water samples indicating no dominance of mixture effects by specific neurotoxicants.

The EC_{10} agreed well with previous studies on European WWTP effluents and surface water.[62] In fact, the EFF was very close to the mean value of 56 European WWTP effluents.[62] The pooled European SW sample showed lower effects than the literature data but previous samples were often taken during rain events,[62] which typically have higher effects than samples taken in rivers at dry weather.

The fish samples showed cytotoxicity and neurotoxic effects with EC_{10} values of REF 0.022 to 0.06 $g_{fish}/mL_{bioassay}$ and a SR of 2.6 to 3.7. The fatty fish showed higher neurotoxic effects than the lean fish. All blanks were nontoxic up to the highest enrichments tested. Cow milk had EC_{10} for NOI of REF 1.2 $mL_{milk}/mL_{bioassay}$ and showed no cytotoxicity. The HBM was almost 10 times more potent, but was also extracted with a different method, so a direct comparison between cow and human milk may be difficult. The serum samples had an exceedingly high effect on the neurite outgrowth with low cytotoxicity and SR from 9 to 28. Note that also the charcoal-stripped FBS that was used to correct for the background effects had a SR of 45.

AChE was not tested with the serum extracts as AChE inhibitors are readily metabolized in the body and typically occur at concentrations too low to be detectable.

The MitoOxTox assay was performed with AREc32 cells, and the measurement of the mitochondrial membrane potential was multiplexed with cytotoxicity and oxidative stress response. The SPE blank did not show any effect up to an REF of 300. WW showed the highest toxicity for all three endpoints among the five types of pooled water extracts. EFF affected MMP with EC_{10} of REF 0.3, which means the sample had to be diluted by a factor of 3 to disrupt 10% MMP, while the $EC_{IR1.5}$ for oxidative stress response was at REF 1.20 and cytotoxicity occurred only at an IC_{10} of 5.38, which is 4 to 20 times less sensitive than the MMP endpoint. WW samples showed specific effects on MMP with $SR_{cytotoxicity}$ of 19.2 and specificity on oxidative stress response with $SR_{cytotoxicity}$ of only 4.5.

EFF needed to be enriched more than 10 times to show effects on oxidative stress response and more than 30 times for cytotoxicity. While a EC_{10} of 62 for MMP could be derived for the EFF, this effect occurs at higher REF than cytotoxicity and is therefore not specific ($SR_{cytotoxicity}$ = 0.5). In contrast, the oxidative stress response shows a $SR_{cytotoxicity}$ of 2.9. Hence, the pooled WW was not only much more potent than the EFF but also more specific on MMP (Figure 2). In terms of oxidative stress response, the WW had a higher response than the EFF but $SR_{cytotoxicity}$ was similar and rather low for both types of samples.

Surface water, drinking water, and bottled water did not show any effects up to REF 50 and had to be enriched more to exert cytotoxic effects (IC10 of REF 55 for surface water, 87 for drinking water and >300 for bottled water).

The oxidative stress response in the water samples aligned well with literature data[62-64] with the WW causing more effect than most previous samples and the DW and BW showing smaller effects but in previous studies only chlorinated waters have been tested, while the pooled European DW samples contained pooled non-disinfected and disinfected water, and BW is typically not disinfected.

Fish extracts also activated oxidative stress response with an $EC_{IR1.5}$ of 0.022 to 0.051 $g_{fish}/mL_{bioassay}$ and milk with $EC_{IR1.5}$ of 3.07 $mL_{milk}/mL_{bioassay}$ for OCM and 2.44 $mL_{milk}/mL_{bioassay}$ for CCM. Serum samples were active but not highly specific apart from S4, the human cord blood with a SR of 25 and an $EC_{IR1.5}$ of 0.091 $mL_{serum}/mL_{bioassay}$.

Text S14. Detailed results on thyroid hormone system disruption of extracts

Out of all water, fish, and milk samples tested (extracts and blanks) only the conventional cow milk extract (CCM) exhibited agonistic activity towards the thyroid hormone receptor in the THR-TA assay, with an IC_{10} of REF 0.33. Cytotoxicity was observed for the wastewater extract and for the three fish extracts. For WW, an IC_{10} for cell viability was determined of REF 2.6 $mL_{water}/mL_{bioassay}$. For the fish extracts, however, no sigmoidal concentration-response curve could be fitted, due to a non-monotonic cytotoxic response, with cell viability in the middle concentrations being lower than in the low and high concentrations. Consequently, no IC_{10} could be determined.

Weak TTR-binding responses were found for both water blanks, i.e., BWSPE (IC_{10} of REF 108 $mL_{water}/mL_{bioassay}$) and BWF (IC_{10} of REF 167 $mL_{water}/mL_{bioassay}$). Water blank responses, however, were negligible at IC_{10} values determined for the water samples, for which IC_{10} decreased in the order BW (REF 33 $mL_{water}/mL_{bioassay}$) ≈ DW (REF 31) > SW (REF 5.1) > EFF (REF 1.3) >> WW (REF 0.080).

For cow milk blanks, TTR-binding responses were observed, with IC_{10} ranging from REF 0.29 to 0.32 $mL_{milk}/mL_{bioassay}$. Similar as for the water blanks, the cow milk blank responses were negligible at IC_{10} values determined for the CCM (REF 0.012 $mL_{milk}/mL_{bioassay}$) and OCM (REF 0.010 $mL_{milk}/mL_{bioassay}$) samples. In contrast, no TTR-binding was observed for the human breast milk blank (BHBM), while the TTR-binding capacity of the actual HBM sample was one order of magnitude higher than in cow milk samples OCM and CCM (IC_{10} of REF 0.0018 $mL_{milk}/mL_{bioassay}$).

Fish blanks only gave a TTR-binding response at the highest test concentration, with IC_{10} values ranging from REF 1.0 to 1.1. For fish extracts, however, IC_{10} values were three orders of magnitude lower, i.e., REF 0.0011 $g_{fish}/mL_{bioassay}$ for LFW, REF 0.00071 for FFW, and REF 0.00077 for FFA.

For the blank blood collection bag (BBCB) extract, interference was observed with the FITC-T4 substrate. This interference led, in the presence of TTR, to an apparent IC_{10} value of REF 0.084, which should be attributed to increased fluorescence of FITC-T4 in the presence of the extract, and not to components in the extract competing with FITC-T4 for TTR-binding. For the actual serum extracts, TTR-binding was observed at lower REF than the charcoal-stripped FBS, with a little higher TTR-binding potency in the Australian samples (IC_{10} at REF 0.00063 $mL_{serum}/mL_{bioassay}$ for both S2 and S3) compared

to the Danish samples (IC_{10} at REF 0.0011 $mL_{serum}/mL_{bioassay}$ for S1 (adult) and REF 0.0014 $mL_{serum}/mL_{bioassay}$ S4 (cord blood)).

For the water extracts, TBG binding activity was only observed for the WW sample, with an IC_{10} value of REF 0.66. None of the blank samples exhibited any TBG binding activity. TBG binding capacity of the HBM extracts (IC_{10} of REF 0.0052 $mL_{milk}/mL_{bioassay}$) was at least one order of magnitude higher than observed for cow milk samples OCM (IC_{10} of REF 0.094 $mL_{milk}/mL_{bioassay}$) and CCM (IC_{10} of REF 0.19 $mL_{milk}/mL_{bioassay}$), similar as was observed for the TTR-binding assay. For the fish extracts, higher TBG binding activity was observed for FFW (IC_{10} of REF 0.00098 $g_{fish}/mL_{bioassay}$) compared to LFW (IC_{10} of REF 0.0031 $g_{fish}/mL_{bioassay}$) and FFA (IC_{10} of REF 0.0027 $g_{fish}/mL_{bioassay}$). All serum extracts demonstrated TBG binding capacity, being slightly higher in the Australian samples (IC_{10} at REF 0.0040 $mL_{serum}/mL_{bioassay}$ for S2 and REF 0.0044 $mL_{serum}/mL_{bioassay}$ for S3) compared to the Danish samples (IC_{10} at REF 0.011 $mL_{serum}/mL_{bioassay}$ for S1 (adult) and REF 0.0076 for S4 (cord blood)). Similar as to the milk samples, this reflects the findings for the TTR-binding assay.

All three deiodinases were affected by WW in a range of REF 2 to 4 $mL_{water}/mL_{bioassay}$, having the strongest effect on DIO1 activity. Internal discussion led to the hypothesis, that a) humic acid as partially coextracted matrix in environmental waters[65] might contribute to this effect and b) that the effect size might depend on the overall yield of the enzymatic reaction, to more or less suppress unspecific protein-interaction of present humic acid with the enzyme. Experiments with humic acid standard confirmed a direct effect of humic acid on DIO1 activity. To suppress this unspecific protein interaction, BSA was tested as scavenger and the results confirmed that addition of defined, enzymatically inactive protein might provide a future strategy to suppress humic acid effects using cell-free bioassays.

Fish extracts had a strong inhibitory effect on all three deiodinase isoenzymes, e.g., with an EC_{10} of REF ~0.0005 $g_{fish}/mL_{bioassay}$ for DIO1/FFW or ~0.001 $g_{fish}/mL_{bioassay}$ for DIO3/LFW. In addition, the serum extracts had a high inhibitory potential for all isoenzymes, e.g., with an EC_{10} of REF ~0.05 $mL_{serum}/mL_{bioassay}$ for DIO1/S3 or ~0.01 $mL_{serum}/mL_{bioassay}$ for DIO3/S1. There is a high probability that these effects of the different categories of sample types might be due to unspecific interaction from major matrix compounds, rather than being a specific effect of extracted anthropogenic chemicals. For

the HBM extract, strong effects were detected, again in all applied cell-free assays, while the OCM had a strong effect only on DIO1 (and DEHAL1), showing a certain degree of specificity. Interestingly, for CCM no effect was detected in any of the cell-free assays, providing evidence for a certain degree of matrix independence of OCM and HBM associated inhibitions. A direct effect of the extracts on the final readout (Sandell-Kolthoff-reaction) also cannot be ruled out and might deserve further attention.

The dehalogenase cell-free assay showed a pattern of inhibition very similar to the iodothyronine deiodinase isoenzymes, while representing a completely different type of enzyme regarding structural and biochemical homology. WW was the only environmental water sample showing an inhibitory effect with an approximated EC_{10} of ~REF 1.7. Fish and serum extracts exerted a strong inhibition (e.g., with an EC_{10} of REF ~0.006 for DEHAL/FFW $g_{fish}/mL_{bioassay}$ or ~0.05 $mL_{serum}/mL_{bioassay}$ for DEHAL1/S1). Co-incubation with HBM and OCM, in contrast to CCM extract, lead to a significant inhibition (with an EC_{10} of REF ~0.2 $mL_{milk}/mL_{bioassay}$ for OCM and ~0.01 for HBM $mL_{milk}/mL_{bioassay}$). The similar patterning of this enzyme, compared to the results for the DIO isoenzymes, might argue for a more “general” effect, more depending on the matrix type/respective major compounds that are co-extracted, rather than on low level contaminants. Again, direct effect of the extracts on the final readout (Sandell-Kolthoff-reaction) cannot be ruled out.

While the majority of extracts (WW, EFF, FFW, FFA, LFW, OCM, CCM, HBM, S1-S5) had a clear effect on the cellular NIS activity within the studied REF ranges, this was accompanied by a respective reduction in viability, which is not a consequence of NIS inhibition but rather occurring alongside, likely triggered by other mixture components in the extracts. While the cellular toxicity did not completely mask the effects on NIS-depending transport regarding the potency in most cases (e.g., EC_{10} of REF ~0.6 $mL_{milk}/mL_{bioassay}$ and IC_{10} of REF ~2.8 $mL_{milk}/mL_{bioassay}$ for CCM), the short incubation time (30 min) could lead to an underestimation of the true cytotoxic potential.

Text S15. Detailed results on reproductive toxicity of extracts and comparison with literature

The AR-EcoScreen™ was used to assess the AR agonistic potential of water samples, and the AR antagonistic potential of fish, cow milk, human breast milk, human adult, and human cord blood serum samples. AR antagonism was measured in the presence of 0.1 nM methyltrienolone (R1881). Data were normalized to a negative control (untreated cells) and a reference compound (R1881 for AR agonism, and 2-hydroxyflutamide (OHF) for AR antagonism).

Of all tested water samples, only WW and EFF showed AR agonistic activity within a non-cytotoxic range of concentrations, with EC_{10} values of REF 0.027 $mL_{water}/mL_{bioassay}$ for WW and REF 1.83 $mL_{water}/mL_{bioassay}$ for EFF. Wild fish samples (FFW and LFW) displayed over 2-fold higher antiandrogenic potential when compared to the aquaculture sample (FFA). OCM and CCM, as well as HBM, exhibited antagonistic activity towards AR, with IC_{10} values of REF 0.65 $mL_{milk}/mL_{bioassay}$, REF 1.80 $mL_{milk}/mL_{bioassay}$, and REF 0.084 $mL_{milk}/mL_{bioassay}$, respectively. Amongst the pooled serum samples, the adult female (childbearing age) Australian serum (S3), and the Danish cord blood serum (S4) showed the highest antiandrogenic potential (IC_{10} at REF 0.082 $mL_{serum}/mL_{bioassay}$ for S3 and REF 0.12 $mL_{serum}/mL_{bioassay}$ for S4). No significant effect was noted for blanks, aside from BM1, BM2 and BM3. Regarding cytotoxicity, samples and blanks either showed no significant effect in cell viability, or the IC_{10} values for cytotoxicity were higher than the estimated REF for (anti)androgenic effect.

Apart from DW and water blanks, pooled water samples showed supramaximal estrogenic effect in the ER_Luc, with observed maximal effects being 2 to 4-fold higher than the maximal effect of E2. The estrogenic potency of water samples, based on their EC_{10} (ranging from REF 0.004 to REF 53.36), was as follows: WW > EFF > SW > BW > DW. Wild fish samples (FFW and LFW), but not FFA, exhibited estrogenic activity at non-cytotoxic REFs (EC_{10} of REF 0.11 for FFW and REF 0.10 $g_{fish}/mL_{bioassay}$ for LFW). Cow milk samples showed agonistic effects towards ER as well, with EC_{10} values of REF 1.30 $mL_{milk}/mL_{bioassay}$ for OCM and REF 2.25 $mL_{milk}/mL_{bioassay}$ for CCM. No effect was observed in HBM other than cytotoxicity. Both adult and cord blood serum samples from Denmark showed significant ER agonist activity (EC_{10} of REF 0.074 $mL_{serum}/mL_{bioassay}$ and REF 0.25

$mL_{serum}/mL_{bioassay}$, respectively). Besides HBM, cytotoxicity was observed in WW, FFA and LFW within the tested REF ranges.

For comparison, the ER_BLA assay was also run with the water samples because it has been more often applied in the past than the ER_Luc assay for water quality testing.[56] There was a good agreement between ER_BLA and ER_Luc at low effects (high EC_{10}) but ER_Luc was more sensitive than ER_BLA at high effects (low EC_{10}) (Table S5). Subsequent experiments with fish, milk and serum were therefore only performed with ER_Luc. The pooled European samples appear to be quite representative of the range of effect concentrations ER_BLA from literature.[62, 63, 66]

EFF, SW, DW, LFW, OCM and CCM exhibited some potential to inhibit the differentiation of the embryoid bodies at non-cytotoxic REFs, with EC_{10} values ranging from REF 0.035 $g_{fish}/mL_{bioassay}$ for LFW to REF 22.53 $mL_{water}/mL_{bioassay}$ for DW. S1, S2 and S3 showed effect at extremely low REFs (0.0024, 0.0016, 0.0025 $mL_{serum}/mL_{bioassay}$), which was unexpected and might require further quality control assessment for potential matrix effects. Some effects were noted for several blanks, including BWF, BF1, BF2, and milk blanks (BM1-BM4), though at higher REFs than the estimated EC_{10} values for effect of the respective samples, with exception of CCM. Regarding cytotoxicity, several samples and blanks reduced cell viability within the tested REFs, including HBM, serum samples, and respective blanks. For HBM and BHBM it was not possible to estimate potential effect on cell differentiation due to high variability of data. For WW, FFW and FFA, cytotoxicity was observed before any effect was detected in NKX2.5 inhibition.

Text S16. Effects of individual chemicals and TR and SR analysis

Dosing ranges for the individual chemicals were adjusted so that all chemicals should reach cytotoxicity, and no false negative effects would be reported due to dosing that was too low. This was necessary because of the high diversity of the chemicals not only in terms of their source and use category but also in terms of their physicochemical properties (Table S27). Most chemicals are neutral, but TeCP is almost fully anionic at pH 7.4, 4NP 60% anionic, and BPS shows a complex speciation with 39% neutral, 53% anionic and 9% cationic (Table S27, Figure S10A). The hydrophobicity expressed as K_{ow} ranges over 11 orders of magnitude and even after correcting for speciation, the binding to lipids and proteins (Figure S10A). Accordingly, the predicted baseline toxicity (eq. S17) also ranged over eight orders of magnitude (Figure S10B). A constant dosing would have led to false negative outcomes or the need for many repeats until the correct dosing range would be found.

Despite optimization of the experiments and targeting baseline toxicity, not all chemicals were soluble enough and consequently did not trigger cytotoxicity in all cell lines. In the neurotoxicity assay, EP, DDE, TEHT, ATZ, ATZOH and E1 did not trigger any cytotoxicity up to their solubility limit. They precipitated in the experiments if dosed at three times the predicted baseline toxicity and their concentrations could not be further increased.

The median experimental cytotoxicity IC_{10} did not vary much from each other (Figure S10C). Differences in IC_{10} between cell lines were subtle and mainly due to differences in protein content of the media, therefore we report in the main manuscript the median cytotoxicity but give an account of each cell line's cytotoxicity towards the individual chemicals in the following:

Only three compounds were specifically cytotoxic: CBZ had a toxic ratio TR (eq. S19) of 18 in SH-SY5Y (Figure S10D, Table S29), 118-235 in AREc32 SY5Y (Figure S10E, Tables S30-31), was not cytotoxic in NIS (Table S33) and had TR 5 in CHO cells (Figure S10G, Table S34). CBZ is a fungicide and has no specific mode of action but often fungicides impair cell growth. The TR of Caf ranged between 5 and 55, which might be somewhat uncertain as the physicochemical properties were only estimated. TeCP had TR between 4 and 26, which is expected because TeCP is an uncoupler in mitochondria.

All chemicals with exception of EP, pTSA and ATZOH affected the neurite length. However, their EC_{10} were still within the range of cytotoxicity. Only BPS had a specificity ratio $SR_{cytotoxicity}$ (eq. S16) over 10. This means that many of the chemicals were neurotoxic, and the neurite outgrowth was affected at lower concentrations than the overall cell viability, but the effects were not highly specific, and most chemicals could be classified as baseline toxicants even for the neurite outgrowth inhibition endpoint.

Only AA and BP3 triggered oxidative stress response (Table S30) but at baseline toxicity concentrations, hence the effects were not specific. In AREc32 most chemicals were cytotoxic but only CAF and CBZ had a TR >10 for both of the cytotoxicity measures (Table S30 for phase contrast imaging only (confluency), Table S31 for phase contrast imaging with nuclei staining). The mitochondrial membrane potential (MMP) was inhibited by 18 chemicals but only BPS had a SR of 37 (Table S31). In the main manuscript, we therefore focus exclusively on the endpoint MMP.

TTR is a cell-free assay and therefore the IC_{10} for TTR binding (Table S32) could not be compared with the baseline toxicity QSAR as this model is only valid for cytotoxicity after 24h of exposure. Still, the IC_{10} for TTR binding correlated with $D_{lip/w}$(pH 7.4). In principle, this is not surprising, as the T4 binding site is hydrophobic. There would be an alternative ANSA-based assay that uses ANSA as a substrate to measure competitive binding. ANSA in no way resembles T4, it is only indicative for a hydrophobic protein binding site. This way one could differentiate specific from non-specific TTR binding.

The NIS inhibition was already detected after 30 min. Therefore, the baseline toxicity QSAR is strictly speaking not valid as it is derived for 24h exposure. However, we can assume that the test chemicals reach steady state within the exposure time. Then a comparison would be possible because baseline toxicity is a reversible effect. Cytotoxicity and NIS inhibition were dependent on the hydrophobicity and all data fell within the baseline prediction range (Table S33). 20 chemicals inhibited NIS. CAF and ATZOH were cytotoxic and only pTSA and CBZ showed neither cytotoxicity nor NIS inhibition.

Out of the 24 quantified chemicals, 21 were tested positive for AR antagonism with AA, CBZ and testosterone as the exceptions (Table S34). In case of the androgen receptor (AR) antagonism, all chemicals with exception of Test were baseline cytotoxic, but many were specifically antagonizing the AR. The specificity of AR antagonism was especially high for PRO with $SR_{baseline}$ of 8652. All single chemicals were tested for AR

agonism, but there was no effect for chemicals other than the hormones (Table S34). All hormones were activating the AR with high $SR_{baseline}$ (<1 Mio for Test) and not cytotoxic.

It was not possible to derive IC_{10} for the genotoxicity endpoints γH2AX_Neu and pH3_Neu (Table S35), although often the highest tested concentration was active but also slightly cytotoxicity even if the cytotoxicity threshold of 50% that is exceptional for genotoxicity assays, was not exceeded.

Text S17. Effect-based trigger values (EBT)

EBTs are bioassay-specific and are typically derived by reading across from established environmental quality standards for single chemicals. Several methods for the derivation of EBT for surface water and drinking water are reviewed in Escher et al.[56] As the database of single chemical effect data is not complete, we have used an interim method that relies on a good correlation between existing EBTs and the effect concentrations of the reference chemicals.[67] For surface water the SW-EBT was approximated to be 5.7 times lower that the EC_{10} of a typical reference compound (eq. S36) and a factor of 0.7 for drinking water (eq. S37).

$$\text{SW-EBT}=\frac{EC_{10}}{5.8} \quad (S36)$$

$$\text{DW-EBT}=\frac{EC_{10}}{0.7} \quad (S37)$$

We adopted the same EBT for BW as for DW and assumed that effluent is typically 10 times diluted in the receiving stream (eq. S38) and the WWTP can remove 90% of the mixture effect (eq. S39). The resulting EBT for each bioassay are listed in Table S37.

$$\text{Eff-EBT}=\frac{EC_{10}}{0.58} \quad (S38)$$

$$\text{WW-EBT}=\frac{EC_{10}}{0.058} \quad (S39)$$

All BEQ_{bio} of all water samples in all bioassays with exception of ER-Luc were below their EBT-BEQ (Figure S12). DW and BW were typically substantially lower that the EBT WW, EFF and SW were only about a factor of ten below EBT. ER-Luc was consistently above the EBT for WW, EFF and SW despite the EBT derived with the interim method (0.31 ng/L) was similar to the previously derived EBT of 0.62 ng/L for VM7Luc ER TA.[68] However, the EBT was not exceeded for the second estrogenicity assay ER-GeneBLAzer for these three water samples. The ER-GeneBLAzer was 13 to 50 times less sensitive than the ER-Luc but the EBT only differed by a factor of 2.5, which explains the differences in passing of the EBT threshold or not.

There are no EBTs available for food in the literature. We derived tentative EBTs for the four bioassays (NOI, MMP, TTR, NIS) for which we had the 24 individual chemical effect data. We started with safe concentrations for single chemicals, i.e., Acceptable Daily Intakes (ADI), Reference Doses (RfD) or Derived No Effect Levels (DNEL). ADI, RfD or DNELs were sourced from the ECHAchem database[69] or the COMPTOX

Chemistry Dashboard (Table S27).[70, 71] The allocation factor AF is approximately 20% for fish and for dairy products[72] and we assumed that 50 g_{fish}/d or 700 mL_{milk}/d were ingested daily by a 70 kg adult.[73] The resulting concentrations for each chemical i at its ADI/RfD/DNEL were converted to BEQ_i by multiplying by the relative effect potency REP_i (eq. S40). These BEQ_i were log-normally distributed for all four bioassays (data not shown) and the 50th percentiles of these distributions (i.e., medians) were used as EBT-BEQs.

$$BEQ_i = REP_i \times AF \times DNEL \times \frac{\text{body weight}}{\text{consumption}} \quad \text{(S40)}$$

The resulting EBT-BEQ were 31 $\mu g_{rotenone}/kg_{fish}$ or 2.2 $\mu g_{rotenone}/L_{milk}$ for the NOI assay, 52 $mg_{azoxystrobin}/kg_{fish}$ or 3.7 $mg_{azoxystrobin}/L_{milk}$ for the MMP assay, 187 ng_{T4}/kg_{fish} or 13.4 ng_{T4}/L_{milk} for TTR and 681 $\mu g_{perchlorate}/kg_{fish}$ or 48.7 $\mu g_{perchlorate}$ $/L_{milk}$ for NIS (Table S37).

These EBT were exceeded for all three types of fish for NOI (7 to 19 times), TTR (25 to 38 times) and NIS (12-13 times) but for MMP the BEQ_{bio} were just around or below the EBT-BEQ ((Figure S12F-H). The EBT of milk were just slightly exceeded in NOI (5-fold) and NIS (9-fold) and 32 to 37-fold in TTR (Figure S12I-J). It is possible that coextracted lipids induce PPARγ (therefore no EBT was derived) and these preliminary EBTs still need to be corrected for endogenous compounds. If only the mixture effects of the detected chemicals, expressed as BEQ_{chem} (Tables S38-42) are compared to the EBTs, then none of the fish and milk samples exceeds the EBT.

Text S18. Simplified toxicokinetic model to relate the food intake to blood concentrations

Treating the BEQ_{bio} of food as concentrations, one can apply very simple uptake models to estimate the blood concentrations and then compare the measured BEQ_{bio} in the blood extracts with the estimates from food uptake. This model is highly simplified and provides only an order-of-magnitude estimation, because the samples are not matched and do not represent the most contaminated food samples, but represent a European average scenario.

The bioanalytical equivalent dose (BED_{food} in units of $g_{reference\ compound}$/ person/day) is the sum over the amount of intake ($consumption_i$ in units of g_{food}/person/day) times the $BEQ_{i,food}$ of the food (in units of $g_{reference\ compound}/g_{food}$) in analogy to concentrations of single chemicals (eq. S41).

$$BED_{food} = \sum_{i=1}^{n} consumption_i \times BEQ_{i,food} \quad \text{(S41)}$$

We modelled two scenarios: (i) “complete absorption” as worst case, where we assumed 100% oral bioavailability, immediate distribution, no metabolism or excretion. Then the BEQ_{blood} was calculated with eq. S42.

$$BEQ_{complete\ absorption,blood} = \frac{BED_{food}}{V_{blood}} \quad \text{(S42)}$$

In scenario (ii) “steady state”, the steady state blood $BEQ_{ss,blood}$ was calculated with eq. S43, assuming oral bioavailability F of 100%, BED_{food} as the dose, and an estimated total body clearance CL (L/day) calculated from the volume of distribution V_d (L) and the elimination half-life $t_{1/2}$ (day) (eq. S43).

$$BEQ_{ss,blood} = \frac{F \times BED_{food}}{CL} \quad \text{(S43)}$$

$$CL = \frac{V_d \times \ln(2)}{t_{1/2}} \quad \text{(S44)}$$

Newborn babies were assumed to drink 300 mL of HBM per day in the first week and have a blood volume of approximately 300 mL. Note that we can only compare to umbilical cord serum as we have no experimental biomonitoring data for babies of any age, therefore we chose conditions for newborns for the calculations. The V_d is dependent on the hydrophobicity of the chemicals but as BED_{food} refers to a mixture composed of unknown chemicals, it is difficult to estimate the distribution of V_d. If we assume a V_d of 1

L/kg and a 4 kg baby (V_d of 4L), as well as a half-life of 1d, then the CL would be 2.77 L/day.

The resulting predicted BEQ_{food} (i.e., BEQ_{HBM}) and thereof derived $BEQ_{complete\ absorption,blood}$ and $BEQ_{ss,blood}$ (Table S38) agreed within a factor of ten with BEQ_{bio} of the umbilical cord serum sample S4 (Figure S13A) with a median % of BEQ explained by the model of 83% (95% CI 22 to 183% across all bioassays) with the complete absorption model and 9% (95% CI 4 to 23%) with the steady state model. Considering all assumptions and simplifications that had to be taken, this is a remarkably good agreement.

For adults, we used a blood volume V_{blood} of 6L and a V_d of 42 L for a 70 kg adult, which approximates the total body water, and a half-life of 1d. These assumptions would yield a CL of 61 L/day. We assumed a daily intake of 1L of DW + 1L of BW, 200 mL milk (100 mL OCM + 100 mL CCM) and 150 g fish (50g each of FFA, FFW, LFW). This is evidently only part of a typical food basket of an adult and consequently only 4.4% (95% CI 0 to 34%) were explained by the complete absorption model and 0.44% (95% CI 0 to 3.4%) by the steady state model (Table S38).

Text S19. Iceberg mixture modeling

The chemical BEQ_i in the heatmaps in Figure S14 are sorted from high to low concentration with the three hormones at the lower end and the mixture BEQ, the predicted BEQ_{chem} and the experimental BEQ_{mix} at the bottom. It is evident that the highest concentrated chemicals did not always have the highest BEQ_i. The on average most abundant MP was a dominant mixture driver for the serum samples in several assays, but the second most abundant Caf had a much lower contribution to most effects due to its low REP. BPA, which was the third most abundant chemical, also often strongly influenced the mixture effect. TCS had lower concentrations but was an important effect driver for MMP as it acts as uncoupler of oxidative phosphorylation but was also relatively active in NOI, TTR and NIS. The AR agonism of the hormone-free mixtures was dominated by Caf, BPA and BPAF.

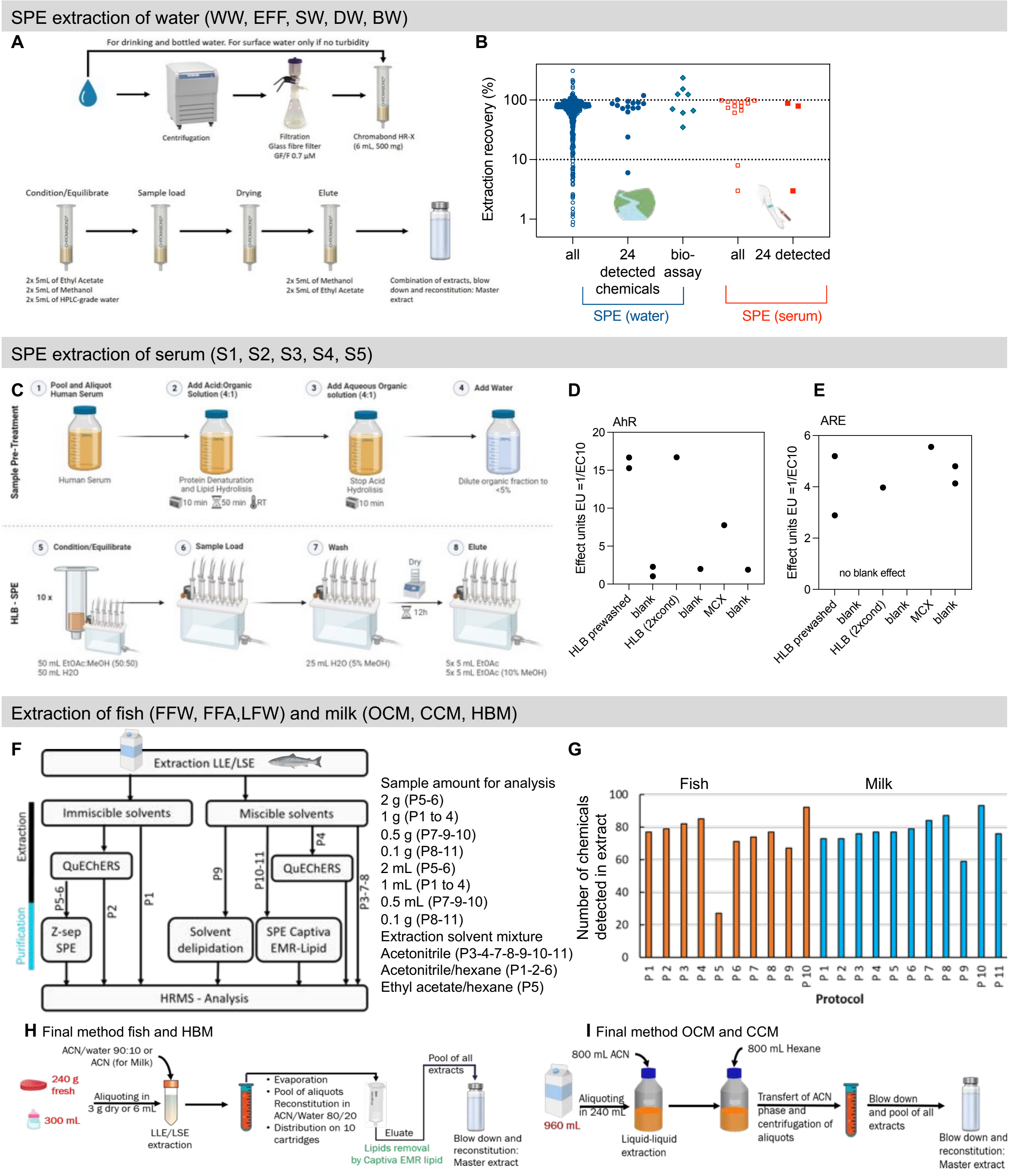

Figure S1. Recovery of chemicals from the investigated matrices.

A. SPE method for water extraction. B. Recovery after solid phase extraction of approximately 600 chemicals spiked to water[1] (empty blue circles) and the recovery of the 24 detected chemicals (solid blue circles) and bioassay recovery (teal diamonds) and SPE recovery of spiked chemicals in serum (empty red squares) and the recovery of the

24 detected chemicals (solid red squares) in this study. C. SPE method for serum extraction. D. Effect of different SPE materials (HLB and MCX) on effects in AhR. D. Effect of different SPE materials (HLB and MCX) on effects in ARE. F. Extraction method optimization for fish and milk. G. Number of chemicals recovered after extraction. H. Final extraction method for fish and HBM. I. Final extraction method for OCM and CCM.

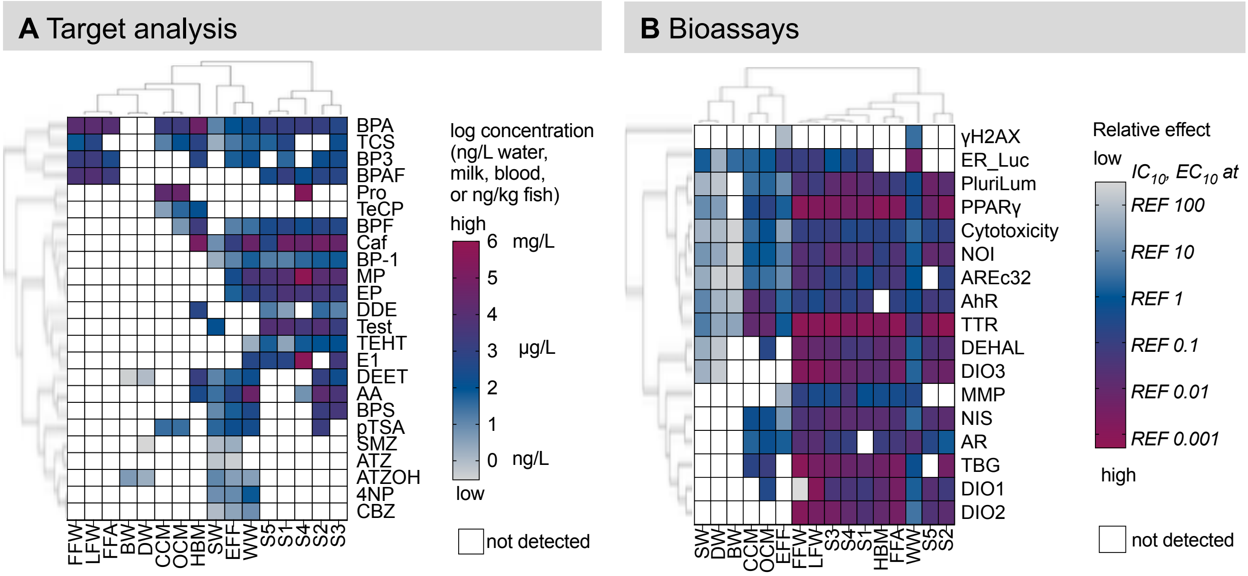

Figure S2. Hierarchical clustering of analytical and bioassay data with sample type. **(A)** Concentrations detected with target analysis (Data and abbreviations in Table S3). **(B)** Bioassays (Data and abbreviations in Table S5). Manhattan distance was used for both binary and continuous variables. Clustering was done using the Maximum Linkage approach.

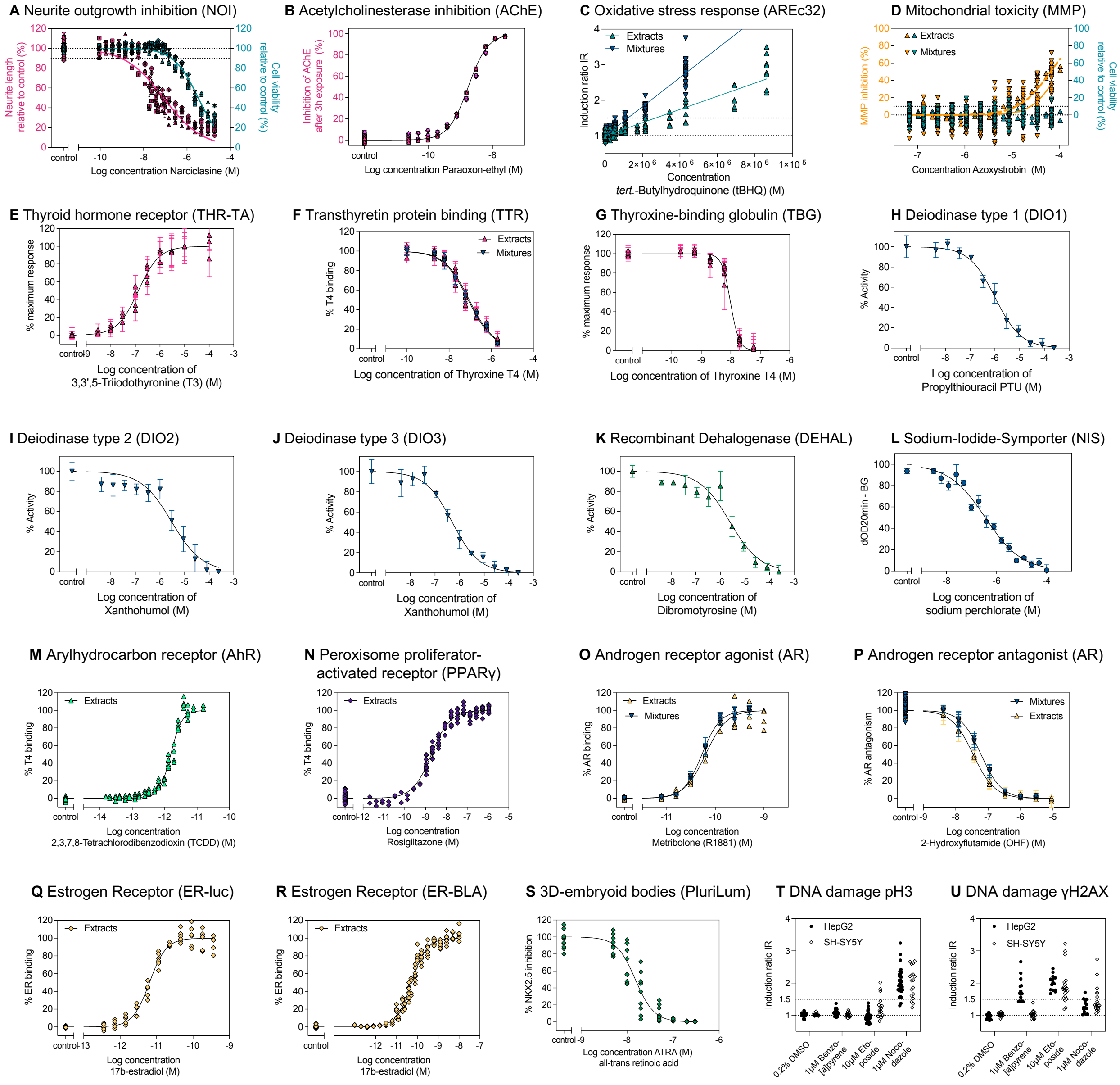

Figure S3. Quality control/quality assurance (QA/QC) of all bioassays.

Data are from Tables S7 to S26 (extracts) and Tables S29 to S42 (mixture experiments). The resulting QC parameters are in Table S5.

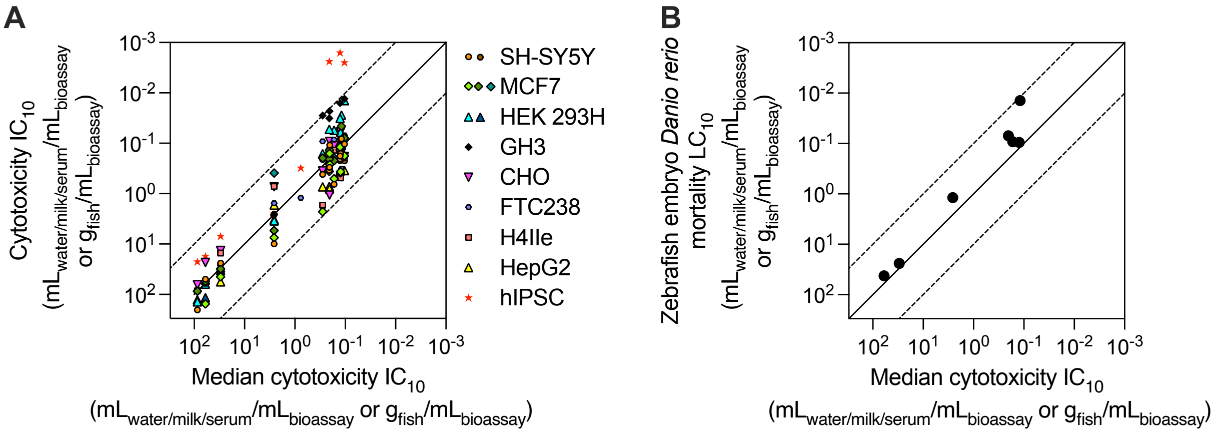

Figure S4. Comparison of cytotoxicity between all cell lines and mean cytotoxicity compared with zebrafish embryo mortality.

(A) Comparison of the 10% cytotoxicity inhibitory concentration IC_{10} of all individual cell lines with the median cytotoxicity IC_{10}. The different colors of the same symbol relate to the same parent cell line but a different reporter gene derivative.

(B) Comparison of the median cytotoxicity IC_{10} with the 10% lethal concentration towards the zebrafish embryo.

Data in Table S5. The full line is the 1:1 line, the dashed lines correspond to 1:10 and 10:1. Axes are inverted because a low IC_{10} relates to a high cytotoxicity.

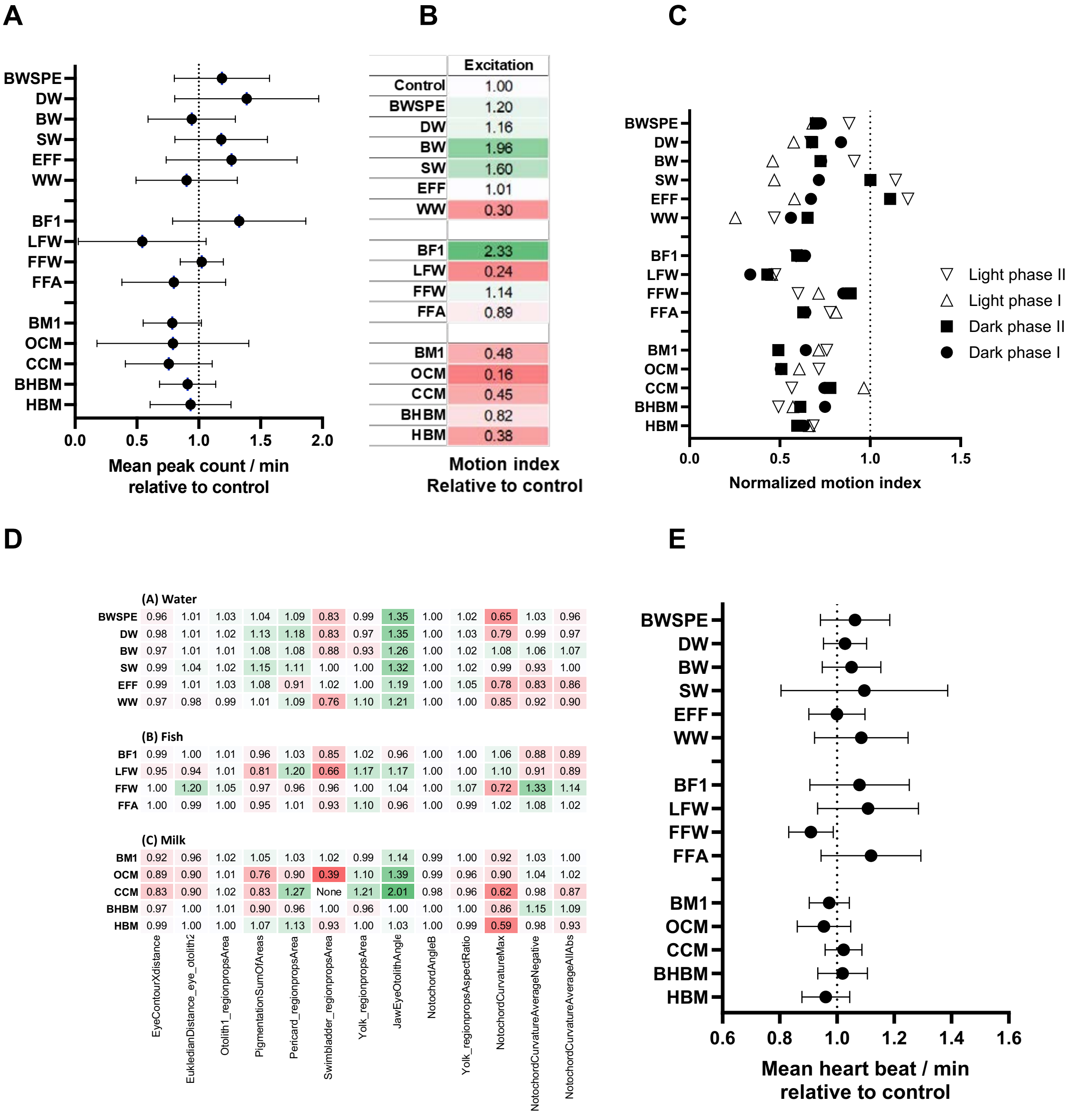

Figure S5. Behavioral effects of zebrafish larvae.

Effects of water, fish and milk samples on (A) spontaneous tail coiling (STC) after 24 h exposure and (B) photo motor response (PMR) after 30 h exposure to individual samples in zebrafish embryo at their IC_{10} level. Mean peak count was calculated relative to control per minute. Motion index was derived from pixel changes for excitation phase from 21 to 25 sec. (C) Locomotor response (LMR) in zebrafish embryo after 96 h exposure. Mean motion index was derived relative to control. (D) Effects on morphology in hatched

zebrafish after 96 h exposure. Mean of individual measures was derived relative to control. (E) Effects on heart rate in hatched zebrafish after 96 h exposure. Mean heart rate (beats per minute) was normalized relative to control, error bars are standard deviation of mean.

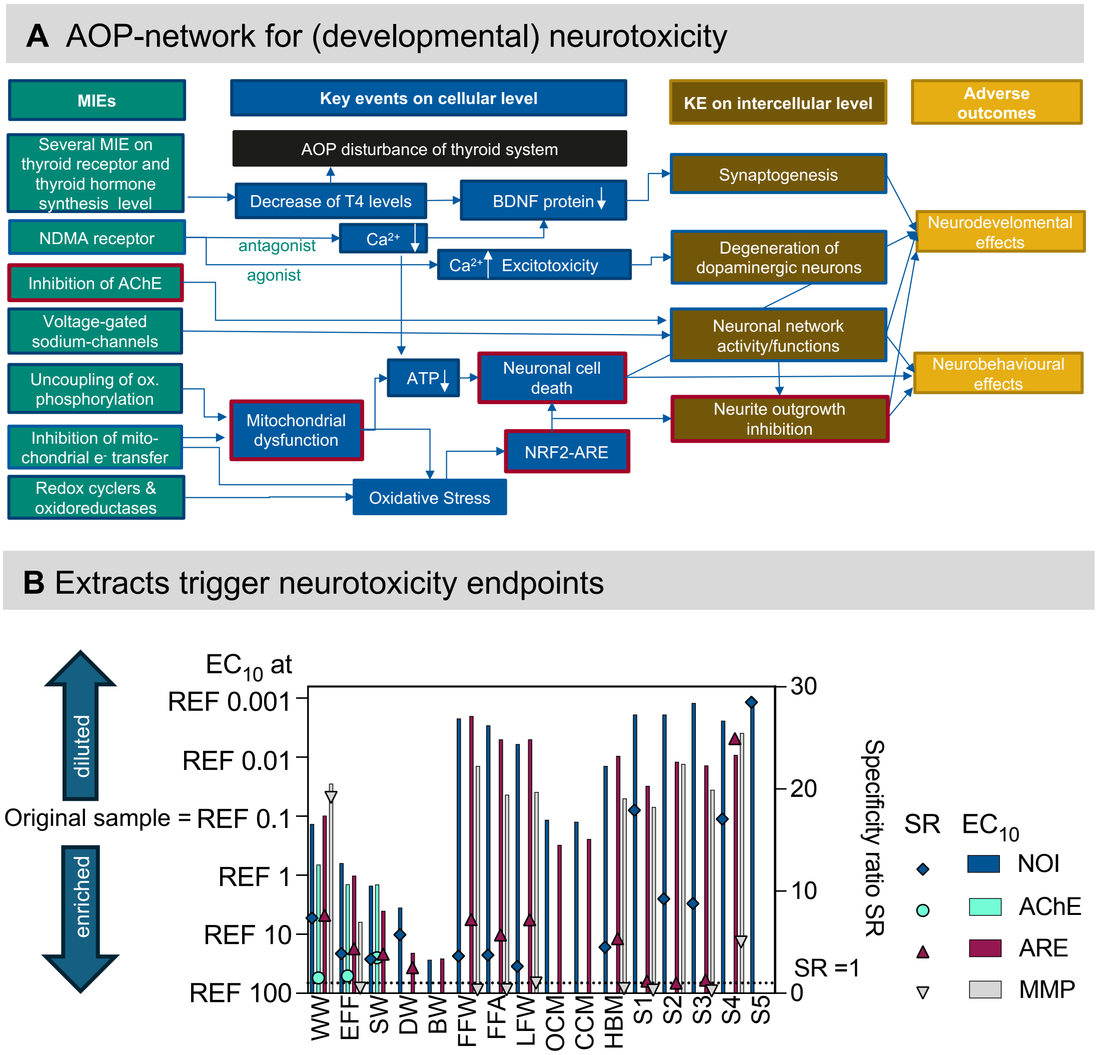

Figure S6. Developmental neurotoxicity.

(A) Simplified Adverse Outcome Pathway (AOP) network describing various neurotoxicity outcomes related to developmental neurotoxicity. Framed in red are Molecular Initiating Events (MIE) and Key Events (KE) where screening-level in vitro bioassays are included in this study.

(B) Left y-axis: A low EC_{10} or $EC_{IR1.5}$ refers to high effect. The specificity of the effect in relation to the cytotoxicity IC_{10} (specificity ratio SR = IC_{10}/EC_{10}) is depicted with symbols on the right Y-axis. The dotted line indicates SR=1.

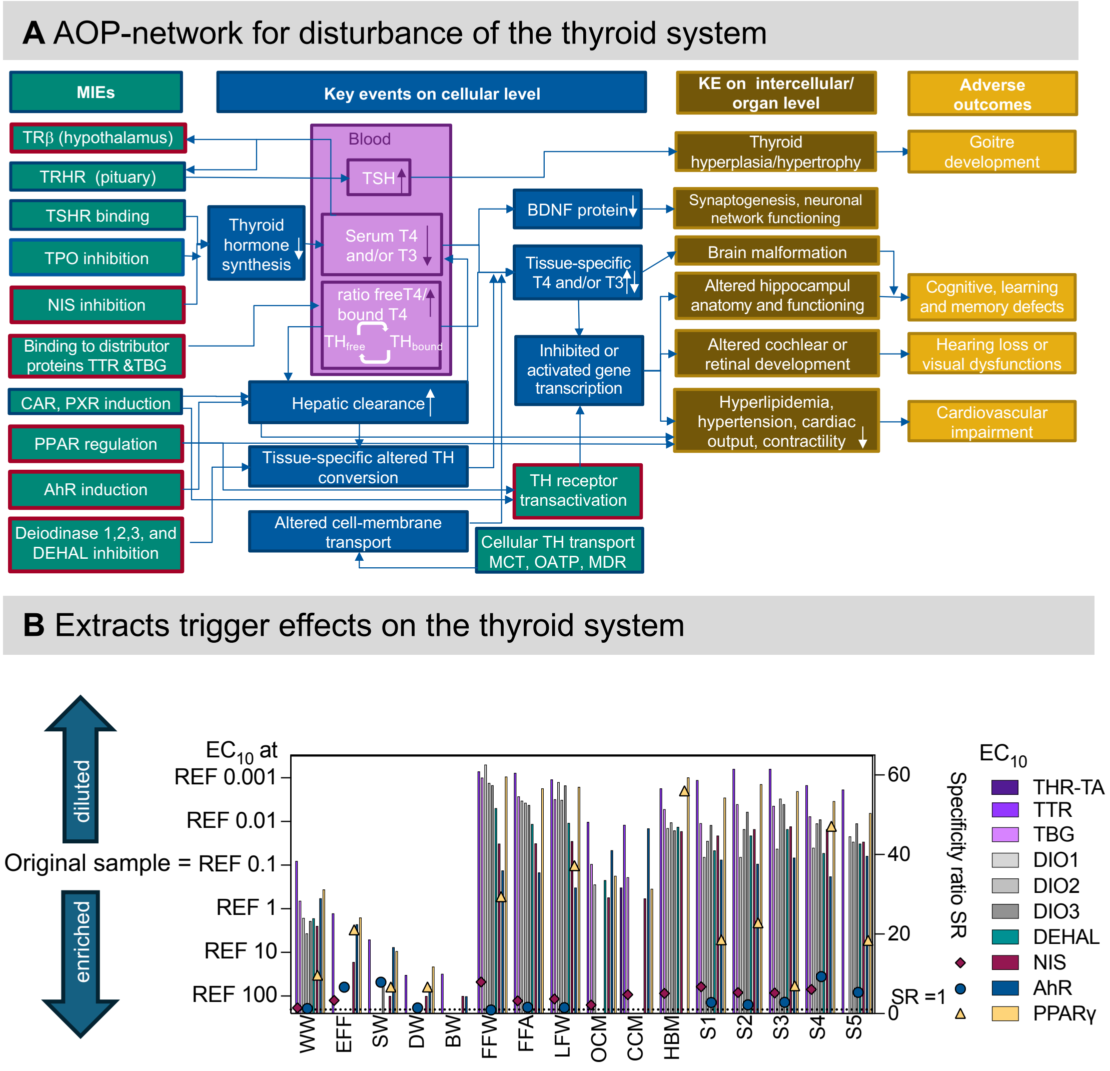

Figure S7. Thyroid disorders.

(A) Thyroid hormone system-related AOP network. Framed in red are MIEs and KEs where *in vitro* bioassays are applied. Figure modified from Noyes et al.[74] with some MIEs omitted for brevity.

(B) Left y-axis: EC_{10} or $EC_{IR1.5}$ in all assays associated with the thyroid hormone system-related AOP network. Right y-axis: specificity ratio ($SR = IC_{10}/EC_{10}$) for the cellular assays.

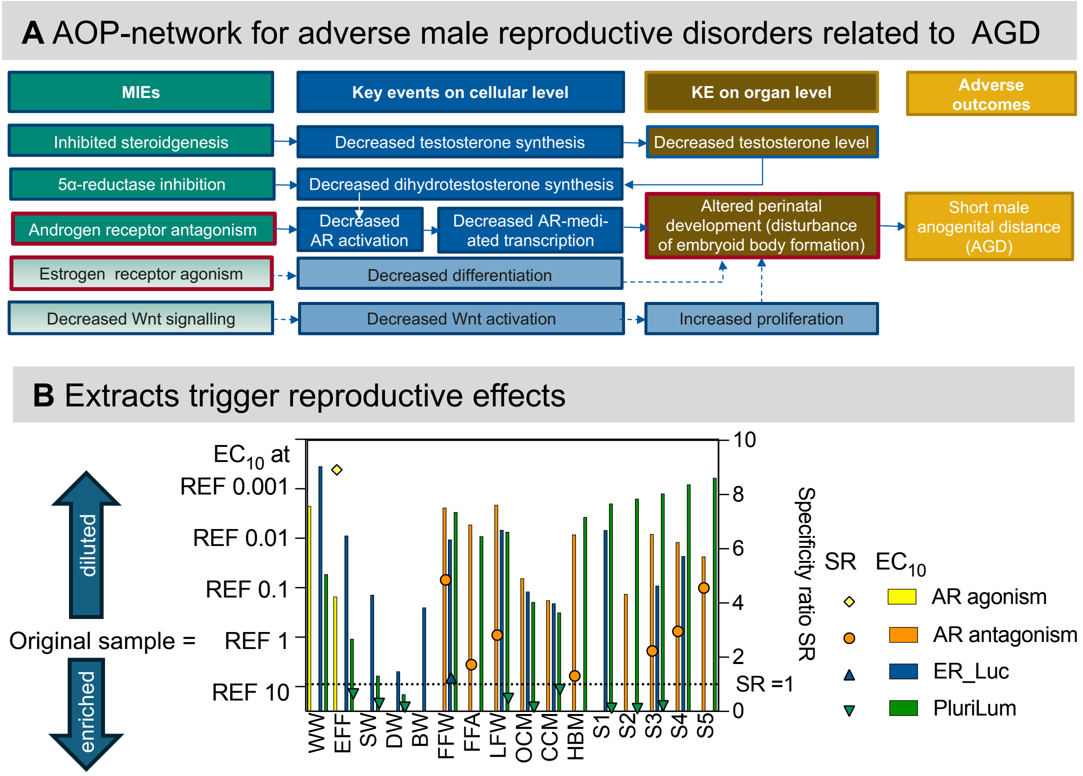

Figure S8. Reproductive disorders.

(A) Simplified AOP network for reproductive effects, focusing on adverse male disorders related to shortened anogenital distance (AGD).

(B) Left y-axis: EC_{10} in all reproduction assays. Right y-axis: specificity ratio ($SR = IC_{10}/EC_{10}$) for the cellular assays.

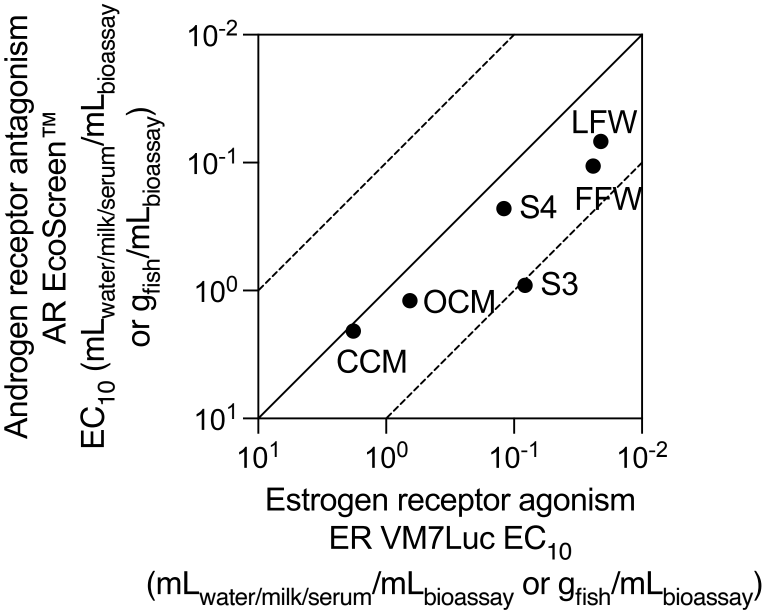

Figure S9. Association of estrogen receptor agonism and androgen receptor antagonism.

Comparison of the 10% effect concentration EC_{10} of estrogen receptor (ER) agonism with EC_{10} of androgen receptor (AR) antagonism. Date from Table S21 and S22. R^2 of linear regression of log-transformed data = 0.800.

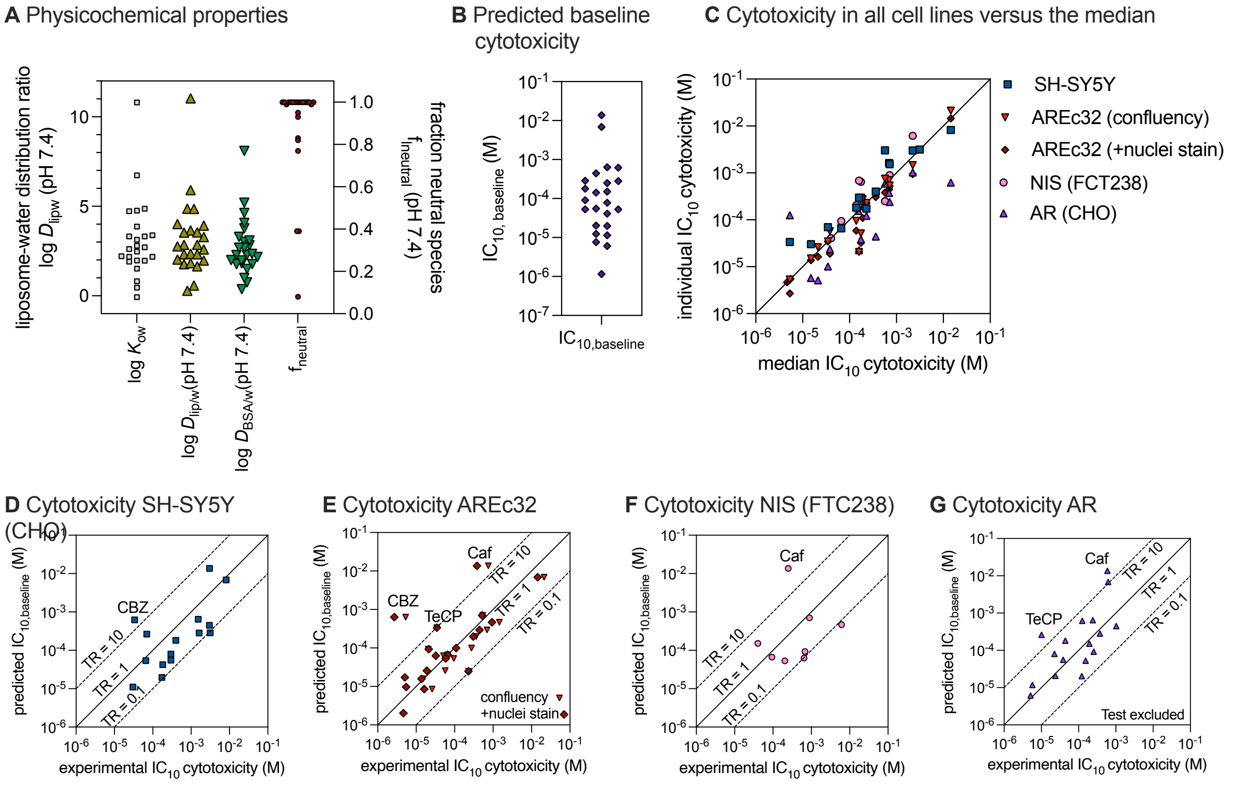

Figure S10. Comparison of individual chemical's cytotoxicity with their predicted baseline toxicity.

(A) Range of physicochemical properties, octanol-water partition K_{ow}, ionization-corrected distribution ratios between biomembranes (liposomes) (lip) and water $D_{lip/w}$ ionization-corrected distribution ratios between bovine serum albumin (BSA) and water $D_{BSA/w}$, fraction of neutral species $f_{neutral}$ (Table S26). **(B)** distribution of predicted baseline cytotoxicity $IC_{10,baseline}$ (mean of all cell lines) (Table S26). **(C)** Comparison between median experimental cytotoxicity IC_{10} and individual IC_{10} for all cell lines (CHO = Chinese hamster ovary) (Table S29-S35). **(D)** Comparison between experimental cytotoxicity IC_{10} and predicted baseline $IC_{10,baseline}$ for SH-SY5Y cells (Table S29). **(E)** Comparison between experimental cytotoxicity IC_{10} and predicted baseline $IC_{10,baseline}$ for AREc32 cells. The two different cytotoxicity quantification methods of confluence (i.e., growth, (Table S30)) and cell viability with nuclei staining (Table S31) agreed well with each other. **(F)** Comparison between experimental cytotoxicity IC_{10} and predicted baseline $IC_{10,baseline}$ for NIS assay's FTC238 cells (Table S32). **(G)** Comparison between experimental cytotoxicity IC_{10} and predicted baseline $IC_{10,baseline}$ for the CHO cells of the AR assay

(Table S33). The toxic ratio (TR) of 1 is depicted with a solid line and the range 0.1<TR<10, which is the range of baseline toxicity with dotted lines.

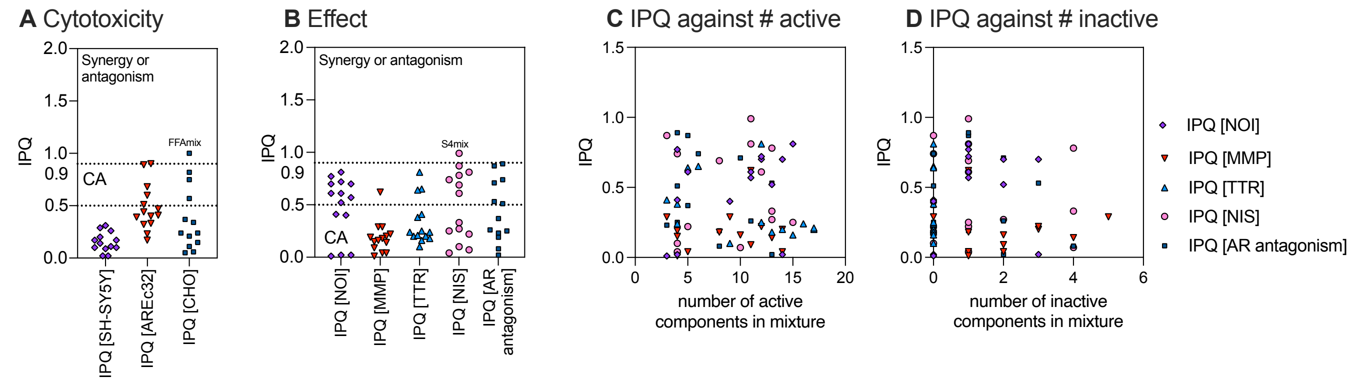

Figure S11. Index on prediction quality IPQ.

(A) IPQ (eq. 29) for the cytotoxicity IC_{10}.

(B) IPQ (eq. 29) for the effect endpoints EC_{10}.

(C) The IPQ is not associated to the number of active chemicals in the mixture.

(D) The IPQ is not associated to the number of inactive chemicals in the mixture.

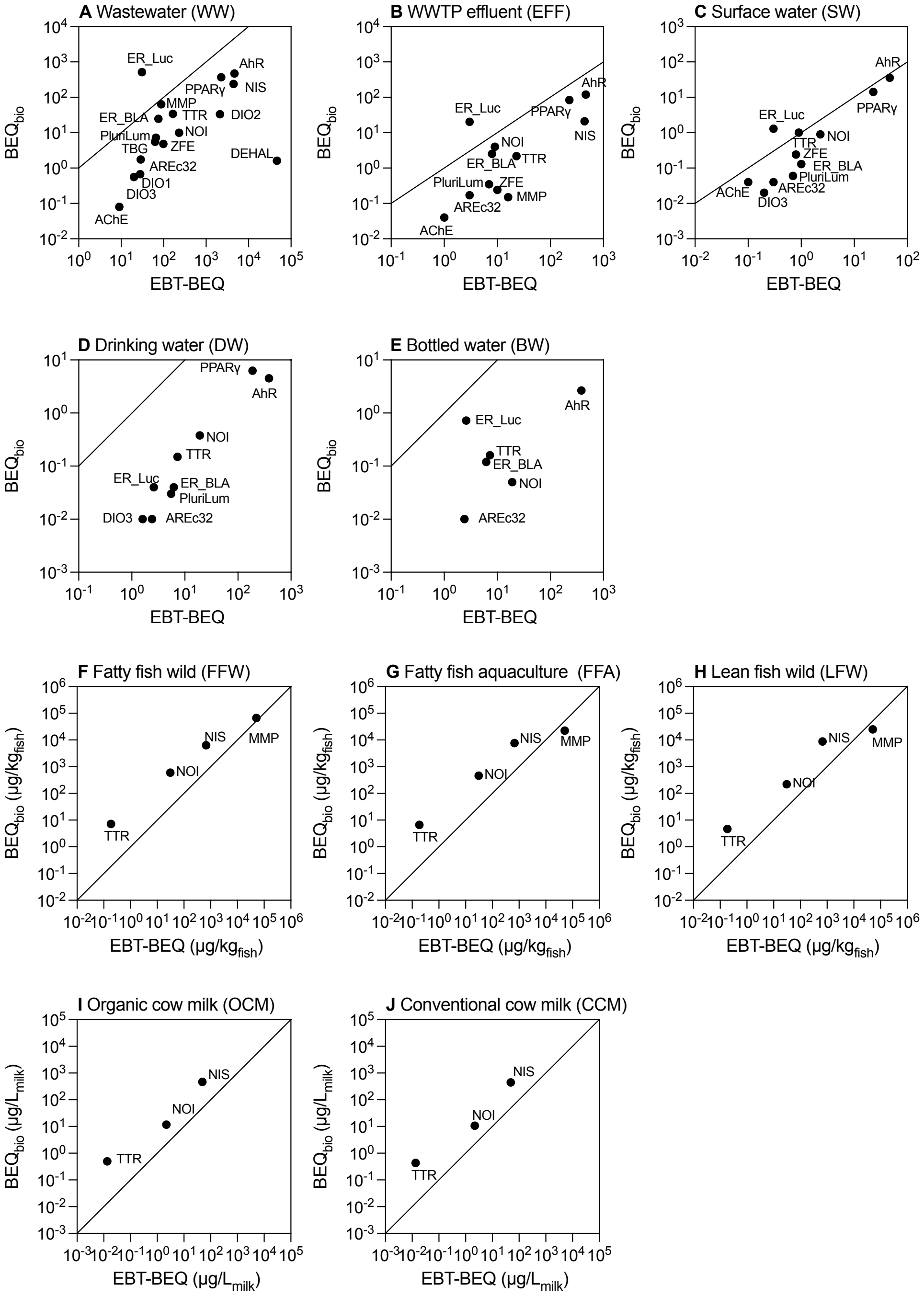

Figure S12. Comparison of effect data expressed as bioanalytical equivalent concentrations BEQ_{bio} with effect-based trigger values EBT-BEQ.

(A) WW, (B) EFF, (C) SW, (D) DW, (E) BW, (F) FFW, (G) FFA, (H) LWF, (I) OCM, (J) CCM. BEQ and EBT-BEQ from Table S37.

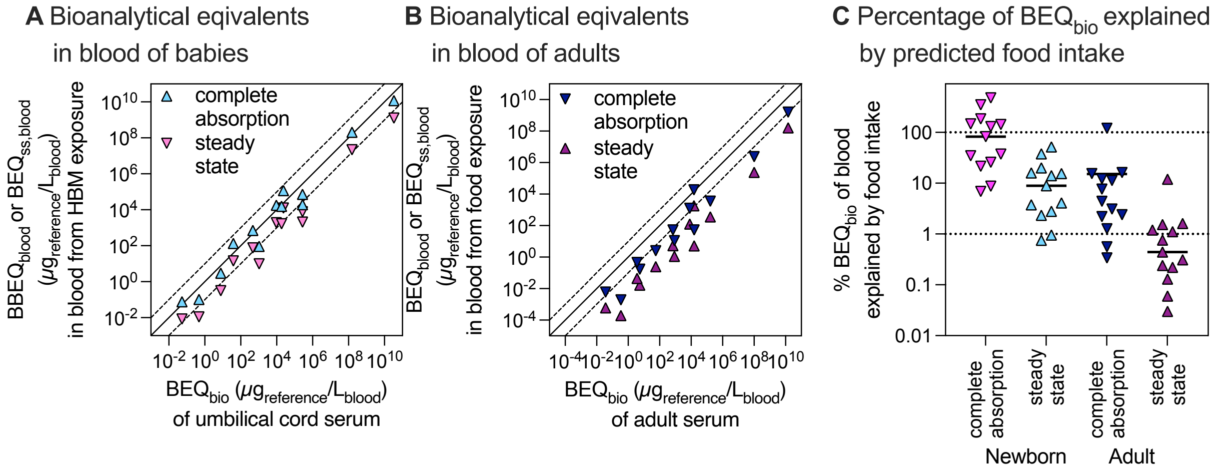

Figure S13. Comparison of bioanalytical equivalent concentrations BEQ in blood directly measured and estimated from food intake.

(A) Comparison of BEQ_{bio} of umbilical cord serum (sample S4, Table S5, blank-corrected data, converted to BEQ in Table S37) with BEQ_{bio} in blood of babies estimated from intake of human breast milk (HMB) under assumptions of complete absorption or a simple steady state model with a clearance of 2.77L/d (Table S37).

(B) Comparison of mean BEQ_{bio} of adult serum extracts (samples S1-S3 and S5, Table S5, blank-corrected data, converted to BEQ in Table S37) with BEQ_{bio} in blood of adults estimated from intake of drinking water, milk and fish under assumptions of complete absorption or a simple steady state model with a clearance of 60.6 L/d (Table S37).

(C) Distribution of the percentage of BEQ_{bio} explained by food intake (Table S37).

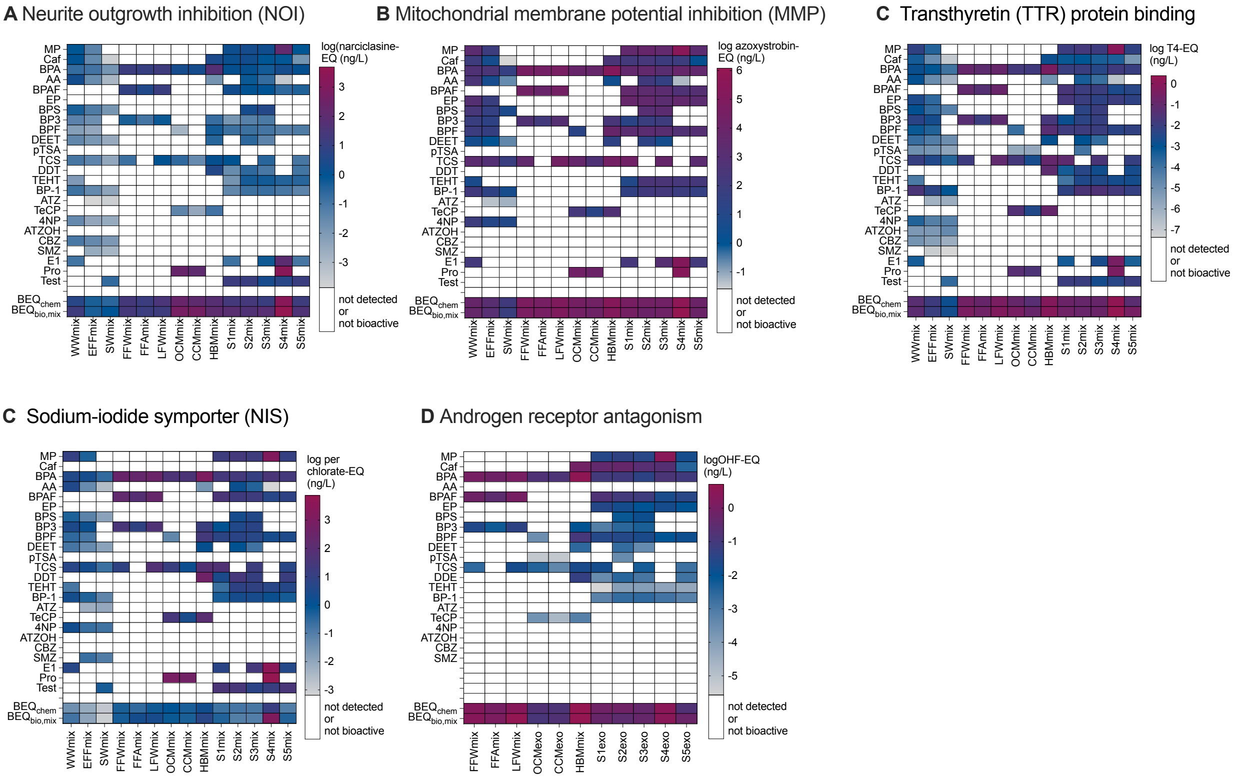

Figure S14. Contribution of individual chemicals' bioanalytical equivalent concentrations BEQ_i to the predicted mixture BEQ_{chem} and measured $BEQ_{bio,mix}$ of the designed mixture.

(A) Neurite outgrowth inhibition (NOI), reference chemical rotenone (Table S39).

(B) Mitochondrial membrane potential inhibition (MMP), reference chemical azoxystrobin (Table S40).

(C) Transthyretin (TTR) protein binding, reference chemical thyroxine (T4) (Table S41).

(D) Sodium-iodide symporter (NIS), reference chemical perchlorate (Table S42).

(E) Androgen receptor antagonism, reference chemical hydroxyflutamide (OHF) (Table S43).

References

(1) Neale, P. A.; Brack, W.; Ait-Aissa, S.; Busch, W.; Hollender, J.; Krauss, M.; Maillot-Maréchal, E.; Munz, N. A.; Schlichting, R.; Schulze, T.; Vogler, B.; Escher, B. Solid-phase extraction as sample preparation of water samples for cell-based and other *in vitro* bioassays. *Environ. Sci. Process. Impacts* **2018**, *20*, 493–504. DOI: DOI: 10.1039/C7EM00555E
(2) Schulze, T.; Neale, P. A.; Ahlheim, J.; Beckers, L.-M.; König, M.; Krüger, J.; Petre, M.; Piotrowska, A.; Schlichting, R.; Schmidt, S.; Krauss, M.; Escher, B. I. A guidance for

the enrichment of micropollutants from wastewater by solid-phase extraction before bioanalytical assessment. *Environ. Sci Europe* **2024**, *36* (1), 165. DOI: 10.1186/s12302-024-00990-x
(3) Simon, E.; Bytingsvik, J.; Jonker, W.; Leonards, P. E. G.; de Boer, J.; Jenssen, B. M.; Lie, E.; Aars, J.; Hamers, T.; Lamoree, M. H. Blood plasma sample preparation method for the assessment of thyroid hormone-disrupting potency in effect-directed analysis. *Environ. Sci. Technol.* **2011**, *45* (18), 7936–7944. DOI: 10.1021/es2016389
(4) Pourchet, M.; Narduzzi, L.; Jean, A.; Guiffard, I.; Bichon, E.; Cariou, R.; Guitton, Y.; Hutinet, S.; Vlaanderen, J.; Meijer, J.; Le Bizec, B.; Antignac, J.-P. Non-targeted screening methodology to characterise human internal chemical exposure: Application to halogenated compounds in human milk. *Talanta* **2021**, *225*, 121979. DOI: 10.1016/j.talanta.2020.121979
(5) Motteau, S.; Dervilly, G.; Cariou, R.; Margalef, M.; Lamoree, M.; Hamers, T.; König, M.; Escher, B. I.; Vinggaard, A. M.; Rørbye, C.; Le Bizec, B.; Antignac, J.-P. Determination of Chemical Mixtures in Environmental, Food, and Human Samples Using High-Resolution Mass Spectrometry-Based Suspect Screening Approaches. *Environ. Sci. Technol.* **2025**, *59* (39), 21265–21277. DOI: 10.1021/acs.est.4c12608
(6) Pastor-Barriuso, R.; Fernández Mariana, F.; Castaño-Vinyals, G.; Whelan, D.; Pérez-Gómez, B.; Llorca, J.; Villanueva Cristina, M.; Guevara, M.; Molina-Molina, J.-M.; Artacho-Cordón, F.; Barriuso-Lapresa, L.; Tusquets, I.; Dierssen-Sotos, T.; Aragonés, N.; Olea, N.; Kogevinas, M.; Pollán, M. Total Effective Xenoestrogen Burden in Serum Samples and Risk for Breast Cancer in a Population-Based Multicase–Control Study in Spain. *Environ. Health Perspect.* **2016**, *124* (10), 1575–1582. DOI: 10.1289/EHP157
(7) Schenck, F. J.; Hobbs, J. E. Evaluation of the Quick, Easy, Cheap, Effective, Rugged, and Safe (QuEChERS) Approach to Pesticide Residue Analysis. *Bull. Environ. Contam. Toxicol.* **2004**, *73* (1), 24–30. DOI: 10.1007/s00128-004-0388-y
(8) Reiter, E. B.; Jahnke, A.; Konig, M.; Siebert, U.; Escher, B. I. Influence of Co-Dosed Lipids from Biota Extracts on the Availability of Chemicals in In Vitro Cell-Based Bioassays. *Environ. Sci. Technol.* **2020**, *54* (7), 4240–4247. DOI: 10.1021/acs.est.9b07850
(9) Ortmann, J.; Altenburger, R.; Scholz, S.; Luckenbach, T. Photomotor Response Data Analysis Approach to Assess Chemical Neurotoxicity with the Zebrafish Embryo. *Altex-Alternat. Animal Exp.* **2022**, *39* (1), 82–94. DOI: 10.14573/altex.2004021
(10) Teixido, E.; Kiessling, T. R.; Kluver, N.; Scholz, S. Grouping of chemicals into mode of action classes by automated effect pattern analysis using the zebrafish embryo toxicity test. *Arch. Toxicol.* **2022**, *96* (5), 1353–1369. DOI: 10.1007/s00204-022-03253-x
(11) Teixido, E.; Kiessling, T. R.; Krupp, E.; Quevedo, C.; Muriana, A.; Scholz, S. Automated Morphological Feature Assessment for Zebrafish Embryo Developmental Toxicity Screens. *Tox. Sci.* **2019**, *167* (2), 438–449. DOI: 10.1093/toxsci/kfy250
(12) Masjosthusmann, S.; Blum, J.; Bartmann, K.; Dolde, X.; Holzer, A.-K.; Stürzl, L.-C.; Keßel, E. H.; Förster, N.; Dönmez, A.; Klose, J.; Pahl, M.; Waldmann, T.; Bendt, F.; Kisitu, J.; Suciu, I.; Hübenthal, U.; Mosig, A.; Leist, M.; Fritsche, E. Establishment of an a priori protocol for the implementation and interpretation of an in-vitro testing battery for the assessment of developmental neurotoxicity. *EFSA Supporting Publications* **2020**, *17* (10), 1938E. DOI: 10.2903/sp.efsa.2020.EN-1938

(13) Ellman, G. L.; Courtney, K. D.; Andres jr, V.; Featherstone, R. M. A new and rapid colorimetric determination of acetylcholinesterase activity. *Biochem. Pharmacol.* **1961**, *7* (2), 88–95. DOI: Doi: 10.1016/0006-2952(61)90145-9
(14) Wang, X. J.; Hayes, J. D.; Wolf, C. R. Generation of a stable antioxidant response element–driven reporter gene cell line and its use to show redox-dependent activation of Nrf2 by cancer chemotherapeutic agents. *Cancer Res.* **2006**, *66* (22), 10983–10994. DOI: 10.1158/0008-5472.can-06-2298
(15) Escher, B. I.; Dutt, M.; Maylin, E.; Tang, J. Y. M.; Toze, S.; Wolf, C. R.; Lang, M. Water quality assessment using the AREc32 reporter gene assay indicative of the oxidative stress response pathway. *J. Environ. Monitor.* **2012**, *14* (11), 2877–2885. DOI: 10.1039/c2em30506b
(16) Neale, P. A.; Altenburger, R.; Ait-Aissa, S.; Brion, F.; Busch, W.; de Aragão Umbuzeiro, G.; Denison, M. S.; Du Pasquier, D.; Hilscherova, K.; Hollert, H.; Morales, D. A.; Novac, J.; Schlichting, R.; Seiler, T.-B.; Serra, H.; Shao, Y.; Tindall, A. J.; Tollefsen, K. E.; Williams, T. D.; Escher, B. I. Development of a bioanalytical test battery for water quality monitoring: Fingerprinting identified micropollutants and their contribution to effects in surface water. *Water Res.* **2017**, *123*, 734–750. DOI: 10.1016/j.watres.2017.07.016
(17) Escher, B. I.; Glauch, L.; Konig, M.; Mayer, P.; Schlichting, R. Baseline Toxicity and Volatility Cutoff in Reporter Gene Assays Used for High-Throughput Screening. *Chem. Res. Toxicol.* **2019**, *32* (8), 1646–1655. DOI: 10.1021/acs.chemrestox.9b00182
(18) Lee, J.; König, M.; Braun, G.; Escher, B. I. Water Quality Monitoring with the Multiplexed Assay MitoOxTox for Mitochondrial Toxicity, Oxidative Stress Response, and Cytotoxicity in AREc32 Cells. *Environ. Sci. Technol.* **2024**, *58* (13), 5716–5726. DOI: 10.1021/acs.est.3c09844
(19) Freitas, J.; Cano, P.; Craig-Veit, C.; Goodson, M. L.; David Furlow, J.; Murk, A. J. Detection of thyroid hormone receptor disruptors by a novel stable *in vitro* reporter gene assay. *Toxicol. in Vitro* **2011**, *25* (1), 257–266. DOI: 10.1016/j.tiv.2010.08.013
(20) O'Brien, J.; Wilson, I.; Orton, T.; Pognan, F. Investigation of the Alamar Blue (resazurin) fluorescent dye for the assessment of mammalian cell cytotoxicity. *European Journal of Biochemistry* **2000**, *267* (17), 5421–5426, https://doi.org/10.1046/j.1432-1327.2000.01606.x. DOI: 10.1046/j.1432-1327.2000.01606.x
(21) Schriks, M.; Vrabie, C. M.; Gutleb, A. C.; Faassen, E. J.; Rietjens, I. M. C. M.; Murk, A. J. T-screen to quantify functional potentiating, antagonistic and thyroid hormone-like activities of poly halogenated aromatic hydrocarbons (PHAHs). *Toxicol. in Vitro* **2006**, *20* (4), 490–498. DOI: DOI: 10.1016/j.tiv.2005.09.001
(22) Murk, A. J.; Leonards, P. E. G.; van Hattum, B.; Luit, R.; van der Weiden, M. E. J.; Smit, M. Application of biomarkers for exposure and effect of polyhalogenated aromatic hydrocarbons in naturally exposed European otters (Lutra lutra). *Environ. Toxicol. Pharmacol.* **1998**, *6* (2), 91–102. DOI: 10.1016/S1382-6689(98)00026-X
(23) Shen, Y.; Bovee, T. F. H.; Molenaar, D.; Weide, Y.; Nolles, A.; Braucic Mitrovic, C.; van Leeuwen, S. P. J.; Louisse, J.; Hamers, T. Optimized methods for measuring competitive binding of chemical substances to thyroid hormone distributor proteins transthyretin and thyroxine binding globulin. *Arch. Toxicol.* **2024**, *98* (11), 3797–3809. DOI: 10.1007/s00204-024-03842-y
(24) Renko, K.; Schache, S.; Hoefig, C. S.; Welsink, T.; Schwiebert, C.; Braun, D.; Becker, N. P.; Kohrle, J.; Schomburg, L. An Improved Nonradioactive Screening

Method Identifies Genistein and Xanthohumol as Potent Inhibitors of Iodothyronine Deiodinases. *Thyroid* **2015**, *25* (8), 962–968. DOI: 10.1089/thy.2015.0058
(25) Renko, K.; Hoefig, C. S.; Hiller, F.; Schomburg, L.; Köhrle, J. Identification of iopanoic acid as substrate of type 1 deiodinase by a novel nonradioactive iodide-release assay. *Endocrinology* **2012**, *153* (5), 2506–2513. DOI: 10.1210/en.2011-1863
(26) Renko, K.; Hoefig, C. S.; Dupuy, C.; Harder, L.; Schwiebert, C.; Kohrle, J.; Schomburg, L. A Nonradioactive DEHAL Assay for Testing Substrates, Inhibitors, and Monitoring Endogenous Activity. *Endocrinology* **2016**, *157* (12), 4516–4525. DOI: 10.1210/en.2016-1549
(27) Eleftheriadou, A.-M.; Mehl, S.; Renko, K.; Kasim, R. H.; Schaefer, J.-A.; Minich, W. B.; Schomburg, L. Re-visiting autoimmunity to sodium-iodide symporter and pendrin in thyroid disease. *European Journal of Endocrinology* **2020**, *183* (6), 571–580. DOI: 10.1530/EJE-20-0566
(28) OECD. *Test Guideline No. 458: Stably Transfected Human Androgen Receptor Transcriptional Activation Assay for Detection of Androgenic Agonist and Antagonist Activity of Chemicals, OECD Guidelines for the Testing of Chemicals, Section 4, OECD Publishing, Paris,* ; Environmental Directorate, Organisation for Economic Co-operation and Development, Paris, France, 2013. DOI: 10.1787/9789264264366-en (accessed 2025/04/05).
(29) König, M.; Escher, B. I.; Neale, P. A.; Krauss, M.; Hilscherová, K.; Novák, J.; Teodorović, I.; Schulze, T.; Seidensticker, S.; Kamal Hashmi, M. A.; Ahlheim, J.; Brack, W. Impact of untreated wastewater on a major European river evaluated with a combination of *in vitro* bioassays and chemical analysis. *Environ. Pollut.* **2017**, *220*, 1220–1230. DOI: 10.1016/j.envpol.2016.11.011
(30) Brennan, J. C.; He, G.; Tsutsumi, T.; Zhao, J.; Wirth, E.; Fulton, M. H.; Denison, M. S. Development of Species-Specific Ah Receptor-Responsive Third Generation CALUX Cell Lines with Enhanced Responsiveness and Improved Detection Limits. *Environ. Sci. Technol.* **2015**, *49* (19), 11903–11912. DOI: 10.1021/acs.est.5b02906
(31) OECD. *Test guideline no. 455. Performance-Based Test Guideline for Stably Transfected Transactivation In Vitro Assays to Detect Estrogen Receptor Agonists and Antagonist*; Environmental Directorate, Organisation for Economic Co-operation and Development, Paris, France, 2015.
(32) Lauschke, K.; Treschow, A. F.; Rasmussen, M. A.; Davidsen, N.; Holst, B.; Emneus, J.; Taxvig, C.; Vinggaard, A. M. Creating a human-induced pluripotent stem cell-based NKX2.5 reporter gene assay for developmental toxicity testing. *Arch. Toxicol.* **2021**, *95* (5), 1659–1670. DOI: 10.1007/s00204-021-03018-y
(33) Treschow, A. F.; Vinggaard, A. M.; Valente, M. J. Standardization and optimization of the hiPSC-based PluriLum assay for detection of embryonic and developmental toxicants. *Arch. Toxicol.* **2024**, *98* (12), 4107–4116. DOI: 10.1007/s00204-024-03870-8
(34) Graillot, V.; Takakura, N.; Le Hegarat, L.; Fessard, V.; Audebert, M.; Cravedi, J. P. Genotoxicity of pesticide mixtures present in the diet of the French population. *Environ. Mol. Mutagen.* **2012**, *53* (3), 173–184. DOI: 10.1002/em.21676
(35) Graillot, V.; Tomasetig, F.; Cravedi, J. P.; Audebert, M. Evidence of the in vitro genotoxicity of methyl-pyrazole pesticides in human cells. *Mutat. Res.* **2012**, *748* (1-2), 8–16. DOI: 10.1016/j.mrgentox.2012.05.014

(36) Khoury, L.; Zalko, D.; Audebert, M. Validation of High-Throughput Genotoxicity Assay Screening Using gamma H2AX In-Cell Western Assay on HepG2 Cells. *Environ. Mol. Mutagen.* **2013**, *54* (9), 737–746. DOI: 10.1002/em.21817
(37) Khoury, L.; Zalko, D.; Audebert, M. Evaluation of four human cell lines with distinct biotransformation properties for genotoxic screening. *Mutagenesis* **2016**, *31* (1), 83–96. DOI: 10.1093/mutage/gev058
(38) Khoury, L.; Zalko, D.; Audebert, M. Evaluation of the genotoxic potential of apoptosis inducers with the gamma H2AX assay in human cells. *Mutat. Res.* **2020**, *852*, 503165. DOI: 10.1016/j.mrgentox.2020.503165
(39) Escher, B.; Neale, P. A.; Villeneuve, D. The advantages of linear concentration-response curves for *in vitro* bioassays with environmental samples. *Environ. Toxicol. Chem.* **2018**, *37* (9), 2273–2280. DOI: 10.1002/etc.4178
(40) Judson, R.; Houck, K.; Martin, M.; Richard, A. M.; Knudsen, T. B.; Shah, I.; Little, S.; Wambaugh, J.; Setzer, R. W.; Kothya, P.; Phuong, J.; Filer, D.; Smith, D.; Reif, D.; Rotroff, D.; Kleinstreuer, N.; Sipes, N.; Xia, M. H.; Huang, R. L.; Crofton, K.; Thomas, R. S. Analysis of the Effects of Cell Stress and Cytotoxicity on In Vitro Assay Activity Across a Diverse Chemical and Assay Space. *Tox. Sci.* **2016**, *152* (2), 323–339. DOI: 10.1093/toxsci/kfw092
(41) Fay, K. A.; Villeneuve, D. L.; Swintek, J.; Edwards, S. W.; Nelms, M. D.; Blackwell, B. R.; Ankley, G. T. Differentiating Pathway-Specific From Nonspecific Effects in High-Throughput Toxicity Data: A Foundation for Prioritizing Adverse Outcome Pathway Development. *Tox. Sci.* **2018**, *163* (2), 500–515. DOI: 10.1093/toxsci/kfy049
(42) Escher, B. I.; Henneberger, L.; Schlichting, R.; Fischer, F. C. Cytotoxicity burst or baseline toxicity? Differentiating specific from nonspecific effects in reporter gene assays *Environ Health Perspect* **2020**, *128* (7), 077007. DOI: 10.1289/EHP6664
(43) Fellows, M. D.; O'Donovan, M. R.; Lorge, E.; Kirkland, D. Comparison of different methods for an accurate assessment of cytotoxicity in the in vitro micronucleus test II: Practical aspects with toxic agents. *Mutat. Res.* **2008**, *655* (1-2), 4–21. DOI: 10.1016/j.mrgentox.2008.06.004
(44) Dertinger, S. D.; Kraynak, A. R.; Wheeldon, R. P.; Bernacki, D. T.; Bryce, S. M.; Hall, N.; Bemis, J. C.; Galloway, S. M.; Escobar, P. A.; Johnson, G. E. Predictions of genotoxic potential, mode of action, molecular targets, and potency via a tiered multiflow® assay data analysis strategy. *Environ. Mol. Mutagen.* **2019**, *60* (6), 513–533, 10.1002/em.22274. DOI: 10.1002/em.22274
(45) Lee, J.; Braun, G.; Henneberger, L.; König, M.; Schlichting, R.; Scholz, S.; Escher, B. I. Critical Membrane Concentration and Mass-Balance Model to Identify Baseline Cytotoxicity of Hydrophobic and Ionizable Organic Chemicals in Mammalian Cell Lines. *Chem. Res. Toxicol.* **2021**, *34* (9), 2100–2109. DOI: 10.1021/acs.chemrestox.1c00182
(46) Endo, S.; Escher, B. I.; Goss, K. U. Capacities of Membrane Lipids to Accumulate Neutral Organic Chemicals. *Environ. Sci. Technol.* **2011**, *45* (14), 5912–5921. DOI: 10.1021/es200855w
(47) Endo, S.; Bauerfeind, J.; Goss, K.-U. Partitioning of Neutral Organic Compounds to Structural Proteins. *Environ. Sci. Technol.* **2012**, *46* (22), 12697–12703. DOI: 10.1021/es303379y
(48) Escher, B. I.; Schwarzenbach, R. P. Partitioning of substituted phenols in liposome-water, biomembrane-water, and octanol-water systems. *Environ. Sci. Technol.* **1996**, *30* (1), 260–270. DOI: 10.1021/es9503084

(49) Kwon, J. H.; Liljestrand, H. M.; Katz, L. E. Partitioning of moderately hydrophobic endocrine disruptors between water and synthetic membrane vesicles. *Environ. Toxicol. Chem.* **2006**, *25* (8), 1984–1992. DOI: 10.1897/05-550r.1
(50) Endo, S.; Goss, K. U. Serum Albumin Binding of Structurally Diverse Neutral Organic Compounds: Data and Models. *Chem. Res. Toxicol.* **2011**, *45* (24), 2293–2301. DOI: 10.1021/es200855w
(51) Huchthausen, J.; Braasch, J.; Escher, B. I.; König, M.; Henneberger, L. Effects of Chemicals in Reporter Gene Bioassays with Different Metabolic Activities Compared to Baseline Toxicity. *Chem. Res. Toxicol.* **2024**, *37* (5), 744–756. DOI: 10.1021/acs.chemrestox.4c00017
(52) Qin, W.; Henneberger, L.; Glüge, J.; König, M.; Escher, B. I. Baseline Toxicity Model to Identify the Specific and Nonspecific Effects of Per- and Polyfluoroalkyl Substances in Cell-Based Bioassays. *Environ. Sci. Technol.* **2024**, *58* (13), 5727–5738. DOI: 10.1021/acs.est.3c09950
(53) Maeder, V.; Escher, B. I.; Scheringer, M.; Hungerbuhler, K. Toxic ratio as an indicator of the intrinsic toxicity in the assessment of persistent, bioaccumulative, and toxic chemicals. *Environ. Sci. Technol.* **2004**, *38* (13), 3659–3666. DOI: 10.1021/es0351591
(54) Altenburger, R.; Boedeker, W.; Faust, M.; Grimme, L. H. Regulations for combined effects of pollutants: consequences from risk assessment in aquatic toxicology. *Food. Chem. Toxicol.* **1996**, *34* (11-12), 1155–1157.
(55) Mustieles, V.; Arrebola, J. P. How polluted is your fat? What the study of adipose tissue can contribute to environmental epidemiology. *Journal of Epidemiology and Community Health* **2020**, *74* (5), 401–407. DOI: 10.1136/jech-2019-213181
(56) Escher, B.; Neale, P.; Leusch, F. *Bioanalytical tools in water quality assessment, second edition, www.iwapublishing.com/books/9781789061970/bioanalytical-tools-water-quality-assessment-2nd-edition*; IWA Publishing, 2021. DOI: 10.2166/9781789061987.
(57) Lee, J.; Escher, B. I.; Scholz, S.; Schlichting, R. Inhibition of neurite outgrowth and enhanced effects compared to baseline toxicity in SH-SY5Y cells. *Arch. Toxicol.* **2022**, *96*, 1039–1053. DOI: 10.1007/s00204-022-03237-x
(58) Lee, J.; Huchthausen, J.; Schlichting, R.; Scholz, S.; Henneberger, L.; Escher, B. I. Validation of an SH-SY5Y Cell–Based Acetylcholinesterase Inhibition Assay for Water Quality Assessment. *Environ. Toxicol. Chem.* **2022**, *41* (12), 3046–3057. DOI: 10.1002/etc.5490
(59) Louisse, J.; Gönen, S.; Rietjens, I. M.; Verwei, M. Relative developmental toxicity potencies of retinoids in the embryonic stem cell test compared with their relative potencies in in vivo and two other in vitro assays for developmental toxicity. *Toxicology Letters* **2011**, *203* (1), 1–8. DOI: 10.1016/j.toxlet.2011.02.012
(60) Walker, L. M.; Sparks, N. R.; Puig-Sanvicens, V.; Rodrigues, B.; Zur Nieden, N. I. An Evaluation of Human Induced Pluripotent Stem Cells to Test for Cardiac Developmental Toxicity. *Int J Mol Sci* **2021**, *22* (15), 8114. DOI: 10.3390/ijms22158114
(61) Braun, G.; Herberth, G.; Krauss, M.; König, M.; Wojtysiak, N.; Zenclussen, A. C.; Escher, B. I. Neurotoxic mixture effects of chemicals extracted from blood of pregnant women. *Science* **2024**, *386* (6719), 301–309. DOI: 10.1126/science.adq0336
(62) Lee, J.; Schlichting, R.; König, M.; Scholz, S.; Krauss, M.; Escher, B. I. Monitoring Mixture Effects of Neurotoxicants in Surface Water and Wastewater Treatment Plant

Effluents with Neurite Outgrowth Inhibition in SH-SY5Y Cells. *ACS Environmental Au* **2022**, *2* (6), 523–535. DOI: 10.1021/acsenvironau.2c00026
(63) Neale, P. A.; O'Brien, J. W.; Glauch, L.; König, M.; Krauss, M.; Mueller, J. F.; Tscharke, B.; Escher, B. I. Wastewater Treatment Efficacy Evaluated with In Vitro Bioassays. *Water Res X* **2020**, *9*, 100072. DOI: 10.1016/j.wroa.2020.100072
(64) Neale, P.; Feliers, C.; Glauch, L.; Lecarpentier, C.; Schlichting, R.; Thibert, S.; Escher, B. Application of in vitro bioassays for water quality monitoring in three drinking water treatment plants using different treatment processes including biological treatment, nanofiltration and ozonation coupled with disinfection. *Environ. Sci.: Water Res. Technol.* **2020**, *6* (9), 2444–2453. DOI: doi.org/10.1039/C9EW00987F
(65) Neale, P. A.; Escher, B. I. Co-extracted dissolved organic carbon has a suppressive effect on the acetylcholinesterase inhibition assay. *Environ. Toxicol. Chem.* **2013**, *32* (7), 1526–1534. DOI: DOI: 10.1002/etc.2196
(66) Finckh, S.; Buchinger, S.; Escher, B. I.; Hollert, H.; König, M.; Krauss, M.; Leekitratanapisan, W.; Schiwy, S.; Schlichting, R.; Shuliakevich, A.; Brack, W. Endocrine disrupting chemicals entering European rivers: Occurrence and adverse mixture effects in treated wastewater. *Environ. Internat.* **2022**, *170*, 107608. DOI: 10.1016/j.envint.2022.107608
(67) Neale, P. A.; Escher, B. I.; de Baat, M. L.; Enault, J.; Leusch, F. D. L. Effect-Based Trigger Values Are Essential for the Uptake of Effect-Based Methods in Water Safety Planning. *Environ. Toxicol. Chem.* **2023**, *42* (3), 714–726, https://doi.org/10.1002/etc.5544. DOI: 10.1002/etc.5544
(68) Escher, B. I.; Ait-Aissa, S.; Behnisch, P. A.; Brack, W.; Brion, F.; Brouwer, A.; Buchinger, S.; Crawford, S.; Hamers, T. H. M.; Hettwer, K.; Hilscherova, K.; Hollert, H.; Kase, R.; Kienle, C.; Legradi, J.; Tuerk, J.; van der Oost, R.; Vermeirssen, E.; Neale, P. A. Effect-based trigger values for *in vitro* and *in vivo* bioassays performed on surface water extracts supporting the environmental quality standards (EQS) of the European Water Framework Directive. *Science of the Total Environment* **2018**, *628-629*, 748–765. DOI: 10.1016/j.scitotenv.2018.01.340
(69) ECHA. ECHA chemicals database (ECHACHEM). European Chemicals Agency. **2025**, https://chem.echa.europa.eu/ accessed 2025/2012/2016).
(70) US EPA. CompTox Chemicals Dashboard. **2025**, https://comptox.epa.gov/dashboard/ accessed on 2025/2004/2012.
(71) Williams, A. J.; Lambert, J. C.; Thayer, K.; Dorne, J.-L. C. M. Sourcing data on chemical properties and hazard data from the US-EPA CompTox Chemicals Dashboard: A practical guide for human risk assessment. *Environ. Internat.* **2021**, *154*, 106566. DOI: https://doi.org/10.1016/j.envint.2021.106566
(72) WFD. *Common Implementation Strategy for the Water Framework Directive (2000/60/EC). Technical Guidance for Deriving Environmental Quality Standards*; WG E(9) –10-03e – TGD-EQS WG E(9) –10-03e – TGD-EQS 2010.
(73) European Food Safety Agency (EFSA). *The EFSA Comprehensive European Food Consumption Database*. 2024. https://www.efsa.europa.eu/en/data-report/food-consumption-data (accessed 2025/12/17) (accessed.
(74) Noyes, P. D.; Friedman, K. P.; Browne, P.; Haselman, J. T.; Gilbert, M. E.; Hornung, M. W.; Barone, S.; Crofton, K. M.; Laws, S. C.; Stoker, T. E.; Simmons, S. O.; Tietge, J. E.; Degitz, S. J. Evaluating Chemicals for Thyroid Disruption: Opportunities

and Challenges with in Vitro Testing and Adverse Outcome Pathway Approaches. *Environ. Health Perspect.* **2019**, *127* (9), CID: 095001. DOI: 10.1289/ehp5297
